# IFNγ, and to a Lesser Extent TNFα, Provokes a Sustained Endothelial Costimulatory Phenotype

**DOI:** 10.3389/fimmu.2021.648946

**Published:** 2021-04-15

**Authors:** Nicole M. Valenzuela

**Affiliations:** Department of Pathology and Laboratory Medicine, University of California, Los Angeles, Los Angeles, CA, United States

**Keywords:** endothelial cell (EC), costimulatory, allogeneic, antigen presentation, vascular

## Abstract

**Background:**

Vascular endothelial cells (EC) are critical for regulation of local immune responses, through coordination of leukocyte recruitment from the blood and egress into the tissue. Growing evidence supports an additional role for endothelium in activation and costimulation of adaptive immune cells. However, this function remains somewhat controversial, and the full repertoire and durability of an enhanced endothelial costimulatory phenotype has not been wholly defined.

**Methods:**

Human endothelium was stimulated with continuous TNFα or IFNγ for 1-48hr; or primed with TNFα or IFNγ for only 3hr, before withdrawal of stimulus for up to 45hr. Gene expression of cytokines, costimulatory molecules and antigen presentation molecules was measured by Nanostring, and publicly available datasets of EC stimulation with TNFα or IFNγ were leveraged to further corroborate the results. Cell surface protein expression was detected by flow cytometry, and secretion of cytokines was assessed by Luminex and ELISA. Key findings were confirmed in primary human endothelial cells from 4-6 different vascular beds.

**Results:**

TNFα triggered mostly positive immune checkpoint molecule expression on endothelium, including CD40, 4-1BB, and ICOSLG but in the context of only HLA class I and immunoproteasome subunits. IFNγ promoted a more tolerogenic phenotype of high PD-L1 and PD-L2 expression with both HLA class I and class II molecules and antigen processing genes. Both cytokines elicited secretion of IL-15 and BAFF/BLyS, with TNFα stimulated EC additionally producing IL-6, TL1A and IL-1β. Moreover, endothelium primed for a short period (3hr) with TNFα mostly failed to alter the costimulatory phenotype 24-48hr later, with only somewhat augmented expression of HLA class I. In contrast, brief exposure to IFNγ was sufficient to cause late expression of antigen presentation, cytokines and costimulatory molecules. In particular HLA class I, PD-1 ligand and cytokine expression was markedly high on endothelium two days after IFNγ was last present.

**Conclusions:**

Endothelia from multiple vascular beds possess a wide range of other immune checkpoint molecules and cytokines that can shape the adaptive immune response. Our results further demonstrate that IFNγ elicits prolonged signaling that persists days after initiation and is sufficient to trigger substantial gene expression changes and immune phenotype in vascular endothelium.

## Introduction

The blood endothelium is a highly active sensor that integrates multiple signals to control physiological vascular and immune function. It is well established that cytokine activation of blood endothelial cells (EC) triggers upregulation of numerous chemokines and adhesion molecules that promote adherence and extravasation of leukocytes. Recently, the capacity of lymphatic endothelium, and particularly lymph node-associated lymphatics, to also control innate and adaptive immune responses has been an increased subject of inquiry (1). Despite significant attention to the vascular endothelial cell’s interaction with lymphocytes at the level of adherence, it has been a matter of contention whether blood EC possess sufficient costimulatory capacity to modulate the adaptive immune compartment beyond simply “recruiting” it to sites of injury.

Activation of naïve T cells requires engagement of the TCR by MHC class I or class II with cognate peptide and a specific repertoire of antigen-independent costimulatory molecules. Memory T cells are less reliant on costimulation but also require specific signals. Professional antigen presenting cells (APC) able to fulfill this capacity are of the hematopoietic lineage and include monocytes, macrophages, dendritic cells and B cells.

Like all nucleated cells, endothelial cells constitutively express MHC class I molecules, which is augmented by exposure to TNFα or interferons (IFN). Early experiments confirmed that allogeneic endothelium could activate bulk CD8 T cells (2), in a manner requiring cell-cell contact (3). In addition, human microvascular EC from at least some vascular beds may express HLA class II *in situ* (4) and respond to type II IFN by upregulation of MHC class II molecules. St. Louis et al. (5) described that IFNγ-stimulated EC could trigger partial helper T cell responses and the authors hypothesized that “costimulatory deficient” endothelial cells could contribute to antigen-selective peripheral recruitment but not T cell proliferation. Subsequently it has been shown that although EC lack the capacity to activate naïve T cells, they can enhance activation of memory T cells (6, 7). It was proposed that endothelial antigen presentation may function to selectively recruit antigen-specific T cells (8, 9). Thus, endothelial immune functions, such as support of T cell activation, proliferation and/or differentiation, are continuing to be elucidated. The emerging paradigm is that EC can function as semi-professional or conditional antigen presenting cells, possessing a restricted repertoire of costimulatory molecules that can alter activation of some T cell subsets (10). Yet, the full endothelial collection of immune molecules that may shape the adaptive immune response has not been defined, particularly which repertoire is expressed under which inflammatory conditions (i.e. the context specific patterns of presentation).

Resolution of inflammation and attenuation of the immune response are critical to curb inappropriate chronic inflammation. Phased activation of endothelial cells is likely to play a role in terminating peripheral inflammation, certainly at the level of recruitment (11) but possibly also throughout effects on the adaptive immune compartment. Importantly, it is not known for how long after cytokine stimulation endothelial cells maintain an enhanced costimulatory phenotype.

In order to provide a comprehensive characterization of endothelial capacity to modulate the adaptive immune response, we focused on molecules that could fulfill the three main signals required for T cell activation: MHC/antigen presentation; cytokines; and costimulatory molecules. We examined multiple endothelial cell types and leveraged public datasets to provide several lines of evidence—both mRNA and protein, across diverse vascular beds of origin—for the expression or absence of these molecules in the endothelial compartment. We compared Th1 cytokines TNFα and IFNγ stimulation of human endothelial cells, assessing baseline and inducible expression of 76 cytokines, antigen presentation and costimulatory molecules known to affect T cell activation. Next, we assessed how durable these alterations were using prime-withdrawal experiments. Our results reveal overlapping and distinct antigen presenting phenotypes after TNFα and IFNγ. Moreover, IFNγ exerted an enduring shift in endothelial cell phenotype days after cytokine has been withdrawn, with long-term enhanced expression of HLA class I and class II, cytokine and coinhibitory molecules. Although the main effector molecules induced by IFNγ appeared as a late phase response, only 3hr of direct exposure to IFNγ was needed to provoke a prolonged endothelial cell phenotype change. Our results provide broader insight into the arsenal of antigen presentation molecules employed by endothelial cells, and particularly highlight the long-lasting effects of IFNγ on endothelial cell immune function.

## Materials and Methods

### Cells and Reagents

All reagents and cells, and their sources, can be found in **Supplemental Tables 1** and **2**.

HMEC-1 immortalized human dermal microvascular endothelial cells were obtained from ATCC. Primary human aortic endothelial cells (HAEC), pulmonary artery (HPAEC), coronary artery (HCAEC), cardiac microvascular, lung microvascular (HCMVEC), dermal microvascular (HDMVEC), renal endothelial cells (HRGEC) and liver endothelial cells (HLSEC) were obtained from commercial sources (see **Supplemental Table 2**). At least three different HAEC donors were tested. EC were cultured on gelatin-coated, tissue culture treated plates to confluence in complete ECM medium (PromoCell, #C-22020). Cells were then stimulated with cytokines diluted in M199 supplemented with 10% heat-inactivated fetal bovine serum (FBS). Human TNFα was obtained from Sigma (#H8916); human IFNγ was from R&D Systems (#285IF).

### Analysis of Public Datasets

Publicly deposited microarray and RNA-Seq data for TNFα and IFNγ stimulated endothelial cells were accessed from NCBI and analyzed in GEO2R (https://www.ncbi.nlm.nih.gov/geo/geo2r/), or downloaded and expression values graphed in Prism. Datasets used were GSE27870 [HUVEC, n=3, TNFα 1-6hr (12)], GSE144810 [HUVEC, n=39, TNFα 24hr (13)], GSE19082 (HMEC, IFNγ 2hr, unpublished), GDS2516/GSE3920 [HUVEC, n=3-5, IFNγ 5hr (14)], GSE6092 (HUVEC, IFNγ 8hr (15), GSE106524 [primary human lung microvascular endothelial cells, IFNγ 3hr, 6hr, 24hr, n=3-4 (unpublished)].

### Prime-Rest Experiments

For transcript changes, endothelial cells (HMEC-1) and primary endothelial cells were stimulated with TNFα (20ng/mL) or IFNγ (200U/mL) diluted in M199+10% FBS for 1hr, 3hr, 6hr, 18hr and 24hr. For protein changes, primary endothelial cells (HAEC) were stimulated with TNFα (20ng/mL) or IFNγ (200U/mL) for 3hr, 6hr, 18hr, 24hr and 40hr. In prime-withdrawal conditions, endothelial cells were primed with TNFα or IFNγ for 3hr, then medium was removed, cells were washed, and fresh M199+10% FBS without cytokine was added for the remainder of the experiment. Controls were treated and tested in parallel for each time point, i.e. when EC were primed for 3hr and then withdrawn for an additional 3hr, a parallel time matched 6hr continuous condition was also tested. All time points ended together, and cell material was collected for analysis concurrently.

### Targeted Gene Expression Analysis

Stimulated endothelial cells were detached with Accutase (Sigma-Aldrich, #A6964), pelleted and resuspended in RLT Buffer (Qiagen) at 1μL/6500 cells. mRNA counts were measured by Nanostring (Human Immunology Panel v2.0, Nanostring Technologies) and analyzed in NCounter software. mRNA counts were normalized against internal and housekeeping controls. Normalized counts ≥250 were considered positive, and genes were considered changed if the counts differed by 50% (i.e. 1.5-fold) or more compared with baseline.

### Flow Cytometry

Flow cytometry panel details are provided in **Supplemental Table 3**. Cell surface expression of proteins was measured by multiparameter flow cytometry staining for CD40-Alexa Fluor 488 (Biolegend #334318), OX40L-PE (Biolegend #326307), 4-1BB-APC (Biolegend #309809), B7-H3-PerCP/Cy5.5 (Biolegend #351010), HLA-ABC-Brilliant Violet 510 (Biolegend #311436), HLA-DR/DP/DQ2-Brilliant Violet 421 (Biolegend #307636, clone L243) and ICOSL-PE/Cy7 (Biolegend #309410); CD273/PD-L2-APC (Biolegend #345507), PD-L1-FITC (Biolegend #374509). All antibodies were used at 5μL per 100μL staining volume diluted in FACS buffer (2% heat inactivated FBS in PBS without Ca^2+^ or Mg^2+^), except for HLA-ABC which was used at 2.5μL per 100μL based on validation experiments. Fluorescence was acquired on a BD Fortessa flow cytometer (BD Biosciences) and analyzed in FlowJo software (BD Biosciences).

### Cytokine and Chemokine Measurements

Conditioned medium was collected from flow cytometry and mRNA experiments and immediately stored in low protein binding tubes or multi-well plates at or below -20⁰C. Secreted cytokines in the supernatants were measured by ELISA (Human BAFF Quantikine ELISA R&D Systems #DBLYS0B; Human IL-15 Quantikine ELISA, R&D Systems #D1500) and Luminex (IL-6, IL-15, Milliplex MAP Human Cytokine/Chemokine 38-Plex panel, Millipore Sigma).

### Statistical Analyses

Heat maps were generated in MORPHEUS (Broad Institute, https://software.broadinstitute.org/morpheus/). Data were graphed in Prism (GraphPad Software). Statistical differences between groups were calculated by unpaired or paired t test, or one-way ANOVA or two-way ANOVA followed by Fisher’s LSD in Prism, as indicated in the figure legends.

## Results

T cells require three main signals for activation by an APC: cell surface antigen presentation in MHC/HLA; a specific repertoire of secreted cytokines; and costimulatory or coinhibitory ligands. We analyzed the kinetics and patterns of endothelial expression of each of these types of effector molecules.

For activation with TNFα, we analyzed two public transcriptome datasets in HUVEC, one with hourly TNFα stimulation from 1-6hr [GSE27870, n=3] and another of 39 donors stimulated with TNFα for 24hr [GSE144810]. For IFNγ stimulation, we analyzed publicly available datasets of IFNγ-exposed EC for 2hr [GSE19082, HMEC, n=4] 5hr [GSE3920, HUVEC, n=5], or 3hr, 6hr, and 24hr [GSE106524, HLMVEC, n=3-4]. In addition, we generated our own dataset of an extended transcriptional time course of TNFα and IFNγ stimulation (1-24hr) and also confirmed these results at the protein level in primary human endothelium from diverse vascular beds of origin.

### TNFα and IFNγ Induce Expression of More Than 40 Genes Involved in Antigen Presentation and Modification of Adaptive Immunity

**Figure 1:**
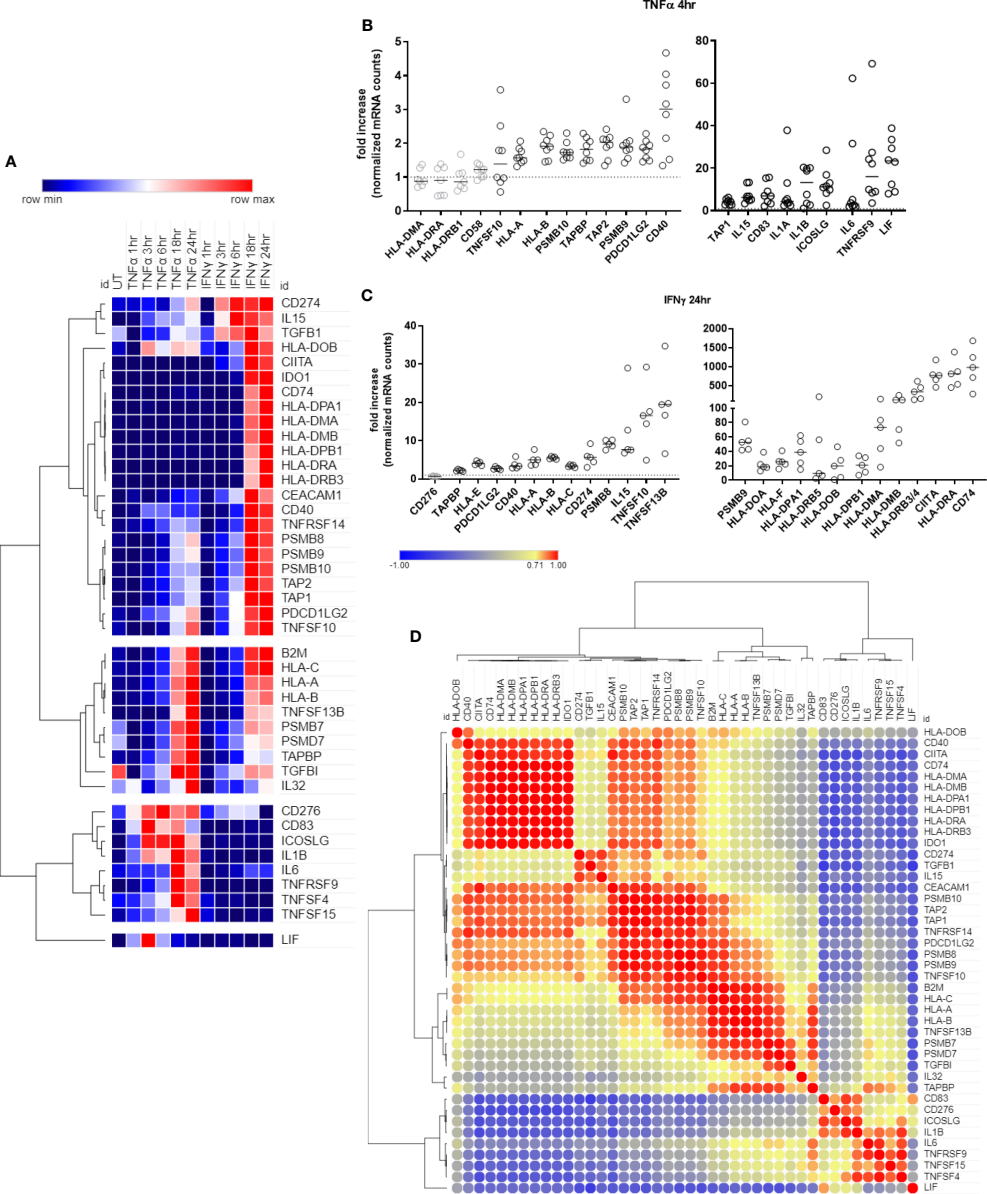
Time course of antigen presentation, costimulatory molecule and cytokine genes after TNFα or IFNγ stimulation. **(A)**. HMEC-1 endothelial cells were stimulated with TNFα (20ng/mL) or IFNγ (200U/mL) for 1hr, 3hr, 6hr, 18hr or 24hr. mRNA counts were measured by Nanostring and normalized to housekeeping genes and controls. Heat map shows relative gene expression over time, with normalization across genes/rows. Hierarchical clustering of rows is by one minus Pearson correlation. **(B)**. Primary human endothelial cells (HAEC, HCAEC, HCMVEC, HPAEC, HPMVEC, HDMVEC, HRGEC, HLSEC) were stimulated with TNFα (20ng/mL) for 4hr, and transcript levels were measured by Nanostring. The fold change in normalized mRNA counts was calculated compared to untreated conditions for each EC type (n=8). **(C).** Primary human endothelial cells (HPAEC, HRGEC, HPMVEC, HLSEC) were stimulated with IFNγ (200U/mL) for 24hr, and transcript levels were measured by Nanostring. The fold change in normalized mRNA counts was calculated compared to untreated conditions for each EC type. (n=4). **(D).** Similarity of costimulatory, cytokine and antigen presentation gene expression across untreated, TNFα and IFNγ treated endothelial cells. Heat map similarity matrix shows temporal and context dependent correlation. Hierarchical cluster analysis and the similarity matrix were generated by MORPHEUS using Pearson correlation values.

We first characterized temporal transcriptional changes in human endothelial cells, after TNFα and IFNγ stimulation. Endothelial cells (HMEC-1) were left untreated or stimulated with TNFα (20ng/mL) or IFNγ (200U/mL) for 1hr, 3hr, 6hr, 18hr or 24hr. Forty-two genes encoding antigen presentation, costimulation/coinhibition molecules or cytokines were upregulated in EC by TNFα, IFNγ or both (**Figure 1A**, **Supplemental Table 4**). On the other hand, 35 cytokines, antigen presentation and costimulatory genes were either not found to be expressed by endothelium or were expressed but not induced by TNFα or IFNγ (**Supplemental Table 5**).

Ten genes were increased by both TNFα and IFNγ to a comparable extent; 11 genes were more highly upregulated by IFNγ than TNFα; 12 genes were IFNγ responsive but unaltered by TNFα; and 9 genes were predominantly TNFα but not IFNγ inducible (**Figure 1A**, **Supplemental Table 4**). Nearly all antigen presentation genes under IFNγ control occurred as late phase events, at 18hr or 24hr after stimulation, while many TNFα-induced genes appeared earlier. Based on these kinetics, we confirmed mRNA induction of cytokines and costimulatory molecules in human primary endothelial cells from 6 other vascular beds (coronary artery, cardiac microvascular, pulmonary artery, lung microvascular, renal glomerular and dermal microvascular), after stimulation with TNFα 4hr (**Figure 1B**) or IFNγ 24hr (**Figure 1C**). Fold change compared to an untreated parallel condition is presented in **Figures 1B, C**; discrete mRNA counts for each cell type are provided in **Supplemental Figures S4–S10**.

In order to understand the temporal and stimulus dependent co-expression of genes, we analyzed the transcript data over time and under both TNFα and IFNγ conditions using a similarity matrix (**Figure 1D**). Expression of *IDO1* and *CD40* was strongly correlated with concurrent expression of HLA class II genes (*CIITA*, *CD74*, *HLA-DRB*). In addition, endothelial expression of immunoproteasome genes (*PSMB8, PSMB9, PSMB10*) was somewhat correlated, unsurprisingly, with HLA class I expression, but more strongly correlated with *PDCD1LG2* (PD-L2) and *TNFRSF14* (HVEM). Other strongly correlated gene clusters included *IL6*, *TNFRSF9* (4-1BB), *TNFSF15* (TL1A/VEGI), and *TNFSF4* (OX40L); and *CD83*, *CD276* (B7-H3), *ICOSLG* and *IL1B*.

### Time Course of TNFα-Induced Cytokines, Antigen Presentation Machinery and Costimulatory Molecules

**Figure 2:**
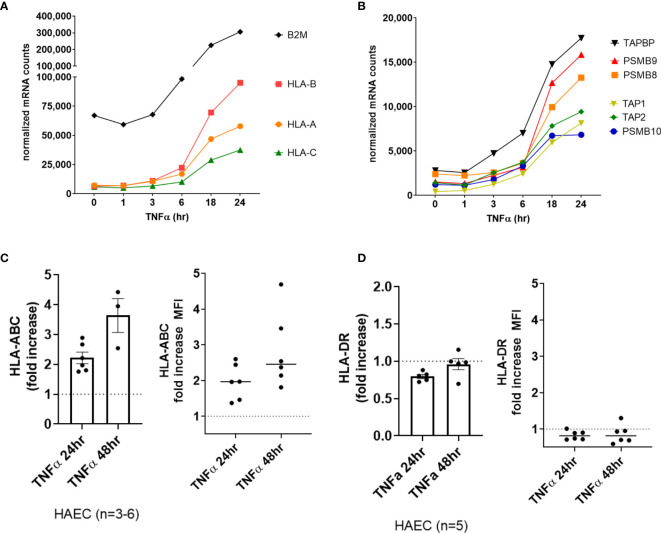
TNFα induced endothelial expression of HLA class I, peptide presentation and immunoproteasome genes. **(A)**. HMEC-1 endothelial cells were stimulated with TNFα (20ng/mL) for 1hr, 3hr, 6hr, 18hr or 24hr. Time course of TNFα induced HLA class I transcripts in HMEC-1. One representative experiment is shown. Results are presented as absolute number of normalized mRNA counts for each gene, normalized to housekeeping controls. **(B)** Time course of TNFα induced antigen presentation transcripts in HMEC-1. One representative experiment is shown. Results are presented as absolute number of normalized mRNA counts for each gene, normalized to housekeeping controls. **(C, D)** Primary human endothelial cells (HAEC, HCAEC, HCMVEC, HPAEC, HPMVEC, HLSEC) were stimulated with TNFα for 24hr or 48hr. Cell surface HLA-ABC **(C)** and HLA-DR **(D)** protein was stained and detected by flow cytometry. Results are presented as the fold increase in MFI compared with untreated conditions (n=3-6 HAEC, left panel; n=6 EC from diverse vascular beds, right panel).

Classical HLA genes *HLA-A* and *B2M* (β2-microglobulin) did not increase significantly in the first 6hr of TNFα treatment, yet the chaperone gene *TAP1* and the immunoproteasome gene *PSMB10* were significantly elevated as early as 3-4hr (**Supplemental Figures S1A–D**, GSE27870 in HUVEC n=3). In our own extended time course, HLA class I transcripts (*HLA-A*, *HLA-B*, *HLA-C*) (**Figure 2A**) as well as class I antigen presentation accessory molecules and immunoproteasome subunits (*ERAP2*, *TAP1*, *TAP2*, *TAPBP*, *PSMB8*, *PSMB9*, *PSMB10*) were increased by TNFα after 6hr, increasing to 24hr (**Figure 2B**). At the protein level on primary aortic endothelium, HLA class I was constitutively expressed and further increased by TNFα as early as 18hr, rising through 48hr (**Figure 2C**, left panel). Similar responses were seen across endothelial cells from 6 diverse vascular beds (**Figure 2C**, right panel; **Supplemental Figure S3**). TNFα also upregulated endothelial non-classical HLA-E and HLA-F expression (2.0-fold at 18hr, *data not shown*), while HLA class II and related genes were not affected in primary aortic endothelium (n=5) and endothelium from other vascular beds (n=6**)** (*data not shown* and **Figure 2D**, **Supplemental Figure S3**).

**Figure 3:**
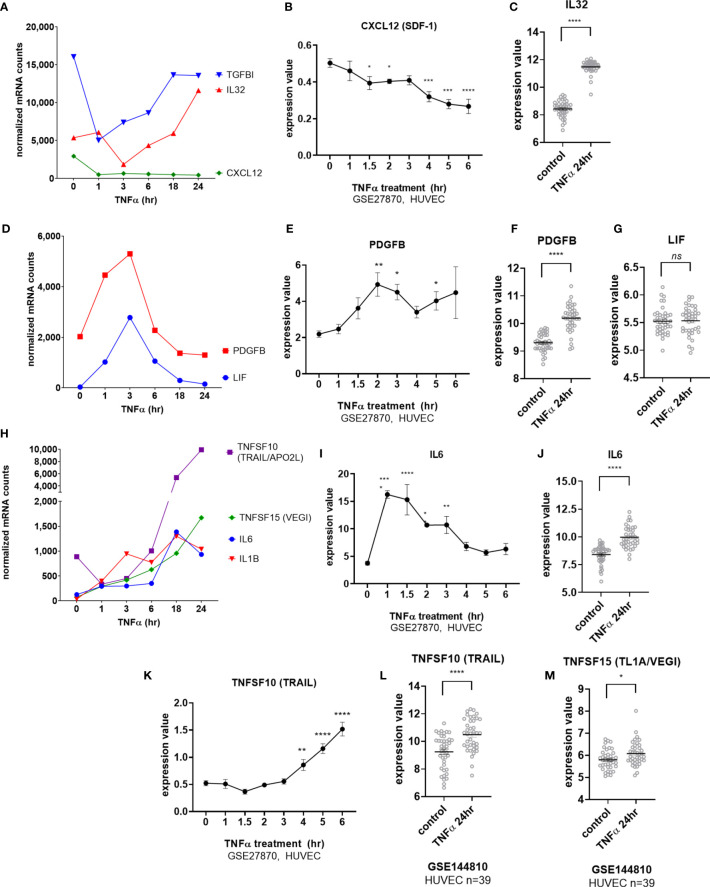
TNFα induced endothelial expression of cytokines. **(A)** HMEC-1 endothelial cells were stimulated with TNFα (20ng/mL) for 1hr, 3hr, 6hr, 18hr or 24hr. Time course of TNFα induced cytokines in HMEC-1. Time course of TNFα-suppressed cytokine mRNA for *TGFBI*, *IL32* and *CXCL12* in HMEC-1. One representative experiment is shown. Results are presented as absolute number of normalized mRNA counts for each gene, normalized to housekeeping controls. **(B)**. Expression values of *CXLC12* from GSE27870 are plotted, for HUVEC stimulated with TNFα for 1hr, 1.5hr, 2hr, 3hr, 4hr, 5hr and 6hr. Values at each time point were compared to baseline (0hr) by One way ANOVA followed by uncorrected Fisher’s LSD t test. *p < 0.05; ***p < 0.001; ****p < 0.0001. Results are shown as mean ± SEM (n=3). **(C)** Expression values of *IL32* from GSE144810 are plotted, comparing HUVEC (n=39) left untreated or stimulated with TNFα for 24hr. Value distributions were compared by unpaired t test. Results are presented with each measured value, and the line at the median. ****p < 0.0001. **(D)** Time course of TNFα early transient-induced cytokine mRNA for *PDGFB* and *LIF* in HMEC-1. One representative experiment is shown. Results are presented as absolute number of normalized mRNA counts for each gene, normalized to housekeeping controls. **(E)** Expression values of *PDGFB* from GSE27870 are plotted, for HUVEC stimulated with TNFα for 1hr, 1.5hr, 2hr, 3hr, 4hr, 5hr and 6hr. Values at each time point were compared to baseline (0hr) by One way ANOVA followed by uncorrected Fisher’s LSD t test. *p < 0.05; **p < 0.01. Results are shown as mean ± SEM (n=3). **(F)** Expression values of *PDGFB* from GSE144810 are plotted, comparing HUVEC (n=39) left untreated or stimulated with TNFα for 24hr. Value distributions were compared by unpaired t test. Results are presented with each measured value, and the line at the median. ****p < 0.0001. **(G)** Expression values of *LIF* from GSE144810 are plotted, comparing HUVEC (n=39) left untreated or stimulated with TNFα for 24hr. Value distributions were compared by unpaired t test. Results are presented with each measured value, and the line at the median. ****p < 0.0001. **(H)** Time course of TNFα late phase induced cytokine mRNA for *TNFSF10, TNFSF15, IL6* and *IL1B* in HMEC-1. One representative experiment is shown. Results are presented as absolute number of normalized mRNA counts for each gene, normalized to housekeeping controls. **(I)** Expression values of *IL6* from GSE27870 are plotted, for HUVEC stimulated with TNFα for 1hr, 1.5hr, 2hr, 3hr, 4hr, 5hr and 6hr. Values at each time point were compared to baseline (0hr) by One way ANOVA followed by uncorrected Fisher’s LSD t test. *p < 0.05; **p < 0.01; ****p < 0.0001. Results are shown as mean ± SEM (n=3). **(J)** Expression values of *IL6* from GSE144810 are plotted, comparing HUVEC (n=39) left untreated or stimulated with TNFα for 24hr. Value distributions were compared by unpaired t test. Results are presented with each measured value, and the line at the median. ****p < 0.0001. **(K)** Expression values of *TNFSF10* from GSE27870 are plotted, for HUVEC stimulated with TNFα for 1hr, 1.5hr, 2hr, 3hr, 4hr, 5hr and 6hr. Values at each time point were compared to baseline (0hr) by One way ANOVA followed by uncorrected Fisher’s LSD t test. **p < 0.01; ****p < 0.0001. Results are shown as mean ± SEM (n=3). **(L)** Expression values of *TNFSF10* from GSE144810 are plotted, comparing HUVEC (n=39) left untreated or stimulated with TNFα for 24hr. Value distributions were compared by unpaired t test. Results are presented with each measured value, and the line at the median. ****p < 0.0001. **(M)** Expression values of *TNFSF15* from GSE144810 are plotted, comparing HUVEC (n=39) left untreated or stimulated with TNFα for 24hr. Value distributions were compared by unpaired t test. Results are presented with each measured value, and the line at the median. *p < 0.05, not significant, ns p > 0.05.

Endothelial cells elaborated interleukins and other cytokines in response to TNFα stimulation. Two cytokines, *CXCL12* (SDF-1) and *IL32*, were constitutively expressed by EC. Tonic *CXCL12* (SDF-1) expression was substantially reduced by TNFα as early as 1hr (**Figures 3A, B**) and remained durably suppressed through 24hr (**Figure 3A**). Constitutive *IL32* mRNA was also decreased in TNFα-activated endothelial cells (at 3hr), but then became elevated above baseline at 24hr (**Figures 3A, C** GSE144810). Raw mRNA counts are shown in **Supplemental Figure S4A, S4D**.

mRNA for the IL-6 family cytokine *LIF* as well as for *PDGFB* was increased early at 3hr but declined rapidly (**Figures 3D, E**). *PDGFB* but not *LIF* expression remained elevated above baseline after 24hr of TNFα exposure (**Figures 3F, G**). mRNA for *IL6*, *IL1B*, *TNFSF15* (TL1A/VEGI) and *TNFSF10* (TRAIL) were detected as early as 3hr (**Figures 3H, I, K**) and continued to increase to 24hr (**Figures 3H, J, L, M**). However, although endothelial cell production of *TNFSF15* (TL1A/VEGI) has been reported before (16), TNFα-induced increases were very modest (**Figures 3H, M** and **Supplemental Figure S4J**).

**Figure 4:**
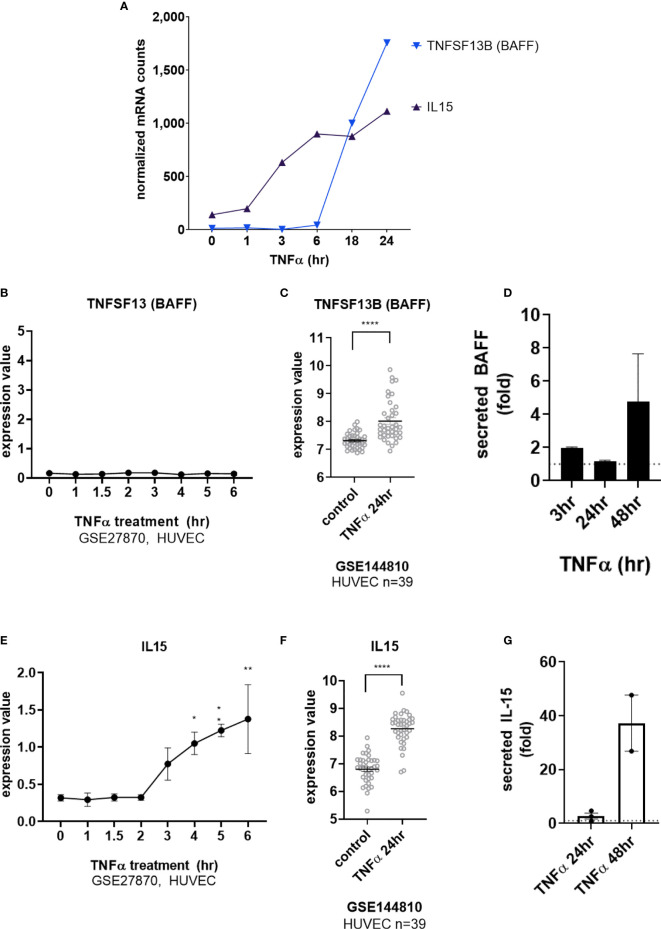
TNFα induces endothelial expression of cytokines IL-15 and BAFF/BLyS. **(A)** HMEC-1 endothelial cells were stimulated with TNFα (20ng/mL) for 1hr, 3hr, 6hr, 18hr or 24hr. Time course of TNFα late phase induced cytokine mRNA for *TNFSF13B* and *IL15α* in HMEC-1. One representative experiment is shown. Results are presented as absolute number of normalized mRNA counts for each gene, normalized to housekeeping controls. **(B)** Expression values of *TNFSF13* from GSE27870 are plotted, for HUVEC stimulated with TNFα for 1hr, 1.5hr, 2hr, 3hr, 4hr, 5hr and 6hr. Values at each time point were compared to baseline (0hr) by One way ANOVA followed by uncorrected Fisher’s LSD t test. Results are shown as mean ± SEM (n=3). **(C)** Expression values of *TNFSF13B* from GSE144810 are plotted, comparing HUVEC (n=39) left untreated or stimulated with TNFα for 24hr. Value distributions were compared by unpaired t test. Results are presented with each measured value, and the line at the median. ****p < 0.0001. **(D)** Primary human aortic endothelial cells (n=4 HAEC donors) were stimulated with TNFα for 3hr, 24hr or 48hr. Supernatants were collected and secreted BAFF protein was measured by ELISA. Results are presented as the fold increase in secreted cytokine compared with conditioned medium from untreated endothelial cells, mean ± SEM. **(E)** Expression values of *IL15* from GSE27870 are plotted, for HUVEC stimulated with TNFα for 1hr, 1.5hr, 2hr, 3hr, 4hr, 5hr and 6hr. Values at each time point were compared to baseline (0hr) by One way ANOVA followed by uncorrected Fisher’s LSD t test. *p < 0.05; **p < 0.01. Results are shown as mean ± SEM (n=3). **(F)** Expression values of *IL15* from GSE144810 are plotted, comparing HUVEC (n=39) left untreated or stimulated with TNFα for 24hr. Value distributions were compared by unpaired t test. Results are presented with each measured value, and the line at the median. ****p < 0.0001. **(G)** Primary human aortic endothelial cells (n=2 donors) were stimulated with TNFα for 24hr or 48hr. Supernatants were collected and secreted IL-15 protein was measured by ELISA. Results are presented as the fold increase in secreted cytokine compared with conditioned medium from untreated endothelial cells, mean ± SEM.

Interestingly, transcripts for *IL15* and *TNFSF13B* (BAFF/BLyS) were also detected after long-term TNFα exposure (**Figure 4A** and **Supplemental Figures S4B, S4E**). *TNFSF13B* (BAFF/BLyS) mRNA did not appear until after 6hr (**Figures 4A, B**, GSE27870 in HUVEC) and was highest at 24hr (**Figures 4A, C**). Limited prior work showed that endothelial cells may produce *TNFSF13B* (BAFF/BLyS), mostly in the context of viral infection or malignancy (17–19). Therefore, we corroborated the increases in mRNA by measuring secreted protein. Indeed, endothelial cells stimulated with TNFα released BAFF/BLyS protein in a delayed manner (**Figure 4D**). BAFF protein secretion from aortic EC was not significantly detected at 3hr or 24hr, but was increased 1.72 ± 0.1 fold at 48hr (from 24.7pg/mL to 65.9pg/mL n=2 donors, **Figure 4D**).

TNFα-induced *IL15* transcript was detected as early as 3hr (**Figures 4A, E**) and increased out to 24hr (**Figures 4A, F**). Because there are only a few reports of endothelial cells as a source of IL-15 (3, 20–23), we verified TNFα-induced protein secretion from primary human endothelial cells. IL-15 secretion was increased 1.85 ± 0.4-fold at 48hr (from 0.125+/-0.053pg/mL to 5.219+/-3.3pg/mL n=2 donors, **Figure 4G** and **S4c**). IL-15 protein was also released from primary endothelial cells activated with TNFα across 6 other vascular beds (1.63 ± 0.14-fold at 24hr, *data not shown*).

**Figure 5:**
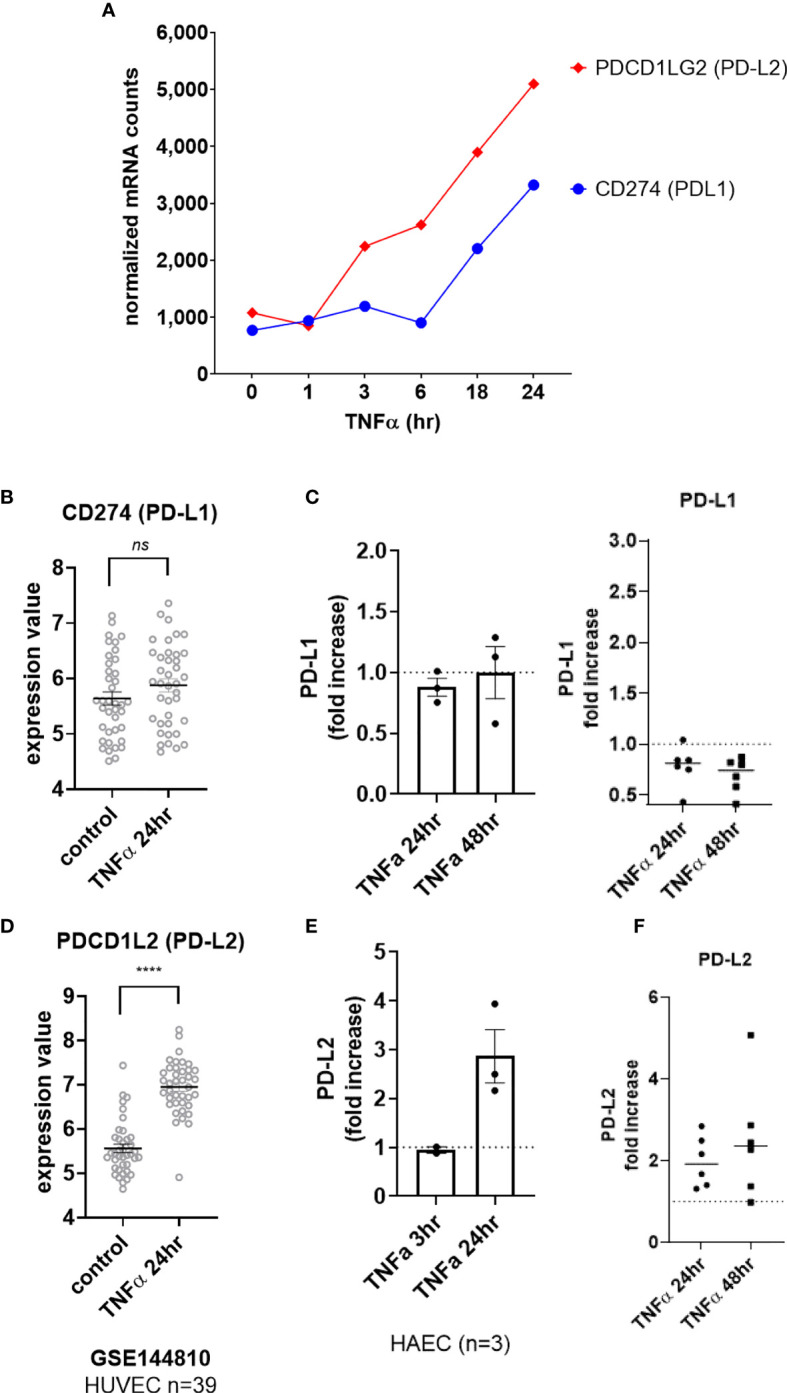
TNFα induced endothelial expression of PD-1 ligands. **(A)** HMEC-1 endothelial cells were stimulated with TNFα (20ng/mL) for 1hr, 3hr, 6hr, 18hr or 24hr. Time course of TNFα induced costimulatory molecule mRNA for *CD274* and *PDCD1LG2* in HMEC-1. One representative experiment is shown. Results are presented as absolute number of normalized mRNA counts for each gene, normalized to housekeeping controls. **(B)** Expression values of *CD274* from GSE144810 are plotted, comparing HUVEC (n=39) left untreated or stimulated with TNFα for 24hr. Value distributions were compared by unpaired t test. Results are presented with each measured value, and the line at the median. **(C)** Primary human endothelial cells (HAEC, HCAEC, HCMVEC, HPAEC, HPMVEC, HLSEC) were stimulated with TNFα for 24hr or 48hr. Cell surface PD-L1 protein was stained and detected by flow cytometry. Results are presented as the fold increase in MFI compared with untreated conditions for each EC type. Left panel shows HAEC (n=3); right panel shows fold change in MFI across EC from different vascular beds (n=6). **(D)** Expression values of *PDCD1L2* from GSE144810 are plotted, comparing HUVEC (n=39) left untreated or stimulated with TNFα for 24hr. Value distributions were compared by unpaired t test. Results are presented with each measured value, and the line at the median. ****p < 0.0001 compared to control. **(E)** Primary human aortic endothelial cells (n=3 donors) were stimulated with TNFα for 3hr or 24hr. Cell surface PD-L1 and PD-L2 were measured by flow cytometry. Results are presented as the fold increase in MFI compared with untreated conditions, mean ± SEM. **(F)** Primary human endothelial cells (HAEC, HCAEC, HCMVEC, HPAEC, HPMVEC, HLSEC) were stimulated with TNFα for 24hr or 48hr. Cell surface PD-L2 protein was stained and detected by flow cytometry. Results are presented as the fold increase in MFI compared with untreated conditions. not significant, ns p > 0.05.

Although we detected a slight increase in transcript for *CD274* (PD-L1) in HMEC-1 (**Figure 5A**), this could not be reproduced in other datasets with primary umbilical vein endothelium (**Figure 5B**) or other primary endothelium (**Supplemental Figure S5A**), nor at the cell surface protein level in aortic endothelium (n=3, **Figure 5C** left panel) or endothelium from other vascular beds (**Figure 5C** right panel, **Supplemental Figures S5B, C, G**). In contrast, TNFα stimulation consistently upregulated *PDCD1LG2* (PD-L2) in HMEC-1, HUVEC, HAEC, HCAEC, HCMVEC, HPAEC, HLMVEC and HRGEC (**Figures 5S, 5D** and **Supplemental Figure S5D**). Moreover, TNFα-induced PD-L2 protein expression was verified on primary human aortic endothelial cells from multiple donors (**Figures 5E** and **Supplemental Figure S5F**), as well as across 6 different vascular beds (mRNA: 1.81 ± 0.29-fold at 4hr, **Figures 1B** and **S5E**; protein: 1.98 ± 0.6-fold at 24hr, 2.50 ± 1.4-fold at 48hr, **Figure 5F**, n=6).

**Figure 6:**
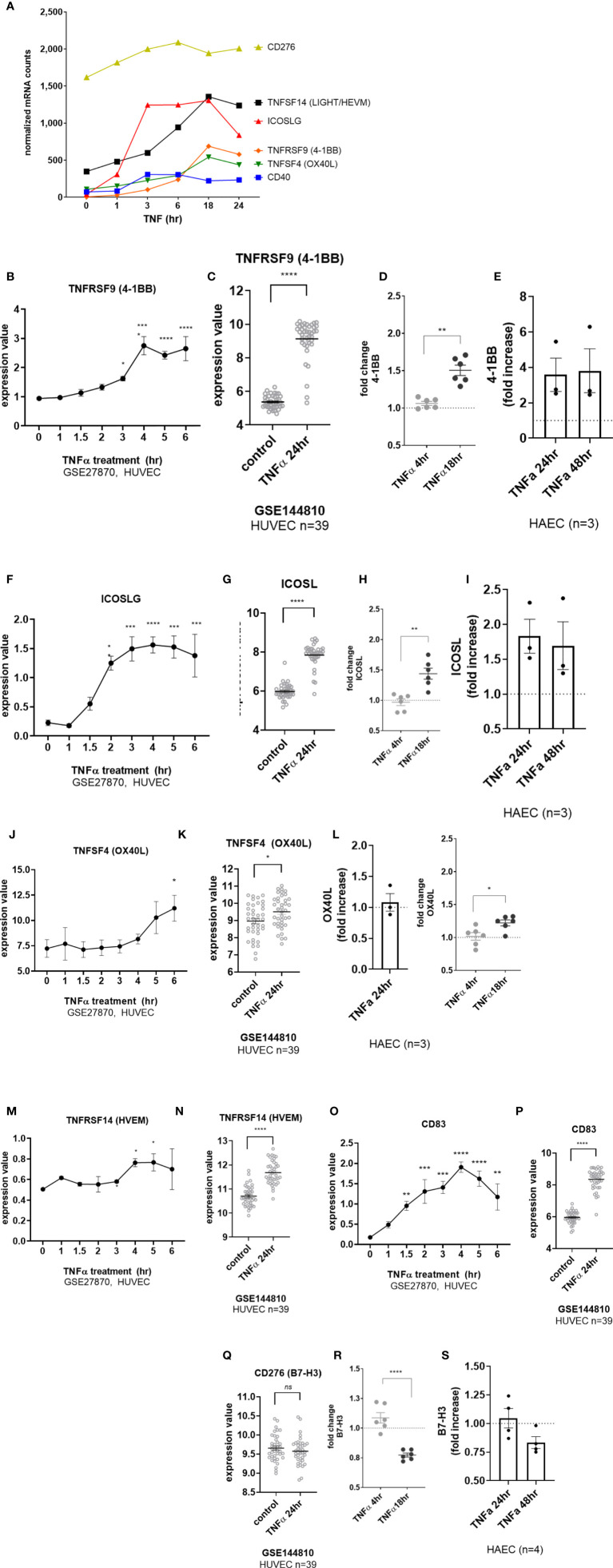
TNFα induced endothelial expression of costimulatory and coinhibitory molecules. **(A)** HMEC-1 endothelial cells were stimulated with TNFα (20ng/mL) for 1hr, 3hr, 6hr, 18hr or 24hr. Time course of TNFα induced costimulatory molecule mRNA for *TNFSF14, ICOSLG, TNFRSF9, TNFSF4, CD40*, and *CD276* in HMEC-1. Results are presented as absolute number of normalized mRNA counts for each gene, normalized to housekeeping controls. **(B)** Expression values of *TNFRSF9* from GSE27870 are plotted, for HUVEC stimulated with TNFα for 1hr, 1.5hr, 2hr, 3hr, 4hr, 5hr and 6hr. Values at each time point were compared to baseline (0hr) by One way ANOVA followed by uncorrected Fisher’s LSD t test. *p < 0.05; ****p < 0.0001. Results are shown as mean ± SEM (n=3). **(C)** Expression values of *TNFRSF9* from GSE144810 are plotted, comparing HUVEC (n=39) left untreated or stimulated with TNFα for 24hr. Value distributions were compared by unpaired t test. Results are presented with each measured value, and the line at the median. ****p < 0.0001 compared to control. **(D)** Primary human (HAEC, HCAEC, HCMVEC, HPAEC, HPMVEC, HLSEC) endothelial cells were treated with TNFα (20ng/mL) for 4hr or 18hr. Cell surface expression of 4-1BB was measured by flow cytometry (n=6). **p < 0.01 by paired t test comparing 4hr to 18hr. **(E)** Primary human aortic endothelial cells (n=3 donors) were stimulated with TNFα for 24hr and 48hr. Cell surface 4-1BB was measured by flow cytometry. Results are presented as average fold increase in MFI of each molecule relative to untreated cells. **(F)** Expression values of *ICOSLG* from GSE27870 are plotted, for HUVEC stimulated with TNFα for 1hr, 1.5hr, 2hr, 3hr, 4hr, 5hr and 6hr. Values at each time point were compared to baseline (0hr) by One way ANOVA followed by uncorrected Fisher’s LSD t test. *p < 0.05; ***p < 0.001; ****p < 0.0001. Results are shown as mean ± SEM (n=3). **(G)** Expression values of *ICOSLG* from GSE144810 are plotted, comparing HUVEC (n=39) left untreated or stimulated with TNFα for 24hr. Value distributions were compared by unpaired t test. Results are presented with each measured value, and the line at the median. ****p < 0.0001 compared to control. **(H)** Primary human (HAEC, HCAEC, HCMVEC, HPAEC, HPMVEC, HLSEC) endothelial cells were treated with TNFα (20ng/mL) for 4hr or 18hr. Cell surface expression of ICOSL was measured by flow cytometry (n=6). **p < 0.01 by paired t test comparing 4hr to 18hr. **(I)** Primary human aortic endothelial cells (n=3 donors) were stimulated with TNFα for 24hr and 48hr. Cell surface ICOSL was measured by flow cytometry. Results are presented as average fold increase in MFI of each molecule relative to untreated cells. **(J)** Expression values of *TNFSF4* from GSE27870 are plotted, for HUVEC stimulated with TNFα for 1hr, 1.5hr, 2hr, 3hr, 4hr, 5hr and 6hr. Values at each time point were compared to baseline (0hr) by One way ANOVA followed by uncorrected Fisher’s LSD t test. *p < 0.05. Results are shown as mean ± SEM (n=3). **(K)** Expression values of *TNFSF4* from GSE144810 are plotted, comparing HUVEC (n=39) left untreated or stimulated with TNFα for 24hr. Value distributions were compared by unpaired t test. Results are presented with each measured value, and the line at the median. *p < 0.01 compared to control. **(L)** Primary human (HAEC, HCAEC, HCMVEC, HPAEC, HPMVEC, HLSEC) endothelial cells were treated with TNFα (20ng/mL) for 4hr or 18hr. Left panel shows relative change in OX40L MFI on HAEC (n=3). Right panel shows fold change in cell surface expression of OX40L measured by flow cytometry across EC from different vascular beds (n=6). *p < 0.05 by paired t test comparing 4hr to 18hr. **(M)** Expression values of *TNFRSF14* from GSE27870 are plotted, for HUVEC stimulated with TNFα for 1hr, 1.5hr, 2hr, 3hr, 4hr, 5hr and 6hr. Values at each time point were compared to baseline (0hr) by One way ANOVA followed by uncorrected Fisher’s LSD t test. *p < 0.05. Results are shown as mean ± SEM (n=3). **(N)** Expression values of *TNFRSF14* from GSE144810 are plotted, comparing HUVEC (n=39) left untreated or stimulated with TNFα for 24hr. Value distributions were compared by unpaired t test. Results are presented with each measured value, and the line at the median. ****p < 0.0001 compared to control. **(O)** Expression values of *CD83* from GSE27870 are plotted, for HUVEC stimulated with TNFα for 1hr, 1.5hr, 2hr, 3hr, 4hr, 5hr and 6hr. Values at each time point were compared to baseline (0hr) by One way ANOVA followed by uncorrected Fisher’s LSD t test. *p < 0.05. Results are shown as mean ± SEM (n=3). **(P)** Expression values of *CD83* from GSE144810 are plotted, comparing HUVEC (n=39) left untreated or stimulated with TNFα for 24hr. Value distributions were compared by unpaired t test. Results are presented with each measured value, and the line at the median. *p < 0.01 compared to control. **(Q)** Expression values of *CD276* from GSE144810 are plotted, comparing HUVEC (n=39) left untreated or stimulated with TNFα for 24hr. Value distributions were compared by unpaired t test. Results are presented with each measured value, and the line at the median. **(R)** Primary human (HAEC, HCAEC, HCMVEC, HPAEC, HPMVEC, HLSEC) endothelial cells were treated with TNFα (20ng/mL) for 4hr or 18hr. Cell surface expression of B7-H3 was measured by flow cytometry (n=6). **p < 0.01 by paired t test comparing 4hr to 18hr. **(S)** Primary human aortic endothelial cells (n=3-4 donors) were stimulated with TNFα for 24hr and 48hr. Cell surface B7-H3 was measured by flow cytometry. Results are presented as average fold increase in MFI of each molecule relative to untreated cells. ****p < 0.0001 vs untreated.

EC upregulated other costimulatory molecules, including *ICOSLG*, *TNFRSF14* (HVEM), *CD83*, *CD40* and *TNFRSF9* (4-1BB) transcripts in response to TNFα (**Figure 6A**). *CD40* was increased by 2.93 ± 1.2-fold at 4hr across 6 different primary EC (**Figures 1B** and **S6A**), and protein was detected by 18hr (**Supplemental Figures S6B, S6C**). *TNFRSF9* (4-1BB) mRNA appeared by 4hr (**Figures 6A, B** GSE27870) and was highly increased at 24hr (**Figures 6A, C** GSE144810). *TNFRSF9* mRNA was induced 21.75 ± 7.4-fold (**Figure 1B** and **S6F**). Cell surface expression of 4-1BB (*TNFRSF9*) was detected by 18hr on 6 different primary endothelial cells (**Figure 6D**, **Supplemental Figures S6G, S6H**) and persistent through 48hr by flow cytometry (**Figure 6E**). *ICOSLG* transcript was significantly increased as early as 2hr (**Figures 6A, F** GSE27870) and the elevation in mRNA persisted through 24hr (**Figures 6A, G** GSE144810). Increased *ICOSLG* transcript (12.68 ± 2.6-fold, **Figure 1B** and **Supplemental Figure S6D**) and ICOSL protein cell surface expression was confirmed on HAEC (n=3) and 6 different endothelial cell types (**Figures 6H, I** and **S6G, S6E**) which peaked around 24hr (**Figure 6I**).

Although there was a significant increase in *TNFSF4* (OX40L) transcript in response to TNFα (**Figures 6A, J, K**), the change was nominal and did not result in notable increases in protein expression in HAEC (**Figure 6** **L**, left panel) or in EC from diverse vascular beds (**Figure 6K**, right panel; **S6I-K**). TNFα also modestly increased expression of *TNFRSF14* (HVEM) (**Figures 6M, N**) and *CD83* (**Figures 6O, P** and **S6O**), with the greatest expression at 18hr and 24hr. Interestingly, despite no significant change in mRNA (*CD276*) (**Figures 6A, Q**, **S6L**), cell surface B7-H3 protein was reproducibly down-regulated after stimulation with TNFα (by 20-30%) in primary endothelium (**Figures 6R, S** and **S6M, S6N**).

Endothelial cells did not express *CD80* or *CD86*, two B7 ligand costimulatory molecules that are critical for activation of naïve T cells, in response to TNFα or IFNγ (**Supplemental Figures S2A-E**). In addition, *CD58* (LFA-3), which is known to be constitutively expressed on endothelium (3, 23), was similarly unchanged by TNFα or IFNγ treatment.

Taken together, endothelium responds to TNFα with augmented expression of numerous cytokines, including IL-6, IL-15 and BAFF, and costimulatory molecules, PD-L2, CD40, ICOSL, and 4-1BB, after TNFα stimulation, in addition to HLA class I molecules. Therefore endothelial cells express a constellation of molecules that can influence activation and differentiation of infiltrating leukocytes.

### Time Course of IFNγ-Induced Cytokines Antigen Presentation Machinery and Costimulatory Molecules

**Figure 7:**
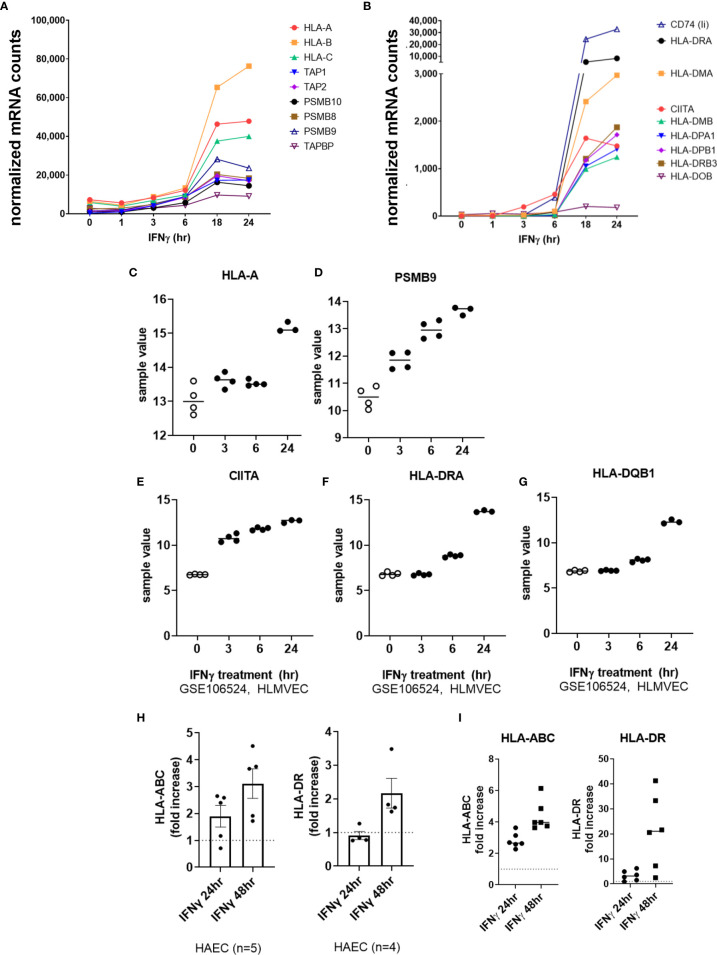
IFNγ induces late phase endothelial expression of HLA, immunoproteasome genes, and cytokines. **(A, B)** HMEC-1 endothelial cells were stimulated with TNFα (20ng/mL) for 1hr, 3hr, 6hr, 18hr or 24hr. Time course of IFNγ induced transcripts in HMEC-1, for HLA class I **(A)**, and **(B)** HLA class II genes. One representative experiment is shown. Results are presented as absolute number of normalized mRNA counts for each gene, normalized to housekeeping controls. **(C-G)** Expression values of *HLA-A* **(C)**, *PSMB9* **(D)**, *CIITA* **(E)**, *HLA-DRA* **(F)** and *HLA-DQB1* **(G)** from GSE106524 are plotted. Human lung endothelial cells were stimulated with IFNγ for 3hr, 6hr or 24hr (n=3-4). **(H)** Primary human aortic endothelial cells (n=4-5 donors) were treated with IFNγ for 24hr or 48hr. Cell surface expression of HLA-ABC and HLA-DR was measured by flow cytometry (n=2). Results are presented as average fold increase in MFI of each molecule relative to untreated cells. **(I)** Primary human (HAEC, HCAEC, HCMVEC, HPAEC, HPMVEC, HLSEC) endothelial cells were treated with IFNγ for 24hr or 48hr. Cell surface expression of HLA-ABC (left panel) and HLA-DR (right panel) was measured by flow cytometry (n=6).

We next tested the effects of type II interferon on endothelial cell costimulatory phenotype. Predictably, HLA class I transcripts (*HLA-A, HLA-B, HLA-C*), immunoproteasome subunits (*PSMB8*, *PSMB9*, *PSMB10*) and class I accessory molecules (*TAP1*, *TAP2*, *TABPBP*) were strongly upregulated by IFNγ stimulation in HMEC-1 and primary endothelial cells (**Figures 1B**, **7A, C, D** and **Supplemental Figure S7**). A change in HLA transcript levels were not seen until 18hr. Nonclassical *HLA-E* and *HLA-F* genes were also increased by IFNγ (*HLA-E*: 4.06 ± 0.3-fold; *HLA-F*: 26.13 ± 5.4-fold at 24hr, n=4 primary EC).

IFNγ-induced class II accessory gene *CIITA* and *CD74* (invariant chain) transcripts appeared as early as 3-6hr, which preceded HLA class II expression (*HLA-DRA*, *DPA*, *DPB*, *HLA-DO*, *HLA-DM*) at 18-24hr (**Figures 7B, E–G** and **Supplemental Figure S7**). Enhanced HLA class I protein expression on primary endothelial cells was seen as early as 18hr, and continued to increase through 48hr of continuous IFNγ exposure (aortic EC: **Figure 7H** **;** 6 primary vascular beds: **Figure 7I**). HLA-DR protein expression was comparatively delayed, with low and inconsistent upregulation at 18-24hr and highest cell surface expression at 48hr (aortic EC: **Figure 7H** **;** 6 primary vascular beds: **Figure 7I**).

**Figure 8:**
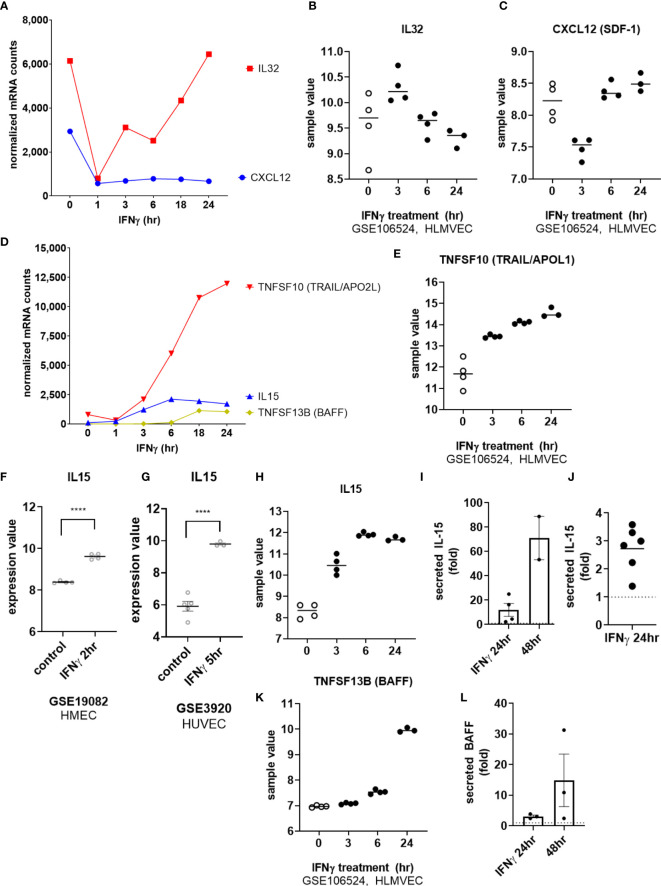
IFNγ alters endothelial cytokine expression patterns. **(A)** HMEC-1 endothelial cells were stimulated with IFNγ (200U/mL) for 1hr, 3hr, 6hr, 18hr or 24hr. Time course of IFNγ suppressed *IL32* and *CXCL12* cytokine transcripts in HMEC-1. One representative experiment is shown. Results are presented as absolute number of normalized mRNA counts for each gene, normalized to housekeeping controls. **(B, C)** Expression values of *IL32* and *CXCL12* from GSE106524 are plotted. Human lung endothelial cells were stimulated with IFNγ for 3hr, 6hr or 24hr (n=3-4). **(D)** HMEC-1 endothelial cells were stimulated with IFNγ (200U/mL) for 1hr, 3hr, 6hr, 18hr or 24hr. Time course of IFNγ induced *TNFSF10*, *IL15* and *TNFSF13B* cytokine transcripts in HMEC-1. One representative experiment is shown. Results are presented as absolute number of normalized mRNA counts for each gene, normalized to housekeeping controls. **(E)** Expression values of *TNFSF10* from GSE106524 are plotted. Human lung endothelial cells were stimulated with IFNγ for 3hr, 6hr or 24hr (n=3-4). **(F, G)** Expression values of *IL15* from GSE3920 are plotted, comparing HMEC (n=3) left untreated or stimulated with IFNγ for 2hr (GSE19082, 8f) or HUVEC (n=3) 5hr (GSE3920, 8g). Value distributions were compared by unpaired t test. Results are presented with each measured value, and the line at the median. ****p < 0.0001 compared to control. **(H)** Expression values of *IL15* from GSE106524 are plotted. Human lung endothelial cells were stimulated with IFNγ for 3hr, 6hr or 24hr (n=3-4). **(I, J)**. Primary human aortic endothelial cells (n=2) **(I)** and HCAEC, HCMVEC, HPAEC, HPMVEC, HLSEC **(J)** were treated with IFNγ for 24hr or 48hr. Conditioned medium was assayed for secreted IL-15 protein by ELISA. Results are presented as fold increase in the concentration of secreted cytokine compared to untreated controls at the same time point. **(K)** Expression values of *TNFSF13B* from GSE106524 are plotted. Human lung endothelial cells were stimulated with IFNγ for 3hr, 6hr or 24hr (n=3-4). **(L)** Primary human aortic endothelial cells were treated with IFNγ for 24hr or 48hr. Conditioned medium was assayed for secreted BAFF protein by ELISA. Results are presented as fold increase in the concentration of secreted cytokine compared to untreated controls at the same time point (n=3).

Among cytokines, IFNγ exposure had no effect on endothelial expression of *IL1B*, *IL6*, *TNFSF15* (TL1A/VEGI) or *LIF*. As with TNFα, *IL32* mRNA was rapidly downregulated in IFNγ-activated endothelial cells (at 1hr), and recovered to baseline levels at 18hr (**Figures 8A, B**). Constitutive *CXCL12* (SDF-1) mRNA was also substantially decreased by IFNγ and remained suppressed through 24hr of IFNγ exposure (**Figures 8A, C**). On the other hand, endothelial cells stimulated with IFNγ upregulated *IL15* and *TNFSF10* (TRAIL) by 6hr and *TNFSF13B* (BAFF/BLyS) by 18hr (**Figures 8D, E**).

We confirmed mRNA induction by IFNγ of *IL15* (12.74 ± 5.4-fold at 24hr) and *TNFSF13B* (BAFF/BLyS) (19.41 ± 5.8-fold) in 4 different primary endothelial cell beds (**Figures 1B** and **Supplemental Figures S8A, S8D**), and corroborated these observations in publicly available datasets of IFNγ-stimulated EC (**Figure 8F**, GSE19082; **Figure 8G**, GSE3920, and **Figures 8H, K**, GSE106524). Inducible protein secretion from IFNγ-stimulated primary aortic EC was detected as early as 24hr and was significantly elevated at 48hr for IL-15 (2.48 ± 0.04-fold at 48hr, **Figures 8I, 8J** and **S8B, S8C**) and BAFF (3.83 ± 0.78-fold at 48hr, **Figures 8L** and **S8E**).

**Figure 9:**
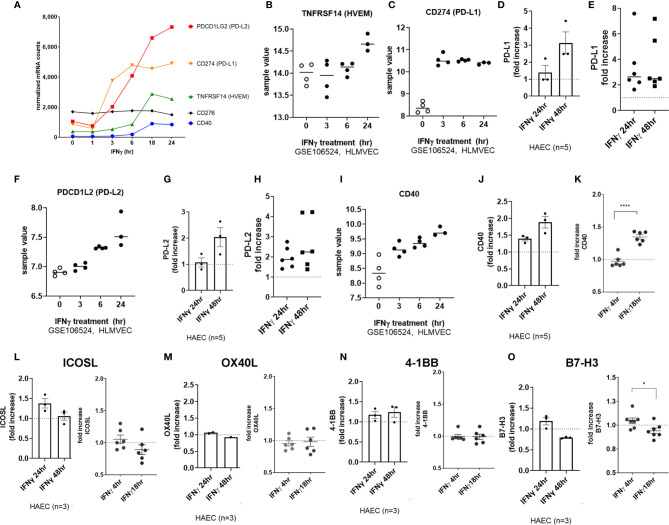
IFNγ induces endothelial expression of coinhibitory and costimulatory molecules. **(A)** HMEC-1 endothelial cells were stimulated with IFNγ (200U/mL) for 1hr, 3hr, 6hr, 18hr or 24hr. Time course of IFNγ induced transcripts in HMEC-1, for *PDCD1LG2, CD274, TNFRSF14*, *CD40*, and *CD276* genes. One representative experiment is shown. Results are presented as absolute number of normalized mRNA counts for each gene, normalized to housekeeping controls. **(B, C)** Expression values of *TNFRSF14* **(B)** and *CD274* **(C)** from GSE106524 are plotted. Human lung endothelial cells were stimulated with IFNγ for 3hr, 6hr or 24hr (n=3-4). **(D)** Primary human aortic endothelial cells (n=3 donors) were treated with IFNγ for 24hr. Cell surface expression of PD-L1 was measured by flow cytometry (n=3). Results are presented as average fold increase in MFI of each molecule relative to untreated cells. **(E)** Primary human (HAEC, HCAEC, HCMVEC, HPAEC, HPMVEC, HLSEC) endothelial cells were treated with IFNγ for 24hr or 48hr. Cell surface expression of PD-L1 was measured by flow cytometry (n=6). **(F)** Expression values of *PDCD1L2* from GSE106524 are plotted. Human lung endothelial cells were stimulated with IFNγ for 3hr, 6hr or 24hr (n=3-4). **(G)** Primary human aortic endothelial cells (n=3 donors) were treated with IFNγ for 24hr. Cell surface expression of PD-L2 was measured by flow cytometry. Results are presented as average fold increase in MFI of each molecule relative to untreated cells. **(H)** Primary human (HCAEC, HCMVEC, HPAEC, HPMVEC, HRGEC, HLSEC) endothelial cells were treated with IFNγ for 24hr or 48hr. Cell surface expression of PD-L2 was measured by flow cytometry (n=6). **(I)** Expression values of *CD40* from GSE106524 are plotted. Human lung endothelial cells were stimulated with IFNγ for 3hr, 6hr or 24hr (n=3-4). **(J)** Primary human aortic endothelial cells (n=3 donors) were treated with IFNγ for 24hr. Cell surface expression of CD40 was measured by flow cytometry. Results are presented as average fold increase in MFI of each molecule relative to untreated cells. **(K)** Primary human (HCAEC, HCMVEC, HPAEC, HPMVEC, HRGEC, HLSEC) endothelial cells were treated with IFNγ for 24hr or 48hr. Cell surface expression of CD40 was measured by flow cytometry (n=6). **(L-O)** Primary human (HAEC [n=3], HCAEC, HCMVEC, HPAEC, HPMVEC, HRGEC and HLSEC) endothelial cells were treated with IFNγ for 4hr, 18hr, 24hr or 48hr. Cell surface expression of **(L)** ICOSL, **(M)** OX40L, **(N)** 4-1BB and **(O)** B7-H3 was measured by flow cytometry. *p < 0.05, ****p < 0.0001 by paired t test.

Among costimulatory molecules, IFNγ promoted upregulation of *CD274* (PD-L1) and *PDCD1LG2* (PD-L2) transcripts by 3hr, with later and more modest *CD40* and *TNFRSF14* (HVEM) (**Figure 9A**). We confirmed in 4 other primary endothelial cell vascular beds, that *CD40* (3.71 ± 1.4-fold), *PDCD1LG2* (PD-L2) (2.71 ± 0.39-fold), and *CD274* (PD-L1) (5.64 ± 2.6-fold) transcripts were upregulated by IFNγ at 24hr (**Figures 1B, 9B, F, I** and **Supplemental Figures S9A, B**).

PD-L1 and PD-L2 protein expression was significantly increased at the aortic endothelial cell surface by 24hr (**Figures 9D, G** **;** raw MFI in **Supplemental Figures S9C, F, G**). Similar increases at the protein level were seen using 6 primary endothelial cells from different vascular beds (**Figure 9E**, PD-L1: 3.40 ± 2.1-fold at 24hr, 3.64 ± 2.2-fold at 48hr; **Figure 8H**, PD-L2: 1.97 ± 0.5-fold at 24hr, 2.65 ± 1.2-fold at 48hr, n=6; raw MFI in **Supplemental Figures S9B, E, G**). Cell surface CD40 was also enhanced on aortic endothelial cells (1.94 ± 0.46-fold at 24hr, 2.51 ± 0.4-fold at 48hr, **Figure 9J** and **S10C**) and on 6 other primary endothelial cell types as early as 18hr (**Figures 9K**, **S10B**).

In contrast, there was no significant increase in *CD83*, *TNFSF4* (OX40L), *TNFRSF9* (4-1BB), *ICOSLG* or *CD276* (B7-H3) protein or mRNA after IFNγ stimulation on HMEC-1 or any primary endothelial cells (**Figures 9A, L-O** and **Supplemental Figure S10**).

Collectively, these results demonstrate that IFNγ activation of endothelial cells results in late display of a restricted set of cytokines, IL-15 and BAFF, and costimulatory and coinhibitory molecules CD40, PD-L1 and PD-L2, in addition to extensive HLA class I and HLA class II antigen presentation machinery.

### Short Exposure to TNFα Enhances Late Phase HLA Class I Expression by Endothelial Cells, But Not Cytokine or Costimulatory Molecule Expression

After defining the pattern of cytokines and costimulatory molecules expressed by cytokine-activated endothelial cells, we next assessed the timing of endothelial return to quiescence. After 3hr of TNFα cytokine priming, endothelial cells were withdrawn from cytokine for an additional 3-45hr. Expression of costimulatory factors was measured, compared with the same times in the continuous presence of cytokine.

**Figure 10:**
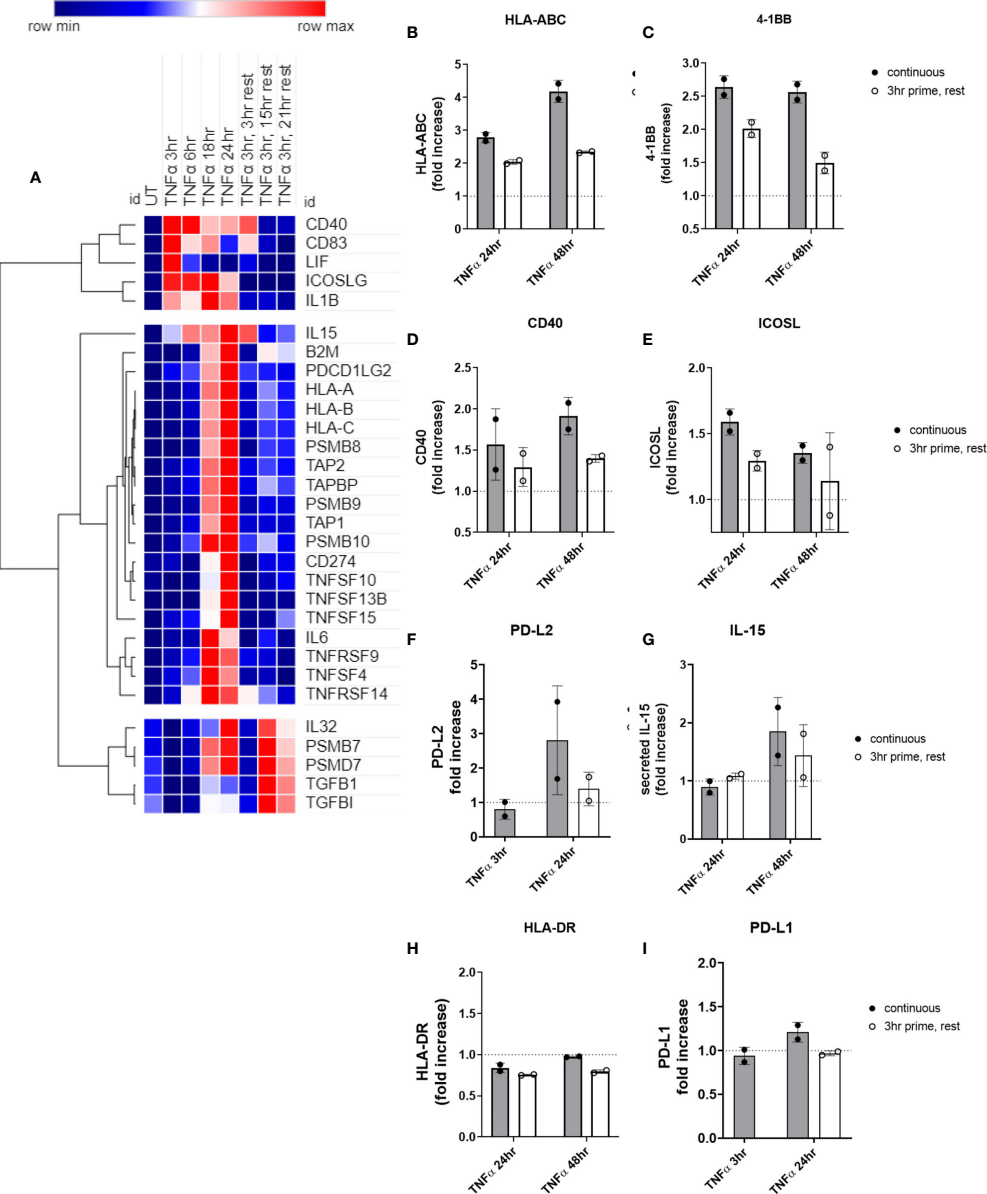
Short priming with TNFα followed by withdrawal/rest in the absence of cytokine. **(A)** HMEC-1 endothelial cells were treated with TNFα (20ng/mL) for 3hr, 6hr, 18hr, or 24hr. In some conditions, medium was replaced after 3hr with medium without TNFα, and cells were allowed to rest for an additional 3hr, 15hr or 21hr. mRNA counts were measured by Nanostring and normalized to housekeeping genes and controls. Heat map shows relative gene expression over time, with normalization across genes/rows. Hierarchical clustering of rows by one minus Pearson correlation. **(B-I)** Primary human aortic endothelial cells were treated with TNFα (20ng/mL) for 3hr, 24hr or 48hr; or primed with TNFα for 3hr followed by an additional 21hr or 45hr without cytokine. Cell surface expression of **(B)** HLA-ABC, **(C)** 4-1BB, **(D)** ICOSL, **(E)** CD40, **(F)** PD-L2, **(H)** HLA-DR, and **(I)** PD-L1 was measured by flow cytometry (n=2). Secreted IL-15 was measured in the supernatants by ELISA **(G)**. Quantitated results are presented as average fold increase in MFI or cytokine of each molecule relative to untreated cells. Filled bars show values for the continuous presence of TNFα; open bars show results at the end of the rest period after 3hr priming.

We observed three distinct patterns after TNFα removal: 1) transcripts recovered to baseline after TNFα withdrawal (*TNFSF13B*, *IL6*; *CD274*, *TNFRSF14*, *TNFRSF9*, *CD83*, *ICOSLG*, *CD40*); 2) TNFα prime/withdrawal yielded intermediate induction, elevated above baseline but lower than continuous (*TNFSF15*, *IL15*, *TNFSF10*; HLA class I, proteasome components); or 3) TNFα prime/withdrawal resulted in comparable kinetics and magnitude of expression to continuous stimulation (*PDGFB*, *TGFBI*, *LIF*, *IL32*; *PDCD1LG2*) (**Figure 10A**). For example, after only 3hr priming with TNFα, HLA class I, TAP and immunoproteasome mRNA expression showed intermediate levels that were lower than with continuous cytokine but higher than baseline. Discrete mRNA counts for genes of interest over the time course are provided in **Supplementary Figures S11A–D**, **S12A–K**, and **S13A–G**.

We next measured residual protein expression in endothelial cells after 3hr TNFα priming. Cell surface HLA I (**Figure 10B**) and 4-1BB (**Figure 10C**), remained elevated above baseline as long as 48hr later, albeit significantly lower compared with chronic TNFα stimulation. However, cell surface ICOSL (**Figure 10D**), CD40 (**Figure 10E**), and PD-L2 (**Figure 10F**), and secreted IL-15 (**Figure 10G**) all declined to untreated levels after TNFα was withdrawn. As with chronic stimulation, no changes in HLA-DR or PD-L1 were seen (**Figures 10H, I**). Representative histograms are shown in **Supplemental Figure S14**.

### Brief Priming With IFNγ Is Sufficient to Trigger Long-Term Augmentation of HLA Class I, HLA Class II, Cytokines and Costimulatory Molecules

**Figure 11:**
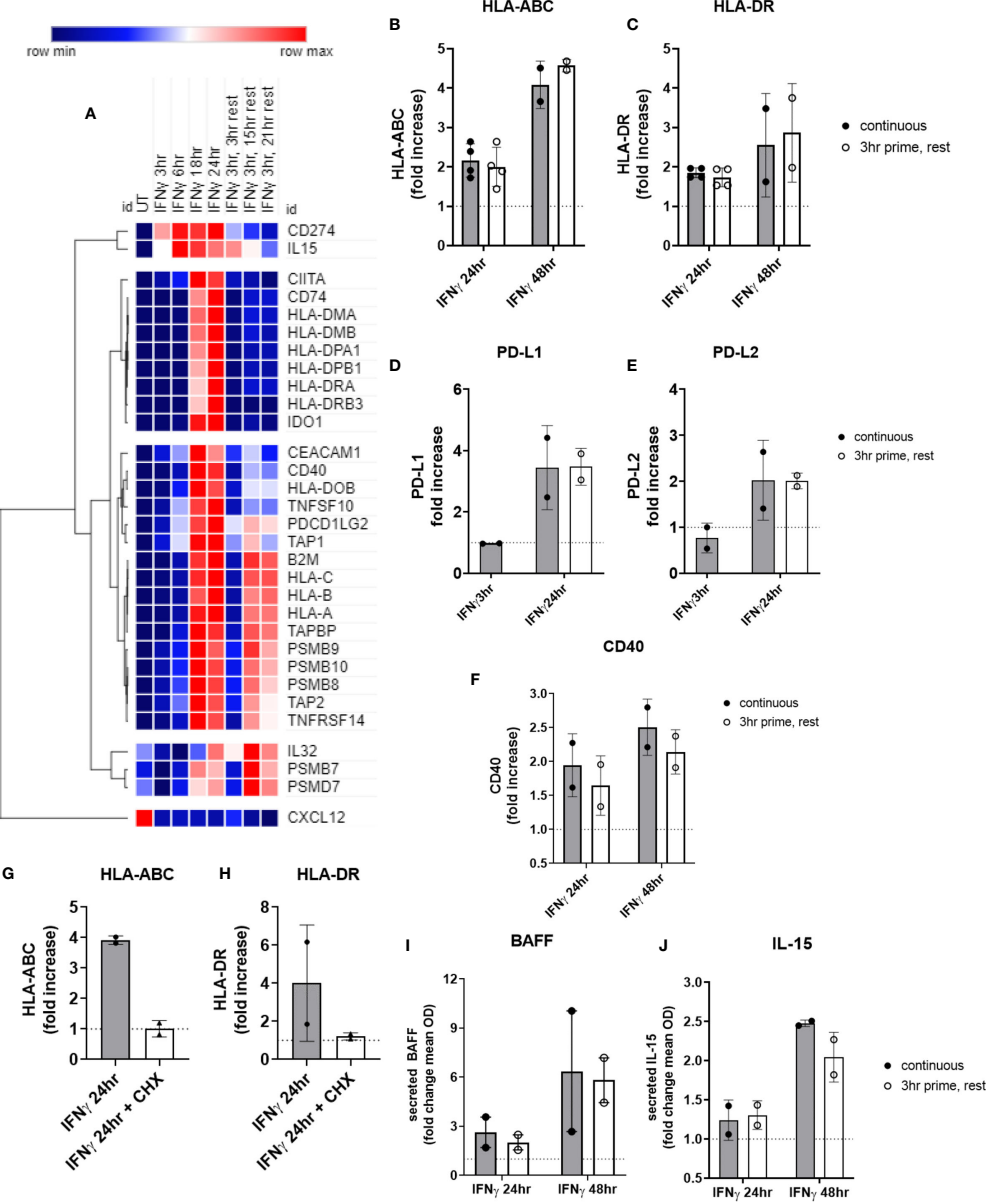
Short priming with IFNγ followed by withdrawal/rest in the absence of cytokine. **(A)** HMEC-1 endothelial cells were treated with IFNγ (200U/mL) for 3hr, 6hr, 18hr, or 24hr. In some conditions, medium was replaced after 3hr with medium without IFNγ, and cells were allowed to rest for an additional 3hr, 15hr or 21hr. mRNA counts were measured by Nanostring and normalized to housekeeping genes and controls. Heat map shows relative gene expression over time, with normalization across genes/rows. Hierarchical clustering of rows by one minus pearson correlation. **(B–F)** Primary human aortic endothelial cells (n=2 donors) were treated with IFNγ for 24hr or 48hr; or primed with IFNγ for 3hr followed by an additional 21hr or 45hr without cytokine. Cell surface expression of **(B)** HLA-ABC, **(C)** HLA-DR, **(D)** PD-L1, **(E)** PD-L2 and **(F)** CD40 was measured by flow cytometry (n=2). Quantitated results are presented as average fold increase in MFI of each molecule relative to untreated cells **(C)**. *(G, H) Primary human aortic endothelial cells (n=2 donors) were treated with IFNγ for 24hr (black bars); or pulsed with IFNγ for 3hr followed by an additional 21hr in plain medium (grey bars) or medium with cycloheximide (CHX, white bars). Cell surface expression of **(G)** HLA-ABC and **(H)** HLA-DR was measured by flow cytometry. **(I, J)** Primary human aortic endothelial cells were treated with IFNγ for 24hr or 48hr (black bars); or primed with IFNγ for 3hr followed by an additional 21hr or 45hr without cytokine (white bars). Conditioned medium was assayed for secreted **(I)** BAFF or **(J)** IL-15 protein by ELISA. Results are expressed as average fold increase in concentration in the supernatant +/-SEM (n=2).

We performed the same experiments testing priming of endothelial cells with IFNγ for 3hr, followed by removal. Surprisingly, nearly all IFNγ-induced costimulatory molecules and cytokines persisted long after IFNγ withdrawal. Two patterns of transcript expression after IFNγ withdrawal were observed: 1) IFNγ prime/withdrawal caused intermediate gene expression, above untreated but lower than continuous (*CD274*, *CD40*; *TNFSF13B*, *TNFSF10*; HLA class II, *CD74*, *CIITA*; *IDO1*); or 2) IFNγ prime/withdrawal resulted in equivalent potentiation as chronic stimulation (*IL32*, *IL15*, *CXCL12*; *TNFRSF14*, *PDCD1LG2*; HLA class I, *TAP1*, *TAP2*, *TAPBP*, *PSMB8*, *PSMB9*, *PSMB10*) (**Figure 11A**). Only suppression of constitutive *CXCL12* and *IL32* was rapidly reversed when IFNγ was removed (**Figure 11A**). Normalized mRNA counts for genes of interest over time are shown in **Supplemental Figures S15** **and** **S16**.

Most strikingly, late phase HLA class I and proteasome induction were equal whether IFNγ was continuously present or had been removed after 3hr (**Figure 11A**). At the protein level, cell surface HLA class I and class II expression were equivalent to continuous IFNγ stimulation, up to 45hr later (**Figures 11B, C**). Similarly, short IFNγ priming triggered delayed expression of PD-L1 (**Figure 11D**) and PD-L2 (**Figure 11E**) that was comparable to chronic IFNγ presence (PD-L1: continuous 2.48-fold vs. rest 3.05-fold; PD-L2: continuous 1.41-fold vs. rest 1.89-fold). Only CD40 (**Figure 11F**) was slightly reduced at 48hr after IFNγ pulse compared to persistent IFNγ stimulation.

When EC were primed with IFNγ for 3hr, then medium was replaced with cycloheximide without IFNγ for an additional 21hr, cell surface expression of HLA-ABC and HLA-DR was abolished (**Figures 11G, H**), suggesting that new transcriptional events after 3hr are required for protracted HLA expression by IFNγ.

Lastly, secreted BAFF protein was increased 2.64 ± 1.3-fold at 24hr, and 6.37 ± 5.2-fold at 48hr of IFNγ stimulation. Although no cytokine secretion was detected at the 3hr time point, there was no significant difference in the amount of BAFF secreted by EC 21hr and 45hr later when they had only been exposed to IFNγ for 3hr (2.02 ± 0.65-fold at 24hr, 5.82 ± 1.9-fold at 48hr) (**Figure 11I**). Similarly, IL-15 was elaborated up to 45hr later from endothelial cells pulsed for only 3hr with IFNγ, with no significant difference compared to chronic IFNγ presence (2.04 ± 0.2-fold at 48hr) (**Figures 11J** and **S16K**).

These results demonstrate that brief priming of endothelial cells with type II interferon elicits a protracted costimulatory phenotype with elevated HLA class I and class II, costimulatory molecules and cytokine expression.

## Discussion

In this study, we characterized inducible endothelial expression of the three major signals required by T cells for activation: antigen presentation, cytokines and costimulatory molecules. We also investigated whether an altered endothelial cell phenotype was durable, or if it rapidly resolved in the absence of inflammatory stimulus. These questions are particularly relevant in the context of solid organ transplantation, where allogeneic T cells encounter HLA mismatched donor vascular endothelial cells and cause rejection of the transplanted tissue. T cell activation and peripheral antigen recognition within self HLA is also important in autoimmunity and atherosclerosis.

Endothelial activation by cytokines canonically leads to increased leukocyte-endothelial interactions due to upregulation of adhesion molecules and chemoattractants. In addition to actively coordinating recruitment of leukocytes, endothelial cells are strategically positioned to shape the immune response relative to local cues at the site of diapedesis. The question of endothelial antigen presenting capacity is particularly relevant in transplantation, where resident vascular cells from the donor express foreign HLA molecules and directly encounter the recipient’s adaptive immune system. In the 1980s *in situ* HLA expression by endothelium in transplanted organs was therefore heavily investigated and confirmed (24–28). This was followed by seminal *in vitro* work from the Pober and Lechler labs showing that coculture of bulk allogeneic T cells with endothelium promoted T cell proliferation and acquisition of activation markers (23, 29–31), in a contact-dependent manner (3) and in the absence of exogenous mitogens (32). The effect was observed with both CD4+ and CD8+ T cells (2, 33). Later studies resolved that endothelial cells could not prime naïve T cells and indeed may even elicit nonresponsiveness in the CD45RA+ population (6, 34–36). Instead, memory T cells were specifically activated by allogeneic endothelium (37–40). The current paradigm is that endothelial cells are able to promote proliferation and activation at least of memory T cells, with further specific effects on differentiation and skewing still being revealed (particularly of Treg and Th17) (40).

In this study, we provide a broader classification of endothelial costimulatory molecule patterns under cytokine stimulation. For example, we report inducible endothelial expression of cytokines that are less well-described in the vascular compartment; some of which have been shown only a few times on EC and with unclear functional relevance.

Endothelial cells express high levels of HLA class I molecules. *In situ*, microvascular endothelial cells may also express HLA class II, although it is typically lost in cell culture. Like many other cells, can upregulate HLA class II molecules in response to type II interferon, as well as further enhance cell surface HLA class I in response to TNFα or IFNγ. *HLA-DQB1* expression by endothelial cells is of high interest particularly in solid organ transplantation, where it is the most common target of donor specific alloantibodies associated with poor long-term graft outcomes. Moreover, polymorphisms in the *HLA-DRB1* and *DQB1* genes are strongly associated with and often causative in numerous autoimmune diseases. MHC accessory molecules critical for antigen processing and presentation are also upregulated by inflammatory cytokines in the vascular compartment. For example, we found low basal expression of *TAP1* and *TAP2*, transporters essential for import of cytosolic peptide antigens into the endoplasmic reticulum; but these were substantially upregulated by TNFα and IFNγ. Moreover, components of the immunoproteasome (*PSMB8, PSMB9*, *PSMB10*) essential for optimal proteolytic antigen processing for binding in the MHC class I groove (41), were specifically upregulated, while genes encoding the constitutive proteasome (*PSMB7*, *PSMD7*) were highly expressed and either unaffected or downregulated by cytokine treatment. Under IFNγ stimulation, HLA class II-associated antigen presentation machinery were increased, including the master transcription factor *CIITA*, the invariant chain *CD74*, and the peptide chaperones *HLA-DM* and *HLA-DO*. Collectively, these changes point to a dramatic physiological shift in the function of endothelium towards antigen presentation under stimulation with Th1 proinflammatory cytokines.

Which contact-dependent molecules are involved in the endothelial activating effect on T cells? Endothelial cells mostly lack expression of the B7 family CD28 ligands CD80 and CD86 (29, 42), under either resting and stimulated conditions, which may account for the inability to stimulate naïve T cells that are heavily dependent on this signal. Yet, even this is controversial, with some early studies finding endothelial expression of CD86 (43, 44); and more recently showing CD86 in specialized vascular compartments (45). In our results, no CD80 or CD86 expression was detected in cultured human endothelial cells, either resting or activated by TNFα or IFNγ.

Initially endothelial ICAM-1 and LFA-3 were identified as important for the proliferative effect on T cells (30, 46). LFA-3 (*CD58*) is constitutively expressed by endothelium (47), acting as a ligand for CD2 in costimulation for CD28- T cells (48) and mediating endothelial-T cell activation (23). While we did observe LFA-3 expression across endothelia, neither TNFα nor IFNγ increased its expression. Therefore LFA-3 may function in basal T cell-endothelial cell interactions but not in inflammatory T cell activation.

Endothelial expression of CD40 is well-described (49). Engagement of endothelial CD40 by CD40L on activated T cells elicits a potent proinflammatory stress response in the endothelium (50, 51). As others have reported (52, 53), we found that both TNFα and IFNγ modestly increased expression of CD40 transcript and protein on endothelial cells, where induction was greater with TNFα than IFNγ. Thus, in addition to signaling to the T cell, increased expression of CD40 may further exacerbate endothelial cells to an inflammatory activation state.

*CD83* was transiently increased in endothelial cells by TNFα but not IFNγ, with relatively low but detectable mRNA counts. Interestingly, CD83 is a marker of mature dendritic cells, which appears to have a negative regulatory role on the function of both T and B cells and therefore may be critical in tolerance and resolution of inflammation (54, 55). Other positive immune checkpoint molecules include ICOSL, OX40L, and 4-1BB. 4-1BB expression in the vasculature was a driver of inflammation in a murine model of atherosclerosis (56), and ICOSL expression by endothelium also mediated T cell costimulation (57). In our experiments, the stimulatory ICOSL and 4-1BB were induced on endothelial cells by TNFα but not IFNγ.

Both TNFα and IFNγ augmented endothelial expression of ligands for PD-1, a critical immune inhibitory checkpoint for T cells. Importantly, our data show that TNFα did not induce PD-L1, only PD-L2; while IFNγ highly upregulated both. PD-L2 has a slightly higher affinity for PD-1 compared with the more broadly expressed PD-L1 (58) and had been thought to be restricted to antigen presenting cells (59). However, we and others have observed inducible PD-L2 expression on human endothelium. Functionally, PD-L1 and PD-L2 suppressed syngeneic CD8+ T cell activation by endothelial cells (60, 61).

Finally, among soluble signals produced by endothelial cells, both TNFα and IFNγ suppressed constitutive expression of *CXCL12* (SDF-1) and *IL32*. IL-32 is relatively recently identified, and growing evidence supports its function as a pro-inflammatory cytokine. *IL32* was cytokine-inducible in EC, and also likely has a function in vascular inflammation (62, 63) as well as effects on the adaptive immune compartment, although its functions are still being elucidated. Within the lymph node, *CXCL12* was uniquely expressed by blood but not lymphatic endothelium (1). *CXCL12* (SDF-1) also enhances TCR stimulation as an additional costimulatory signal (64). It will be intriguing to determine the biological consequence of a switch from constitutive endothelial expression of these factors to other cytokines when initiating a pro-inflammatory program.

Other better described inducible cytokines produced by inflamed EC include *IL6*, *LIF*, *TNFSF15* (TL1A) and *TNFSF10* (TRAIL). *TNFSF10* (TRAIL) presentation by APCs alters their immunostimulatory function, inhibiting TCR signaling when TRAIL-R on T cells is engaged (65). TNFα, but not IFNγ, triggered weak endothelial expression of *IL6* and *LIF*. IL-6 skews Th2 development and is also critical for Tfh and B cell antibody responses, while leukemia inhibitory factor (LIF) is an IL-6 family cytokine that plays a central role in T cell lineage development, particularly Treg vs. Th17 divergence (66).

We were surprised to find that activated endothelial cells elaborated BAFF/BLyS and IL-15, which we detected both at the mRNA level and secreted protein in the supernatant. Both TNFα and IFNγ elicited expression of the well-known pro-survival factor for B cells BAFF. *TNFSF13B* (BAFF) secretion has been demonstrated from EC in the bone marrow, as well as in the settings of malignancy, autoimmunity and viral infection (17–19, 67). Stromal endothelial cell-derived BAFF supported survival of leukemic cells in CLL (67), but there are few other reports of endothelium as a significant source of this cytokine. IL-15 was also reportedly produced by EC under the conditions of viral or autoimmune disease (20, 21). It was recently demonstrated that EC transpresent IL-15 to CD8 T cells (22). In addition to effects on B cells, BAFF also stimulates T cells, including potentiation of TCR activation, T cell survival and proliferation (68, 69). BAFF was recently implicated in the formation of tertiary lymphoid structures in the autoimmune disease lupus nephritis (70), and similarly BAFF expression correlated with formation of TLS in giant cell arteritis (71). IL-15 is an important cytokine for NK cells, and drives central memory CD8+ T cells. Therefore, local production of these factors may propel leukocyte activation and possibly influence the formation of TLS within peripheral tissues.

Our results illustrate the temporal phases of endothelial cell phenotype. It is intriguing that that the antigen presentation and costimulatory molecules induced by TNFα and IFNγ appeared in the later stages of activation, whereas many chemokines and adhesion molecules characteristically appear earlier (3-6hr) as part of an acute and transient phase. This is suggestive of a multi-phase response beginning with pro-adhesive recruitment functions and culminating in costimulatory or coinhibitory effects on the leukocyte compartment.

The significance of high endothelial HLA class I expression in the context of certain positive immune checkpoint molecules (i.e. in the context of TNFα) compared with high HLA I and HLA II but fewer available costimulatory molecules (i.e. IFNγ context) is unknown. Moreover, the cumulative biological impact of these combined signals remains to be elucidated. Experiments are ongoing in our lab to elucidate how these cues are functionally integrated to tune activation and skewing of the adaptive immune compartment. Potentially the context (TNFα vs. IFNγ) may drive disparate outcomes of inflammation or tolerance, stimulation or immunoregulation of the adaptive response. The specific functional contribution of each costimulatory molecule and cytokine expressed by endothelial cells on T cell activation and function has also yet to be fleshed out, and work is ongoing to elucidate the functional significance of endothelial costimulatory molecules on T cell activation.

Inappropriate prolongation and failure to initiate healing and return to non-inflamed homeostasis lies at the heart of numerous chronic diseases, including vascular diseases. Resolution requires termination of pro-inflammatory signaling, either through direct requirement for stimulation (i.e. passive, turns off when stimulus is gone) or active negative feedback and desensitization. We present novel data showing that the endothelial cell costimulatory phenotype remains altered for at least two days after last exposure to inflammatory cytokines. TNFα and IFNγ patterns were distinct, where TNFα-inducible changes were mostly self-limiting over time, and the majority of which rapidly declined when TNFα was removed. IFNγ-triggered changes manifested as late phase responses, and intriguingly IFNγ effects on endothelial cells were extended without the requirement for continual stimulation. Unlike TNFα, withdrawal of the pro-inflammatory IFN signal does not alone contribute to resolution; but rather a stable program results in moderately persistent inflammation. We infer therefore that the perturbations caused by IFNγ require more time to contract and restore endothelial cell homeostasis.

Prior studies mostly employed human umbilical vein endothelium (HUVEC), which are widely available and easy to propagate, but differ in important ways from arterial and capillary endothelial cells lining the vasculature of organs and tissues. We provide several lines of evidence by analyzing public datasets and testing multiple different human endothelial cell types, at both the transcript and protein level, that endothelial cells express an array of costimulatory molecules and cytokines. It is possible that there are tissue specific patterns as well, particularly at sites of entry to immune privileged tissue (cornea, brain, testes) and within the liver, which is widely viewed as tolerogenic. For example, liver sinusoidal endothelium may have a limited capacity to stimulate naïve as well as memory T cells, with a more tolerogenic effect compared with other vascular beds (42, 72–74). The results presented herein do support that costimulatory molecules are broadly induced on multiple endothelial cell types. Our initial study was not designed to address site-specific differences in endothelial heterogeneity of costimulatory molecules, but this will be an important outgrowth to pursue.

By technical necessity most EC-T cell costimulation studies have employed an allogeneic system, in which endothelial cells express mismatched MHC molecules that can activate T cells through direct allorecognition. In an indirect allorecognition model *in vivo*, it was shown that EC can effectively present alloantigen to MHC matched T cells (75). It will be important for future work to model both direct allorecognition with genetically mismatched cells, such as in the context of transplantation, as well as HLA syngeneic antigen presentation by endothelial cells, as in atherosclerosis, malignancy and infection.

## Conclusions and Implications

Although endothelial cells lacked CD28 ligands (CD80, CD86) and did not produce many of the typical cytokines needed for Th skewing (IFNs, IL-12, IL-21, IL-4), they could be provoked to express PD-1 ligands, CD40 and other cytokines that can bias T cell activation. That systemic and tissue resident vascular endothelial cells possess a wide constellation of costimulatory molecules suggests that adaptive immunity takes shape not only in secondary lymphoid organs but also locally in the periphery. The role of the local vasculature in initiating or propelling activation of the adaptive immune compartment will need to be a consideration in therapeutic approaches employing costimulatory agonism or blockade, such as cancer immunotherapy, autoimmunity and organ transplant immunosuppression.

## Data Availability Statement

The raw data supporting the conclusions of this article will be made available by the authors, without undue reservation.

## Ethics Statement

All experiments using human endothelial cells were approved by the UCLA Institutional Review Board (IRB#17-000477). Written informed consent for participation was not required for this study in accordance with the national legislation and the institutional requirements.

## Author Contributions

NV is responsible for experiment conception and design, data collection and analysis, and manuscript preparation. The author contributed to the article and approved the submitted version.

## Funding

Support for this was provided in part by the Norman E. Shumway Career Development Award from the International Society of Heart and Lung Transplantation and Enduring Hearts (to NV).

## Conflict of Interest

The author declares that the research was conducted in the absence of any commercial or financial relationships that could be construed as a potential conflict of interest.

## Abbreviations

EC, endothelial cell; IFNγ, interferon gamma; HAEC, human aortic endothelial cell; HCAEC, human coronary artery endothelial cell; HCMVEC, human cardiac microvascular endothelial cell; HPAEC, human pulmonary artery endothelial cell; HLMVEC, human lung microvascular endothelial cell; HDMVEC, human dermal microvascular endothelial cell; HRGEC, human renal glomerular endothelial cell; HLSEC, human liver sinusoidal endothelial cell; TNFα, tumor necrosis factor alpha
