# Supplementary material for: IFNγ, and to a Lesser Extent TNFα, Provokes a Sustained Endothelial Costimulatory Phenotype
Source: Front Immunol. 2021 Apr 15;12:648946. doi: 10.3389/fimmu.2021.648946 (PMC8082142; doi:10.3389/fimmu.2021.648946)
Supplement: Supplementary file 1 [file DataSheet_1.docx]

**Supplemental Tables**

| **Reagent** | **Vendor** | **Catalog** |
| --- | --- | --- |
| TNF alpha (recombinant, Humankine) | Sigma | H8916-10UG |
| human IFNγ | R&D Systems | 285IF-100/CF |
| Buffer RLT | Qiagen | 79216 |
| CD40-Alexa Fluor 488 | Biolegend | 334318 |
| OX40L-PE | Biolegend | 326307 |
| B7-H3-PerCP/Cy5.5 | Biolegend | 351009 |
| HLA-ABC-Brilliant Violet 510 | Biolegend | 311436 |
| HLA-DR-Brilliant Violet 421 | Biolegend | 307636 |
| ICOSL-PE/Cy7 | Biolegend | 309409 |
| 4-1BB-APC | Biolegend | 309809 |
| PD-L2-APC | Biolegend | 345507 |
| PD-L1-FITC | Biolegend | 374509 |
| Human BAFF Quantikine ELISA | R&D Systems | DBLYS0B |
| Human IL-15 Quantikine ELISA | R&D Systems | D1500 |
| Milliplex MAP Human Cytokine/Chemokine 38-Plex panel | Millipore Sigma | HCYTMAG-60K-PX38 |
| Trypsin EDTA | Sigma | T4049-100mL |
| Accutase | Sigma | A6964-500ML |
| Endothelial Cell Growth Medium MV | PromoCell | C-22020 |
| M199 | ThermoFisher | 11150059 |
| gelatin type B solution | Sigma | G1393-100ML |

Supplemental Table 1. List of reagents and sources used in this study.

| **Cell** | **Vascular Bed** | **Vendor** | **Catalog #** | **Vendor** | **Catalog #** |
| --- | --- | --- | --- | --- | --- |
| HMEC-1 | neonatal foreskin, immortalized | ATCC | CRL-3243 |  |  |
| HAEC | aorta, primary | Lonza | CC-2535 | PromoCell | C-12271 |
| HCAEC | coronary artery, primary | ATCC | PCS-100-020 | PromoCell | C-12221 |
| HCMVEC | cardiac microvascular, primary | Lonza | CC-7030 | PromoCell | C-12285 |
| HPAEC | pulmonary artery, primary | ATCC | PCS-100-022 | PromoCell | C-12241 |
| HLMVEC | lung microvascular, primary | Lonza | CC-2527 | PromoCell | C-12282 |
| HRGEC | renal glomerular, primary | ScienCell | 4000 | Cell Systems | ACBRI128 |
| HDMVEC | dermal microvascular, primary | Lonza | CC-2543 | PromoCell | C-12212 |

Supplemental Table 2. List of endothelial cells and their sources used in this study.

| **Target** | **Fluorophore** | **Vendor** | **Catalog** | **Dilution** |
| --- | --- | --- | --- | --- |
| **Panel 1** |  |  |  |  |
| CD40 | Alexa Fluor 488 | Biolegend | 334318 | 5:100 |
| OX40L | PE | Biolegend | 326307 | 5:100 |
| B7-H3 | PerCP/Cy5.5 | Biolegend | 351009 | 5:100 |
| HLA-ABC | Brilliant Violet 510 | Biolegend | 311436 | 2.5:100 |
| HLA-DR | Brilliant Violet 421 | Biolegend | 307636 | 5:100 |
| ICOSL | PE/Cy7 | Biolegend | 309409 | 5:100 |
| 4-1BB | APC | Biolegend | 309809 | 5:100 |
| **Panel 2** |  |  |  |  |
| PD-L1-FITC | FITC | Biolegend | 374509 | 5:100 |
| PD-L2-APC | APC | Biolegend | 345507 | 5:100 |
| HLA-ABC | Brilliant Violet 510 | Biolegend | 311436 | 2.5:100 |
| HLA-DR | Brilliant Violet 421 | Biolegend | 307636 | 5:100 |

Supplemental Table 3. Description of flow cytometry antibody panels used in this study.

| **Gene** | **Alternative Name** | **Receptor** | **Function** | **TNFα** | **IFNγ** |
| --- | --- | --- | --- | --- | --- |
| *CD40* |  | CD40L | activating | ↑ | ↑ |
| *CD74* | Invariant Chain |  | stabilizes MHC II | ↑ | ↑ |
| *HLA-B* |  | TCR |  | ↑ | ↑ |
| *HLA-C* |  | TCR |  | ↑ | ↑ |
| *HLA-E* |  | NKG2A/B/C | inhibitory ligand for NK cells | ↑ | ↑ |
| *IL15* |  |  | proliferative for T cells and NK cells | ↑ | ↑ |
| *PDCD1LG2* | PD-L2 | PD-1 |  | ↑ | ↑ |
| *PSMB10* |  |  | immunoproteasome; replaces PSMB7 subunit, processing of MHC I peptides | ↑ | ↑ |
| *PSMB5* |  |  | constitutive proteasome | ↑ | ↑ |
| *PSMB7* |  |  | constitutive proteasome | ↑ | ↑ |
| *PSMB8* |  |  | immunoproteasome; replaces PSMB5 subunit, processing of MHC I peptides | ↑ | ↑ |
| *PSMB9* |  |  | immunoproteasome; replaces PSMB6 subunit, processing of MHC I peptides | ↑ | ↑ |
| *TAP1* |  |  | facilitates peptide transport to the ER for MHC I presentation | ↑ | ↑ |
| *TAP2* |  |  | facilitates peptide transport to the ER for MHC I presentation | ↑ | ↑ |
| *TAPBP* |  |  | facilitates peptide transport to the ER for MHC I presentation | ↑ | ↑ |
| *TNFRSF14* | HVEM/LIGHTR |  | cell-cell contact between APC and lymphocytes; negative regulatory role | ↑ | ↑ |
| *TNFSF10* | TRAIL |  | pro-apoptotic cytokine for transformed cells | ↑ | ↑ |
| *TNFSF13B* | BAFF/BLyS |  | stimulatory for T cell and B cells | ↑ | ↑ |
| *CD83* |  |  | marker of activated/mature APC | ↑ | - |
| *ICOSLG* | ICOS ligand | ICOS | costimulatory for T cells, including memory T cells | ↑ | - |
| *IL6* |  |  |  | ↑ | - |
| *PSMD7* |  |  | proteasome | ↑ | - |
| *TNFRSF9* | 4-1BB (CD137) |  | activation of T cells | ↑ | - |
| *TNFSF15* | VEGI/TL1 |  | autocrine anti-angiogenic cytokine | ↑ | - |
| *TNFSF4* | OX40L | OX40 | costimulatory for T cells | ↑ | - |
| *CD274* | PD-L1 | PD-1 | inhibitory | - | ↑ |
| *CEACAM1* |  | TIM3 | inhibitory | - | ↑ |
| *CIITA* |  |  | transcription factor regulating MHC II | - | ↑ |
| *HLA-DMA* |  |  | facilitates peptide loading to MHC II | - | ↑ |
| *HLA-DMB* |  |  | facilitates peptide loading to MHC II | - | ↑ |
| *HLA-DOB* |  |  | facilitates peptide loading to MHC II | - | ↑ |
| *HLA-DPA1* |  | TCR |  | - | ↑ |
| *HLA-DPB1* |  | TCR |  | - | ↑ |
| *HLA-DQA1* |  | TCR |  | - | ↑ |
| *HLA-DQB1* |  | TCR |  | - | ↑ |
| *HLA-DRA* |  | TCR |  | - | ↑ |
| *HLA-DRB1* |  | TCR |  | - | ↑ |
| *HLA-DRB3* |  | TCR |  | - | ↑ |
| *IDO1* | indoleamine 2,3-dioxygenase (IDO) |  | promotes immune T cell tolerance | - | ↑ |
| *CXCL12* | SDF-1 |  | *decreases monocyte adherence to surfaces coated with ICAM-1* | ↓ | ↓ |
| *IL32* |  |  |  | ↓ | ↓ |

Supplemental Table 4. List of genes changed by TNFα and/or IFNγ in endothelial cells.

| **Genes unchanged in endothelial cells by TNFα or IFNγ** |
| --- |
| CD276 |
| CD58 (LFA-3) |
| CD80 |
| CD80 |
| CD86 |
| CD86 |
| IFNγ1 |
| IFNγ2 |
| IL10 |
| IL12A |
| IL12B |
| IL13 |
| IL16 |
| IL17A |
| IL17B |
| IL17F |
| IL18 |
| IL19 |
| IL2 |
| IL20 |
| IL21 |
| IL22 |
| IL26 |
| IL27 |
| IL28A |
| IL29 |
| IL3 |
| IL4 |
| IL5 |
| IL7 |
| IL9 |
| TNF |
| TNFRSF8 |
| TNFSF11 |
| TNFSF8 |

Supplemental Table 5. List of genes that were unchanged by TNFα or IFNγ in endothelial cells.
