# Supplementary material for: IFNγ, and to a Lesser Extent TNFα, Provokes a Sustained Endothelial Costimulatory Phenotype
Source: Front Immunol. 2021 Apr 15;12:648946. doi: 10.3389/fimmu.2021.648946 (PMC8082142; doi:10.3389/fimmu.2021.648946)
Supplement: Supplementary file 2 [file Presentation_1.pptx]

## Slide 1
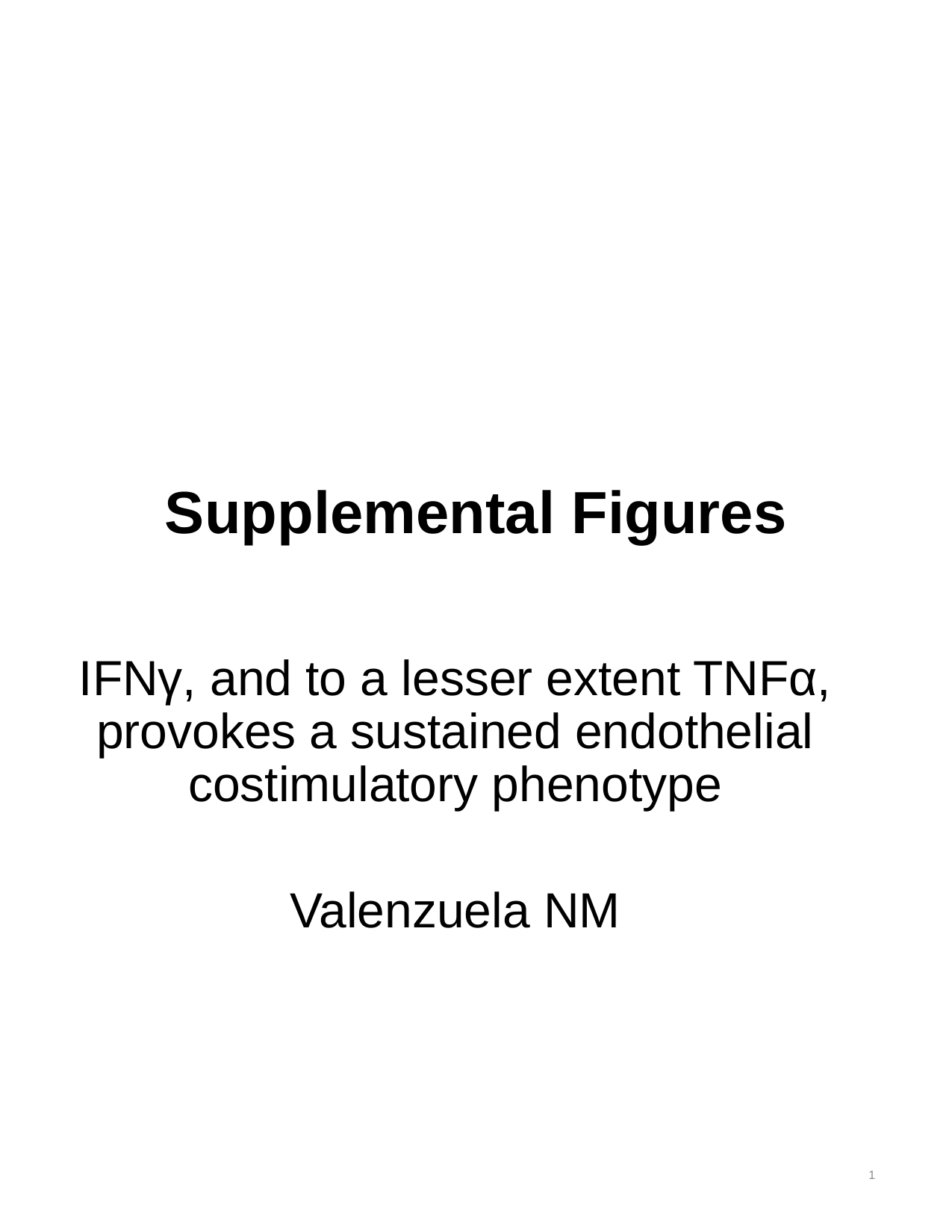

# Supplemental Figures
IFNγ, and to a lesser extent TNFα, provokes a sustained endothelial costimulatory phenotype
Valenzuela NM
1

## Slide 2
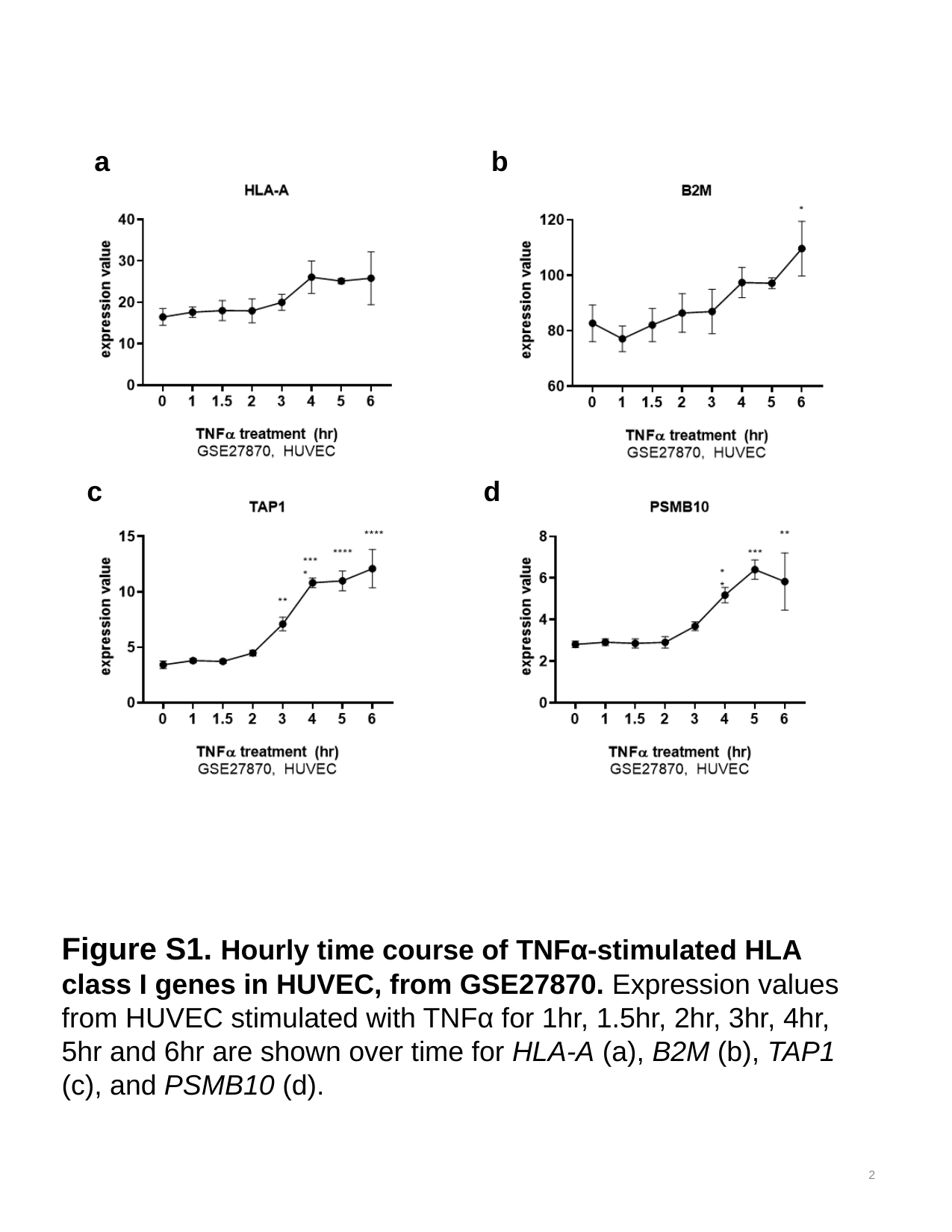

a
b
c
d
Figure S1. Hourly time course of TNFα-stimulated HLA class I genes in HUVEC, from GSE27870. Expression values from HUVEC stimulated with TNFα for 1hr, 1.5hr, 2hr, 3hr, 4hr, 5hr and 6hr are shown over time for HLA-A (a), B2M (b), TAP1 (c), and PSMB10 (d).
2

## Slide 3
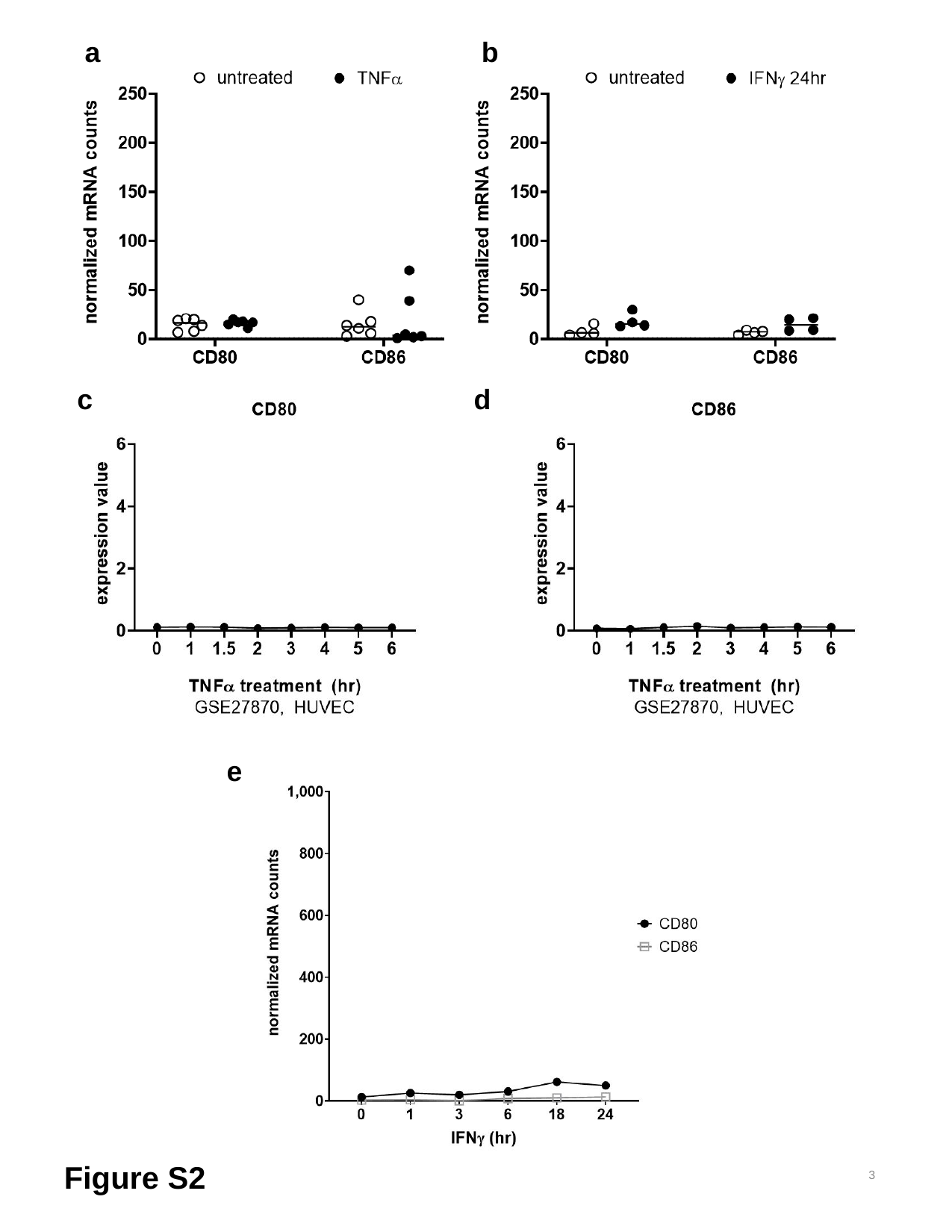

a
b
c
d
e
3
Figure S2

## Slide 4
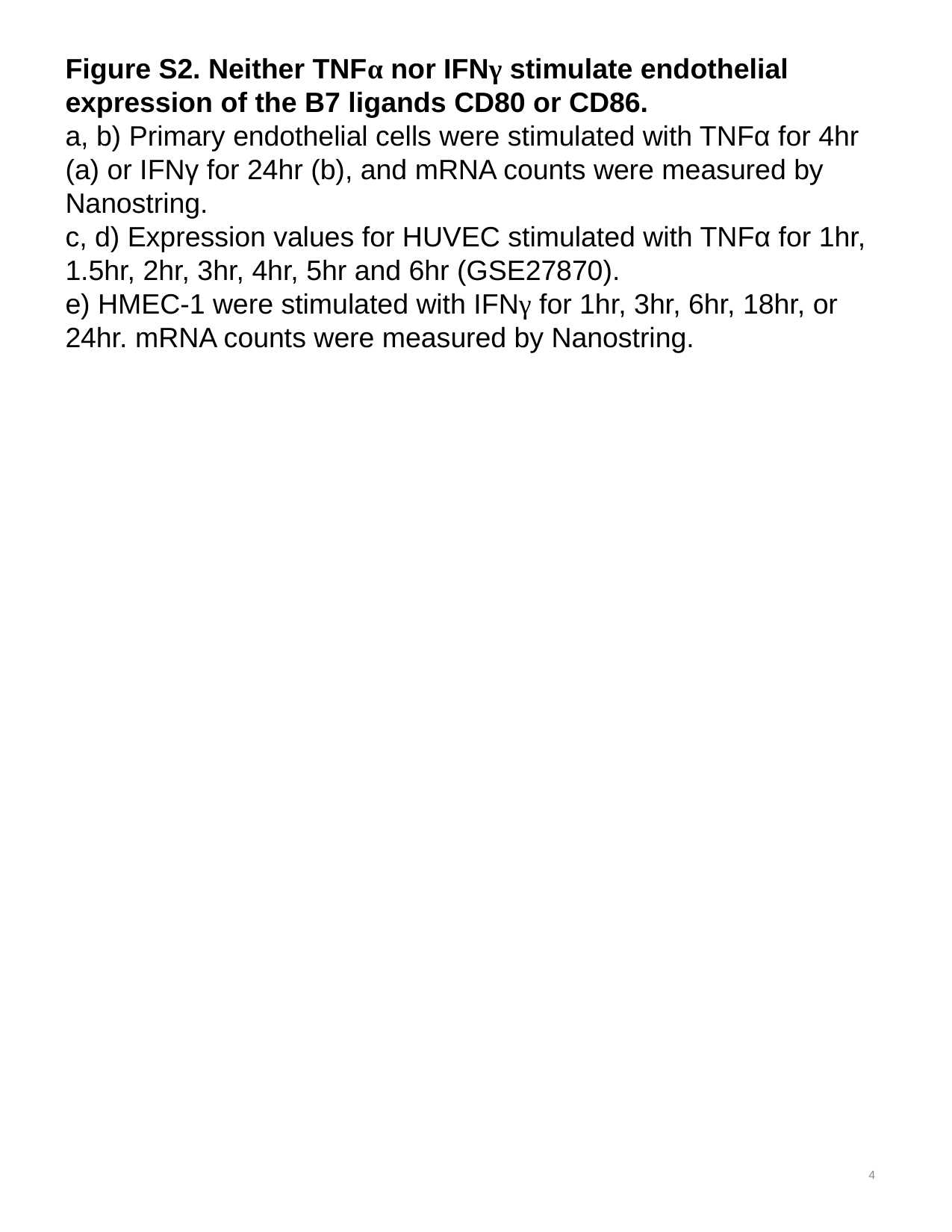

Figure S2. Neither TNFα nor IFNγ stimulate endothelial expression of the B7 ligands CD80 or CD86.
a, b) Primary endothelial cells were stimulated with TNFα for 4hr (a) or IFNγ for 24hr (b), and mRNA counts were measured by Nanostring.
c, d) Expression values for HUVEC stimulated with TNFα for 1hr, 1.5hr, 2hr, 3hr, 4hr, 5hr and 6hr (GSE27870).
e) HMEC-1 were stimulated with IFNγ for 1hr, 3hr, 6hr, 18hr, or 24hr. mRNA counts were measured by Nanostring.
4

## Slide 5
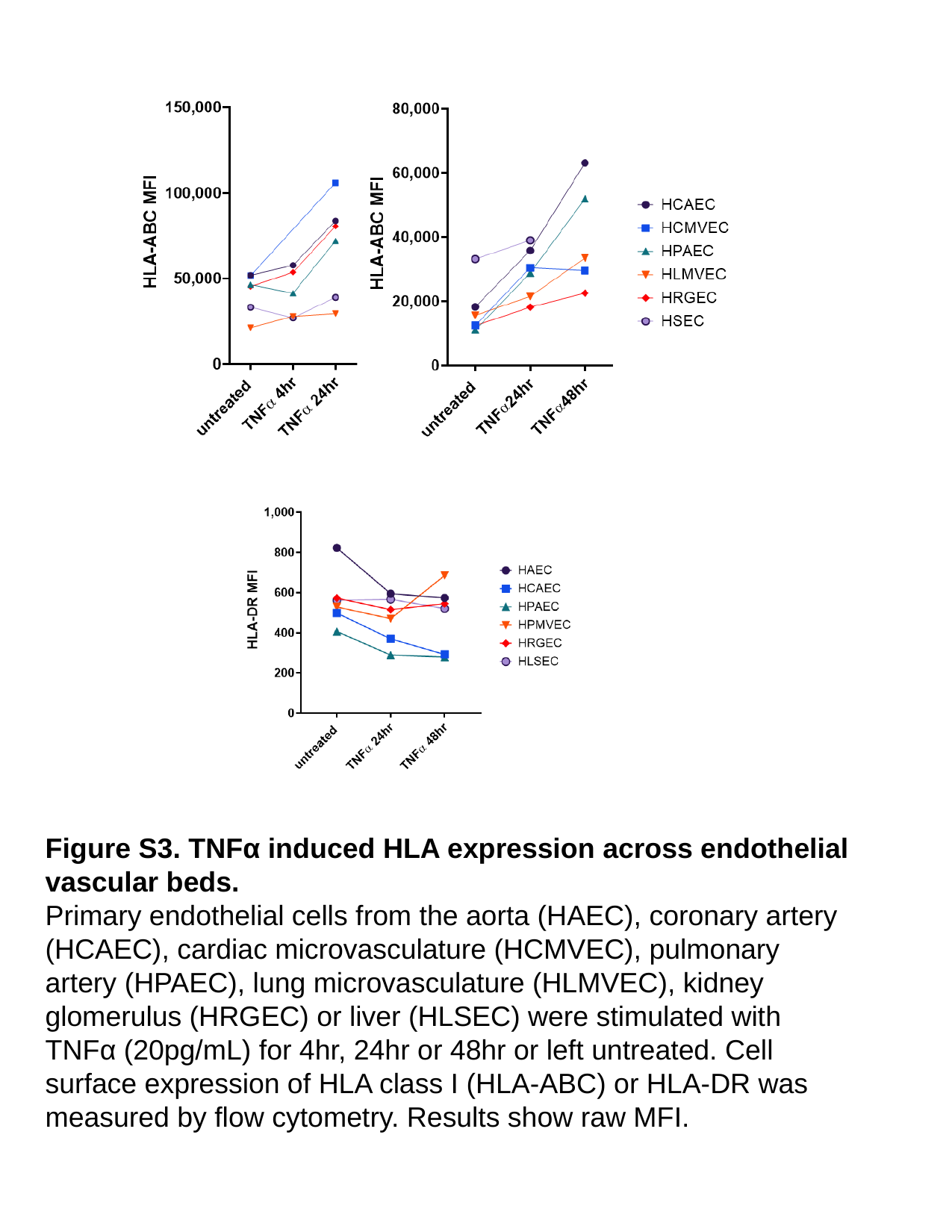

Figure S3. TNFα induced HLA expression across endothelial vascular beds.
Primary endothelial cells from the aorta (HAEC), coronary artery (HCAEC), cardiac microvasculature (HCMVEC), pulmonary artery (HPAEC), lung microvasculature (HLMVEC), kidney glomerulus (HRGEC) or liver (HLSEC) were stimulated with TNFα (20pg/mL) for 4hr, 24hr or 48hr or left untreated. Cell surface expression of HLA class I (HLA-ABC) or HLA-DR was measured by flow cytometry. Results show raw MFI.

## Slide 6
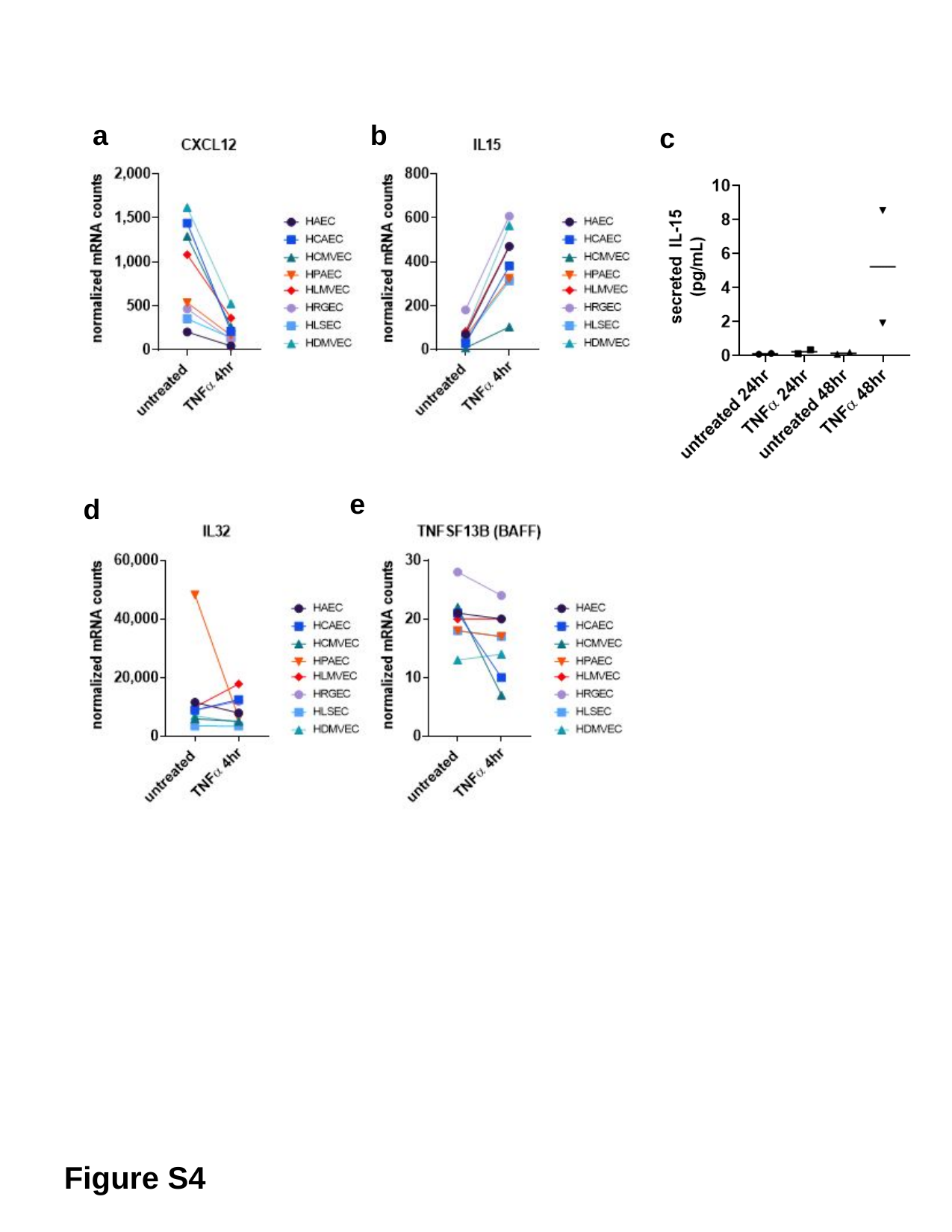

a
b
c
e
d
Figure S4

## Slide 7
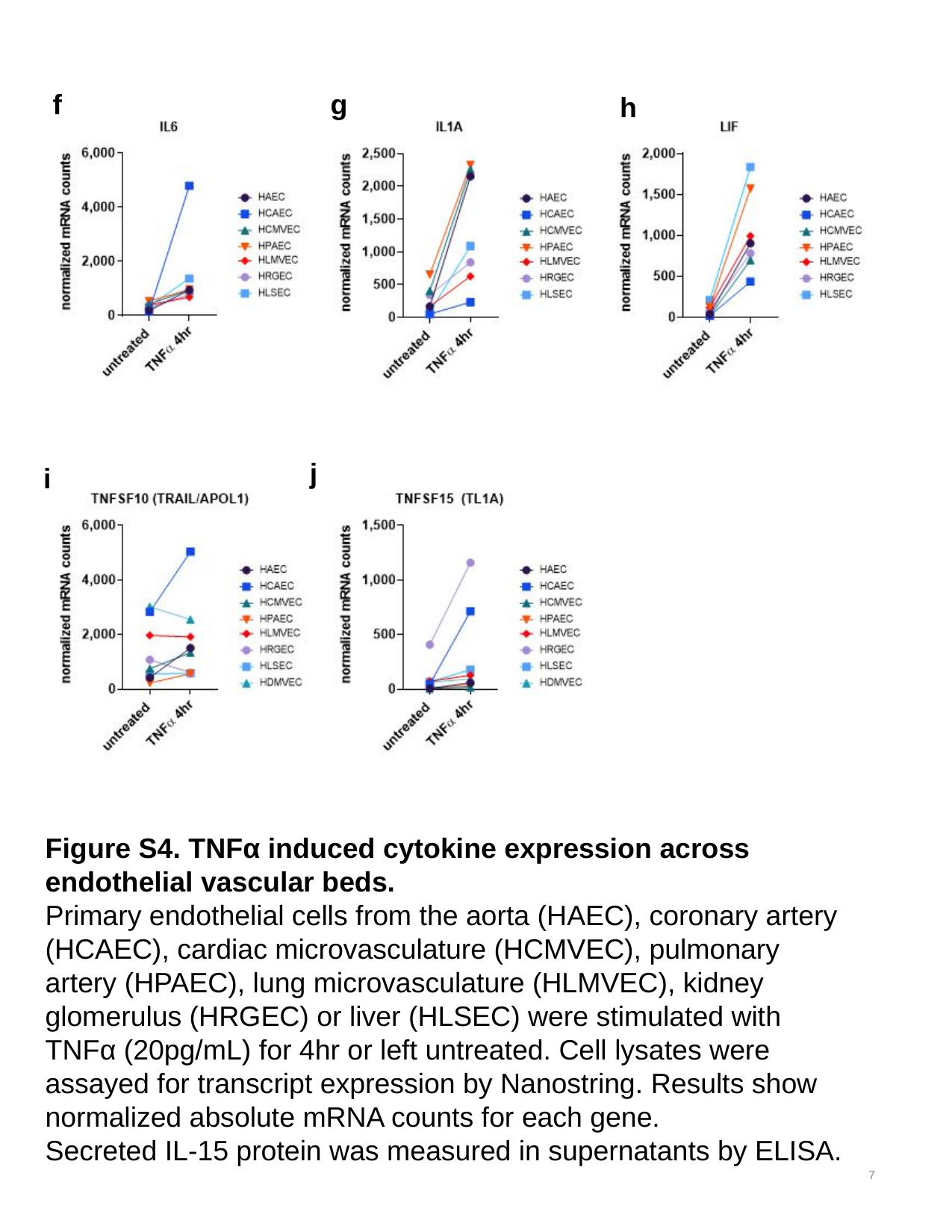

f
g
h
j
i
Figure S4. TNFα induced cytokine expression across endothelial vascular beds.
Primary endothelial cells from the aorta (HAEC), coronary artery (HCAEC), cardiac microvasculature (HCMVEC), pulmonary artery (HPAEC), lung microvasculature (HLMVEC), kidney glomerulus (HRGEC) or liver (HLSEC) were stimulated with TNFα (20pg/mL) for 4hr or left untreated. Cell lysates were assayed for transcript expression by Nanostring. Results show normalized absolute mRNA counts for each gene.
Secreted IL-15 protein was measured in supernatants by ELISA.
7

## Slide 8
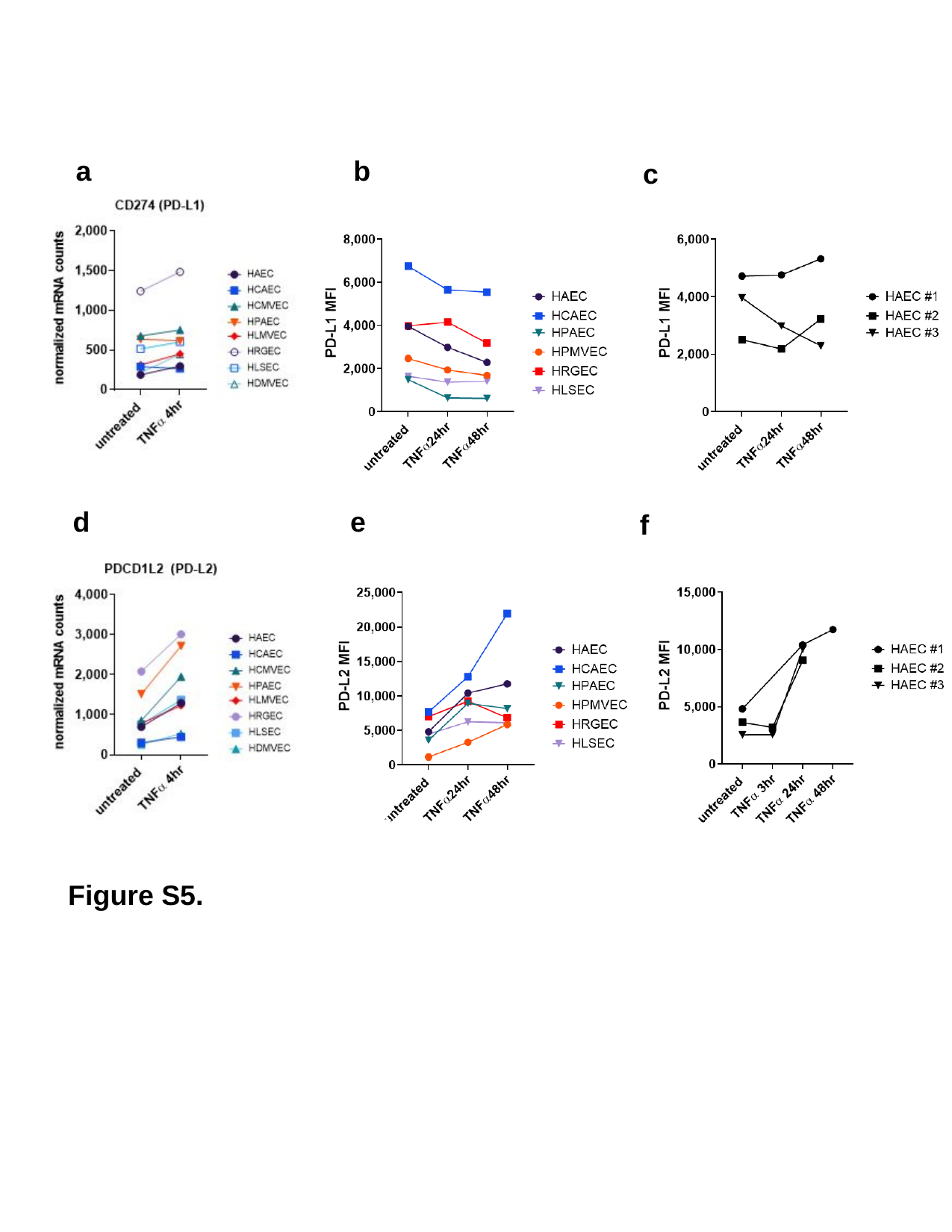

a
b
c
d
e
f
Figure S5.

## Slide 9
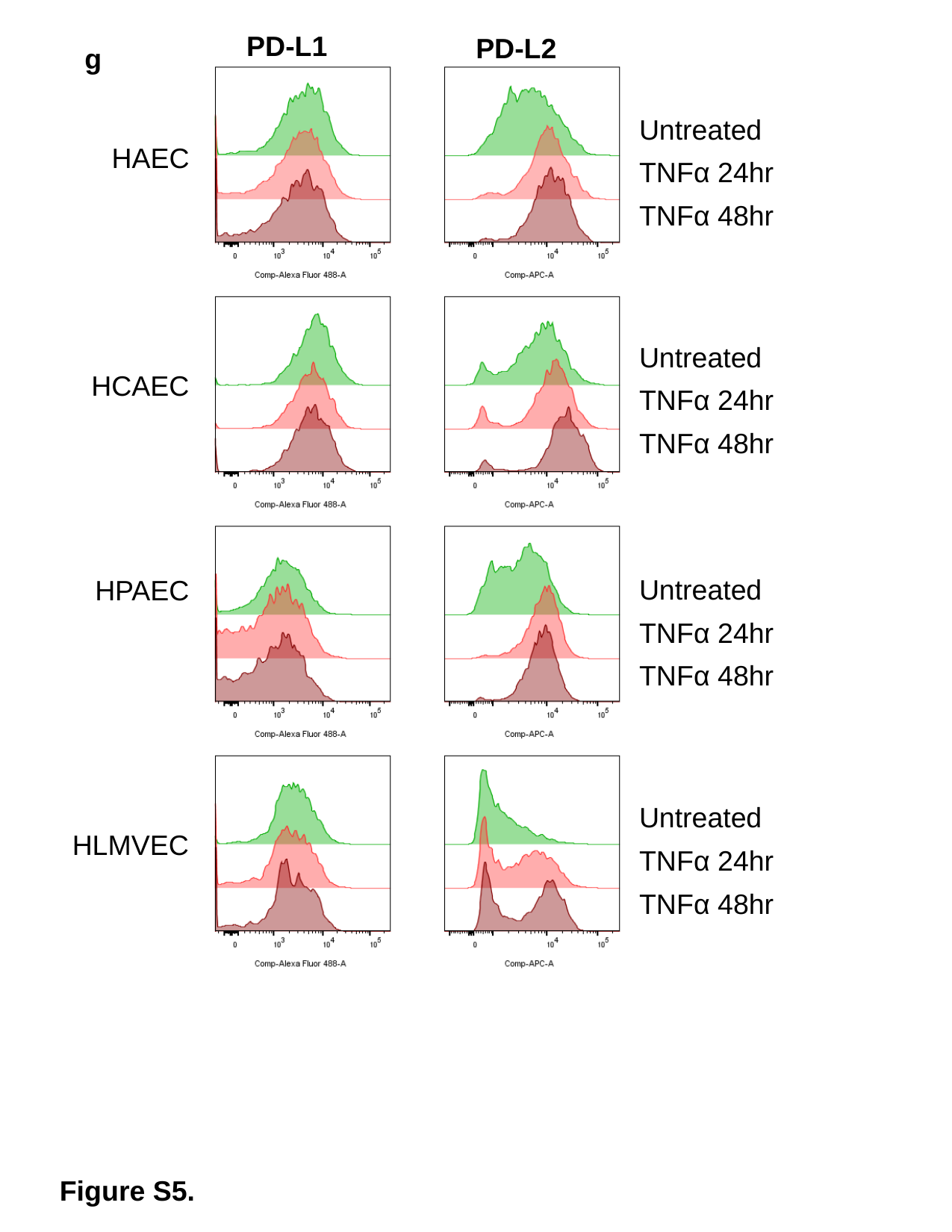

PD-L1
PD-L2
g
Untreated
TNFα 24hr
TNFα 48hr
HAEC
Untreated
TNFα 24hr
TNFα 48hr
HCAEC
Untreated
TNFα 24hr
TNFα 48hr
HPAEC
Untreated
TNFα 24hr
TNFα 48hr
HLMVEC
Figure S5.

## Slide 10
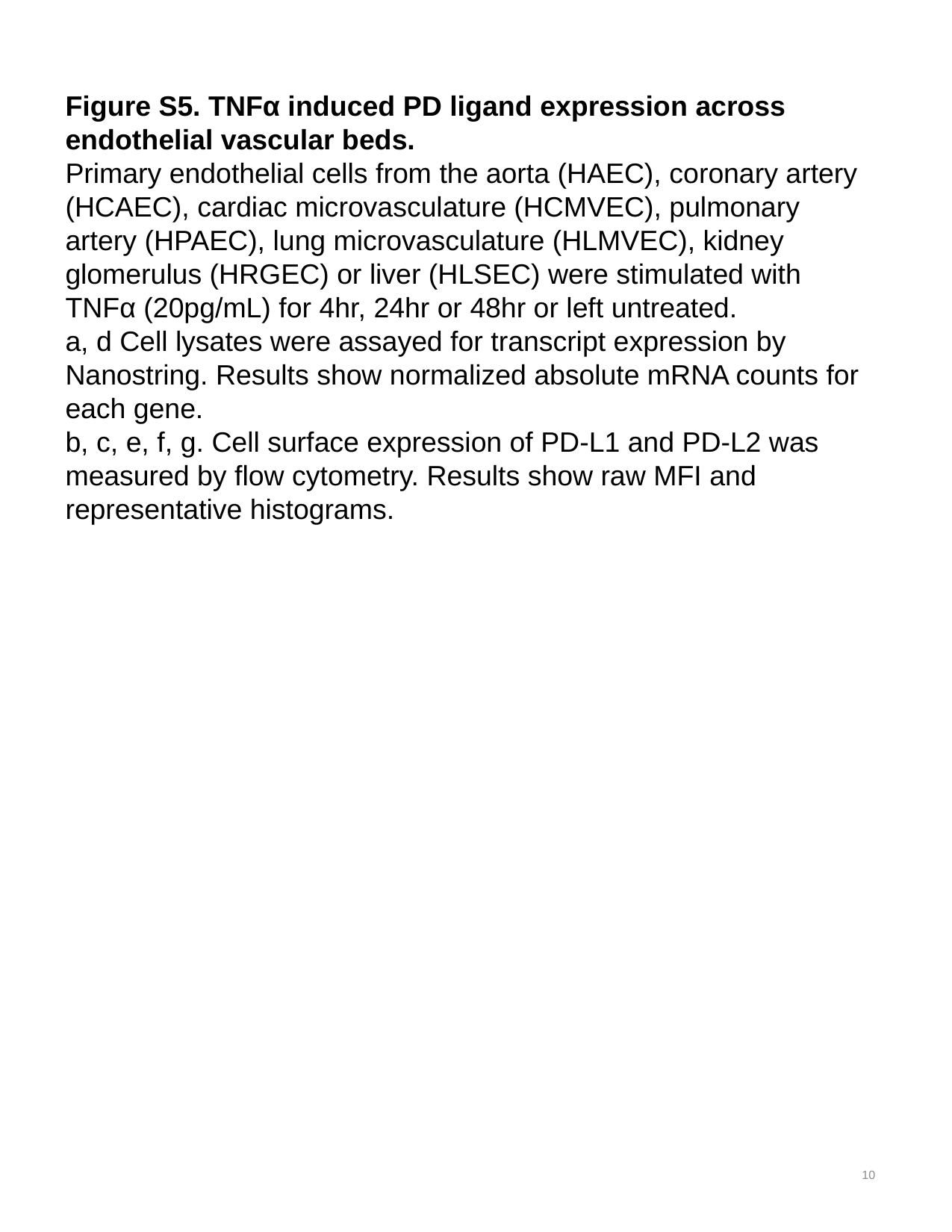

Figure S5. TNFα induced PD ligand expression across endothelial vascular beds.
Primary endothelial cells from the aorta (HAEC), coronary artery (HCAEC), cardiac microvasculature (HCMVEC), pulmonary artery (HPAEC), lung microvasculature (HLMVEC), kidney glomerulus (HRGEC) or liver (HLSEC) were stimulated with TNFα (20pg/mL) for 4hr, 24hr or 48hr or left untreated.
a, d Cell lysates were assayed for transcript expression by Nanostring. Results show normalized absolute mRNA counts for each gene.
b, c, e, f, g. Cell surface expression of PD-L1 and PD-L2 was measured by flow cytometry. Results show raw MFI and representative histograms.
10

## Slide 11
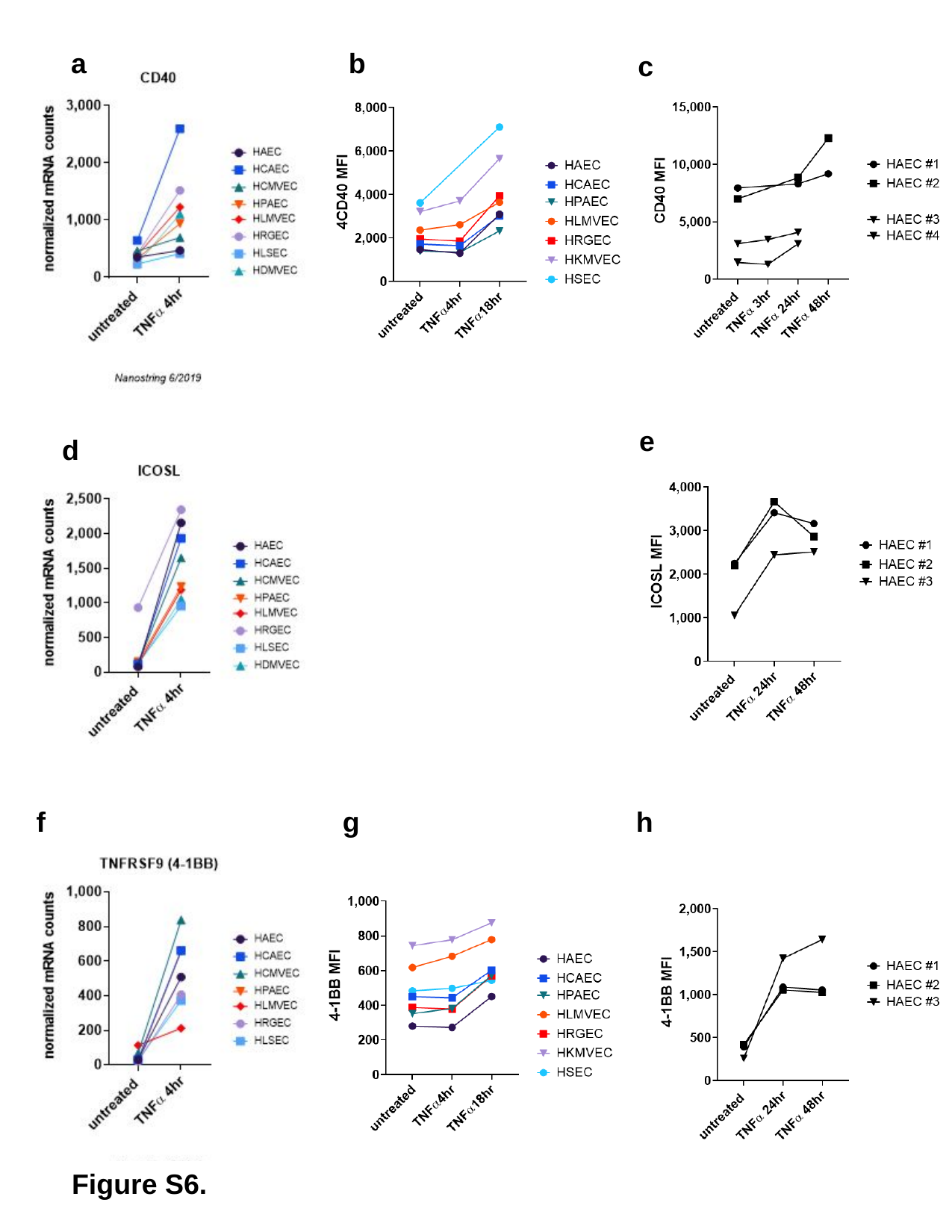

a
b
c
e
d
f
g
h
Figure S6.

## Slide 12
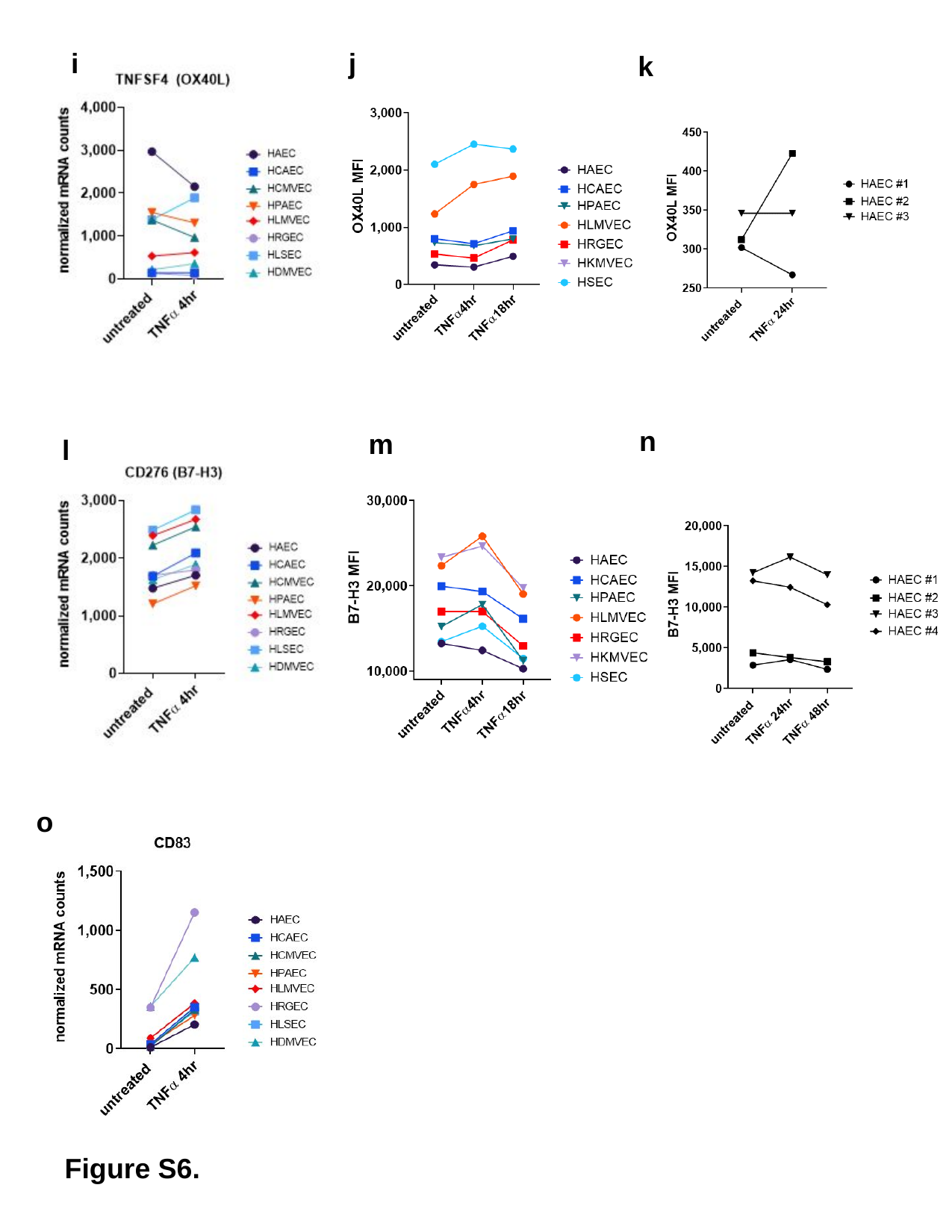

i
j
k
n
m
l
o
Figure S6.

## Slide 13
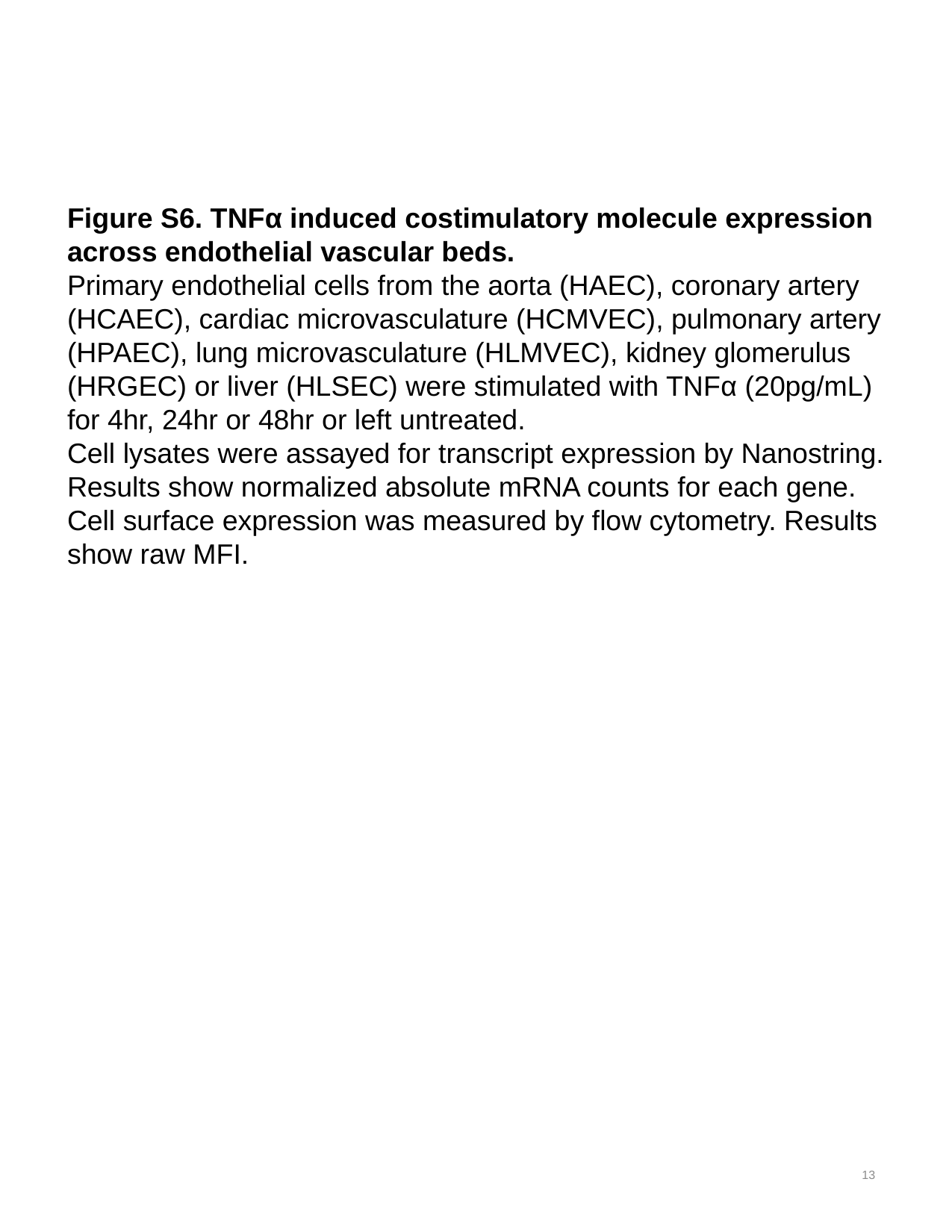

Figure S6. TNFα induced costimulatory molecule expression across endothelial vascular beds.
Primary endothelial cells from the aorta (HAEC), coronary artery (HCAEC), cardiac microvasculature (HCMVEC), pulmonary artery (HPAEC), lung microvasculature (HLMVEC), kidney glomerulus (HRGEC) or liver (HLSEC) were stimulated with TNFα (20pg/mL) for 4hr, 24hr or 48hr or left untreated.
Cell lysates were assayed for transcript expression by Nanostring. Results show normalized absolute mRNA counts for each gene.
Cell surface expression was measured by flow cytometry. Results show raw MFI.
13

## Slide 14
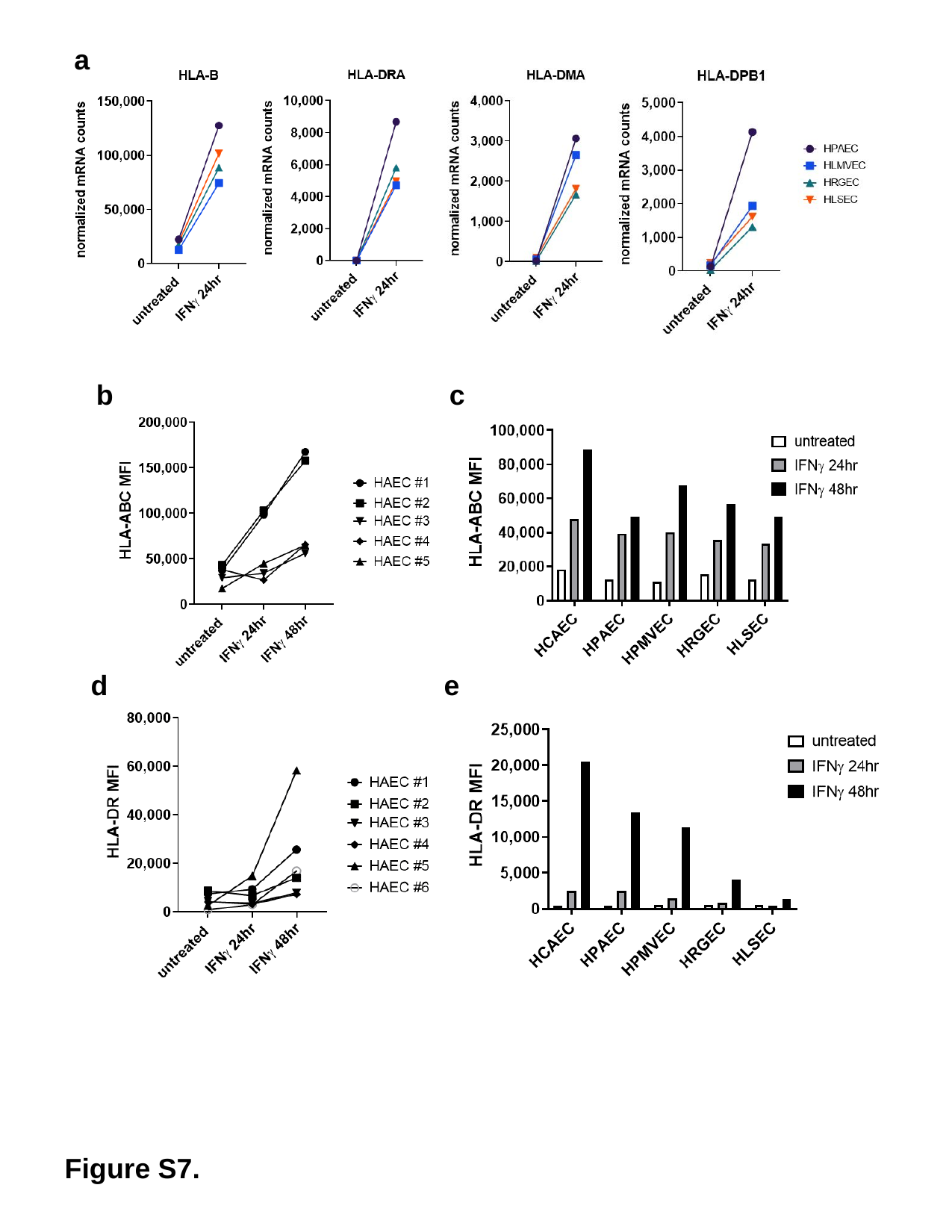

a
b
c
d
e
Figure S7.

## Slide 15
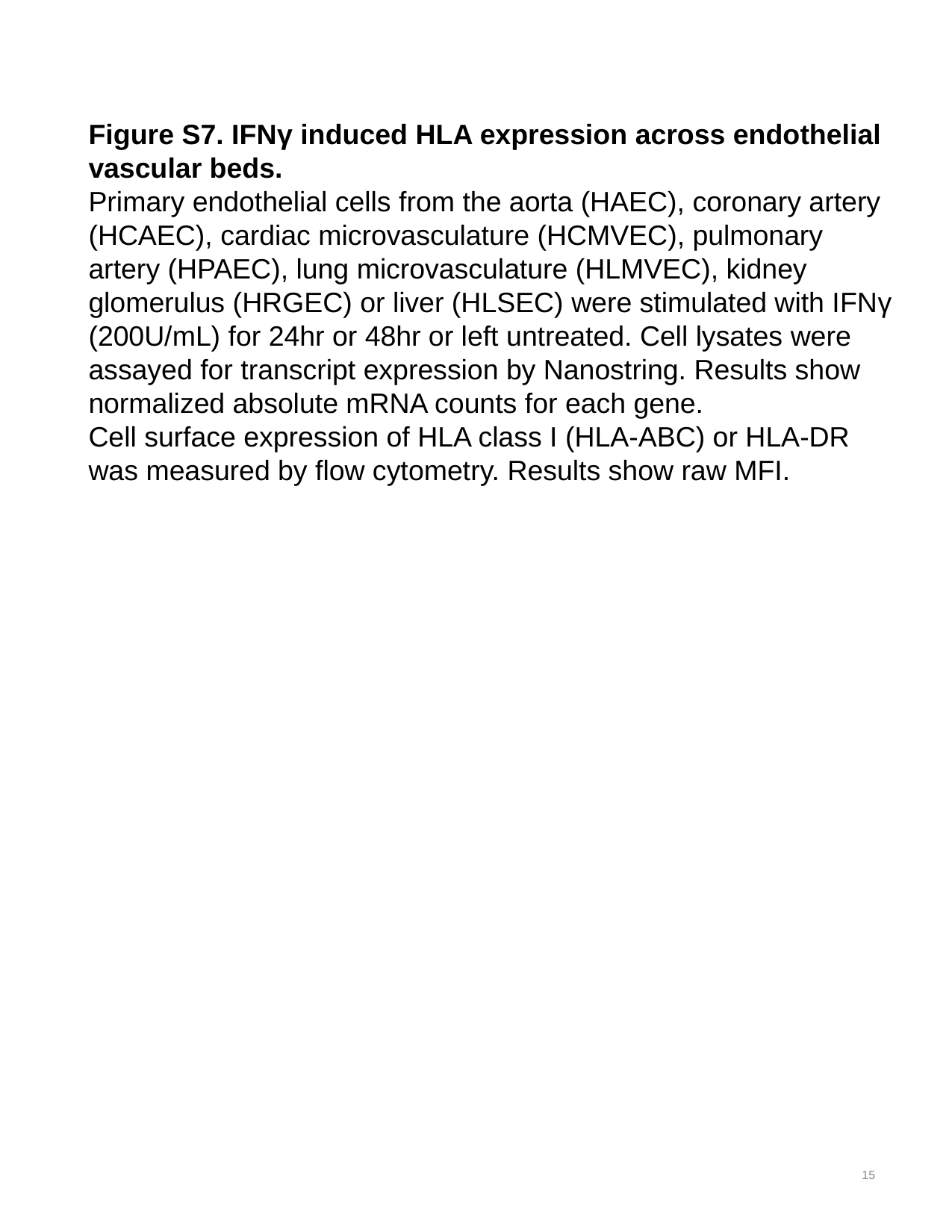

Figure S7. IFNγ induced HLA expression across endothelial vascular beds.
Primary endothelial cells from the aorta (HAEC), coronary artery (HCAEC), cardiac microvasculature (HCMVEC), pulmonary artery (HPAEC), lung microvasculature (HLMVEC), kidney glomerulus (HRGEC) or liver (HLSEC) were stimulated with IFNγ (200U/mL) for 24hr or 48hr or left untreated. Cell lysates were assayed for transcript expression by Nanostring. Results show normalized absolute mRNA counts for each gene.
Cell surface expression of HLA class I (HLA-ABC) or HLA-DR was measured by flow cytometry. Results show raw MFI.
15

## Slide 16
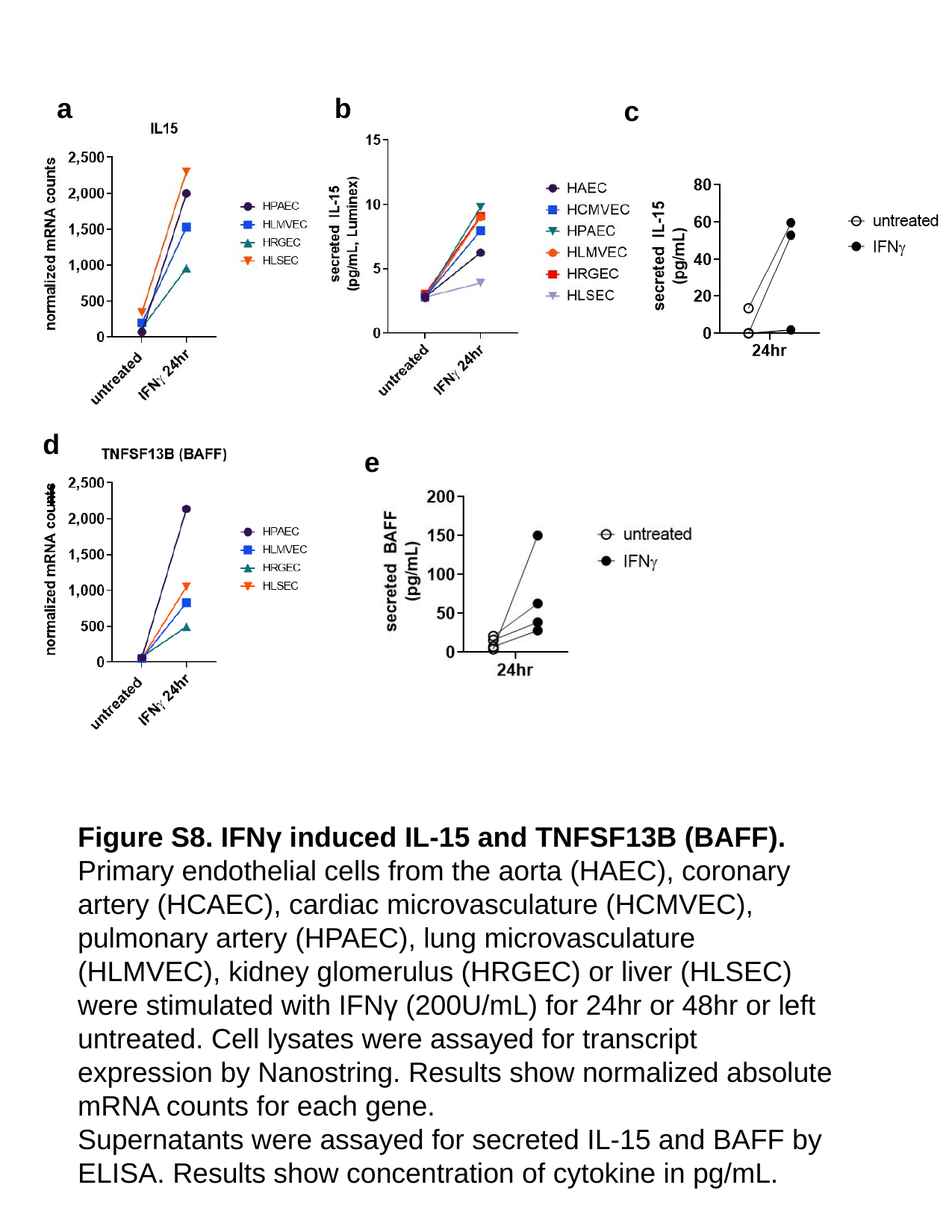

a
b
c
d
e
l
Figure S8. IFNγ induced IL-15 and TNFSF13B (BAFF). Primary endothelial cells from the aorta (HAEC), coronary artery (HCAEC), cardiac microvasculature (HCMVEC), pulmonary artery (HPAEC), lung microvasculature (HLMVEC), kidney glomerulus (HRGEC) or liver (HLSEC) were stimulated with IFNγ (200U/mL) for 24hr or 48hr or left untreated. Cell lysates were assayed for transcript expression by Nanostring. Results show normalized absolute mRNA counts for each gene.
Supernatants were assayed for secreted IL-15 and BAFF by ELISA. Results show concentration of cytokine in pg/mL.

## Slide 17
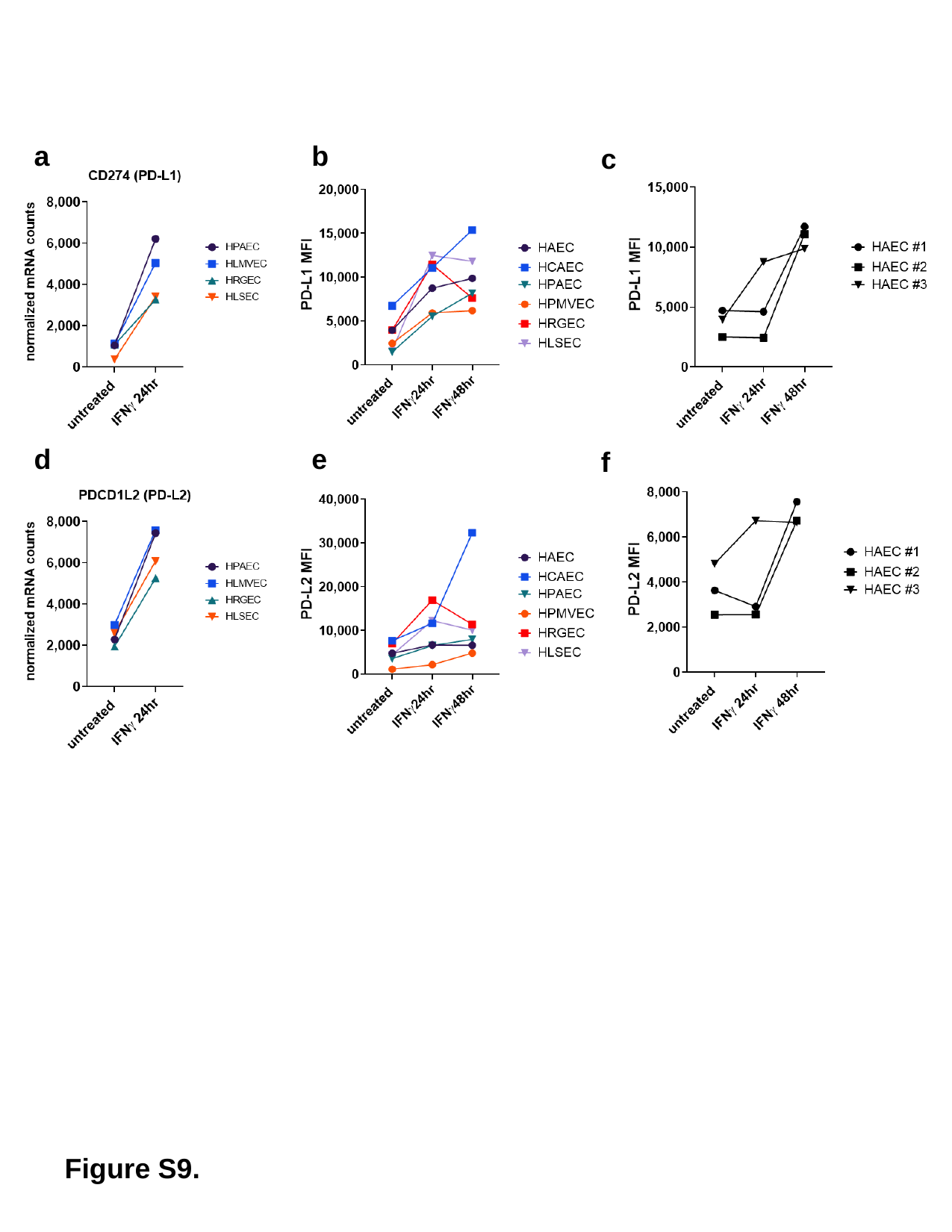

a
b
c
d
e
f
Figure S9.

## Slide 18
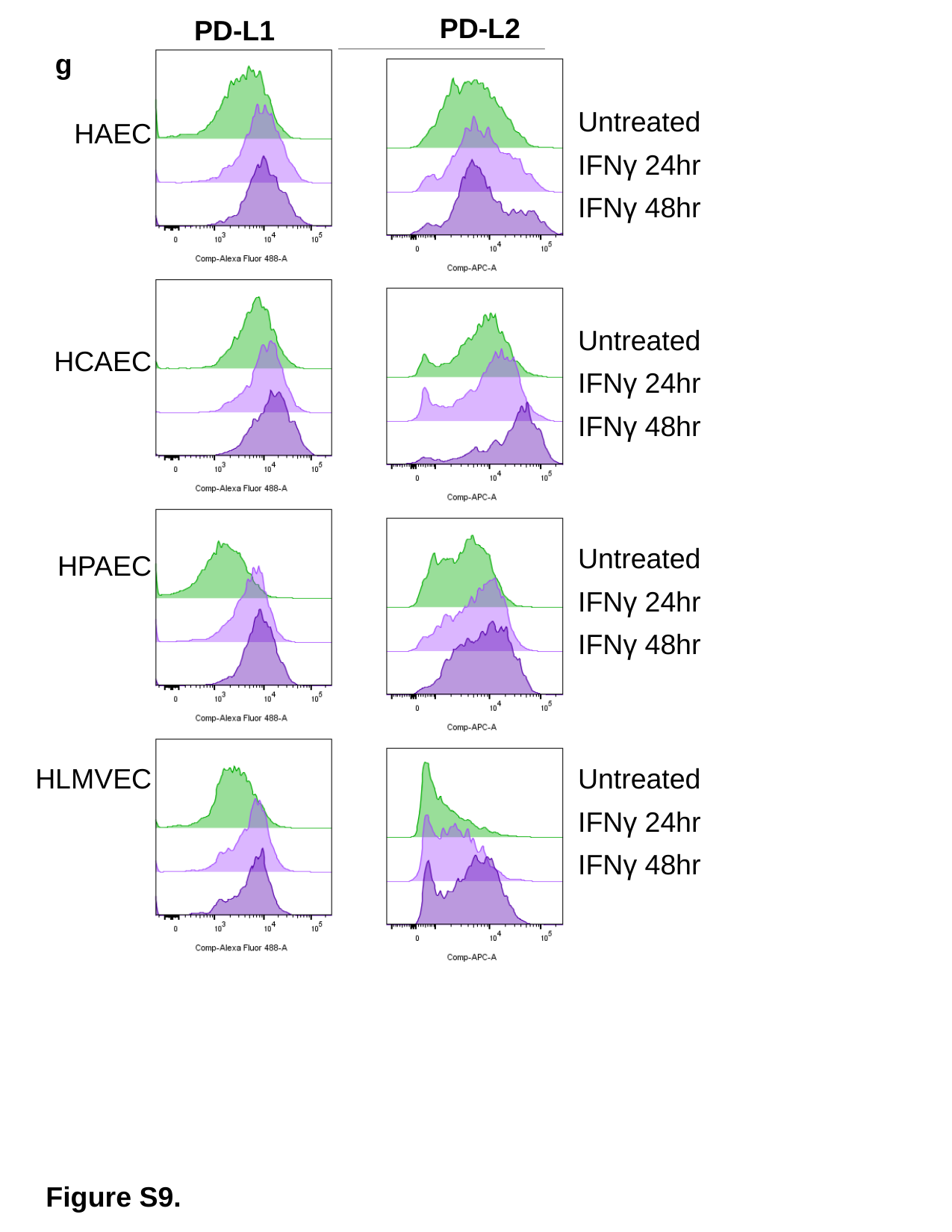

PD-L2
PD-L1
g
Untreated
IFNγ 24hr
IFNγ 48hr
HAEC
Untreated
IFNγ 24hr
IFNγ 48hr
HCAEC
Untreated
IFNγ 24hr
IFNγ 48hr
HPAEC
HLMVEC
Untreated
IFNγ 24hr
IFNγ 48hr
Figure S9.

## Slide 19
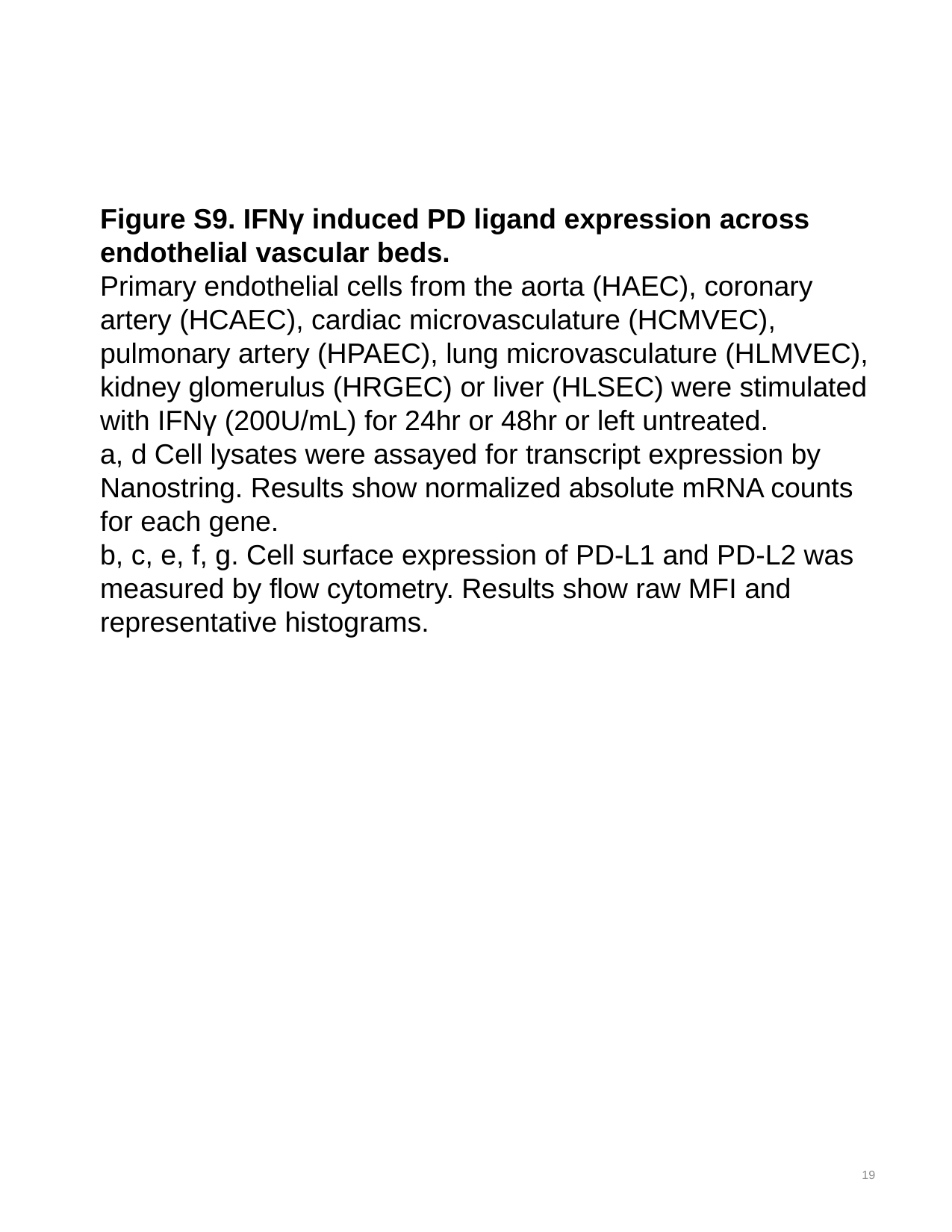

Figure S9. IFNγ induced PD ligand expression across endothelial vascular beds.
Primary endothelial cells from the aorta (HAEC), coronary artery (HCAEC), cardiac microvasculature (HCMVEC), pulmonary artery (HPAEC), lung microvasculature (HLMVEC), kidney glomerulus (HRGEC) or liver (HLSEC) were stimulated with IFNγ (200U/mL) for 24hr or 48hr or left untreated.
a, d Cell lysates were assayed for transcript expression by Nanostring. Results show normalized absolute mRNA counts for each gene.
b, c, e, f, g. Cell surface expression of PD-L1 and PD-L2 was measured by flow cytometry. Results show raw MFI and representative histograms.
19

## Slide 20
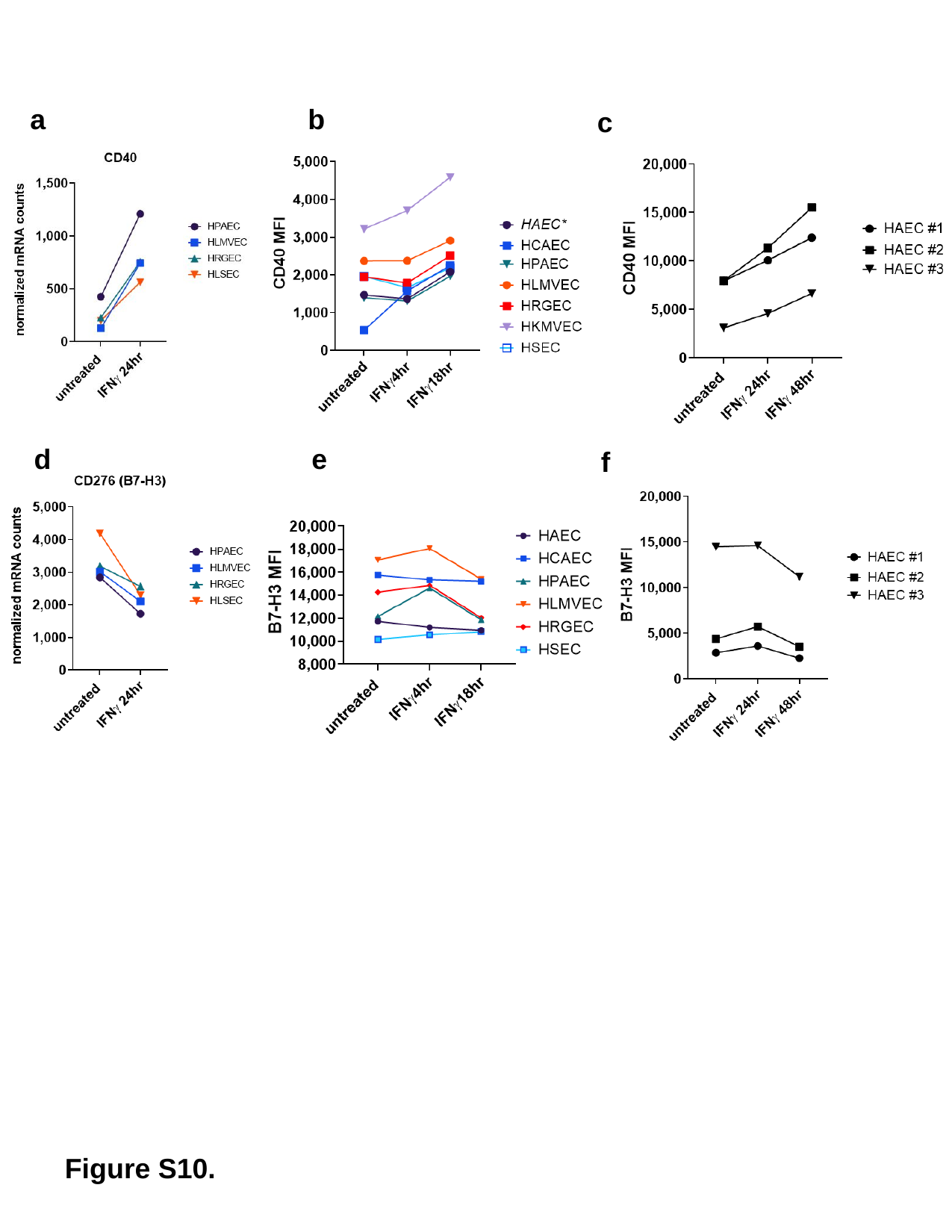

a
b
c
d
e
f
Figure S10.

## Slide 21
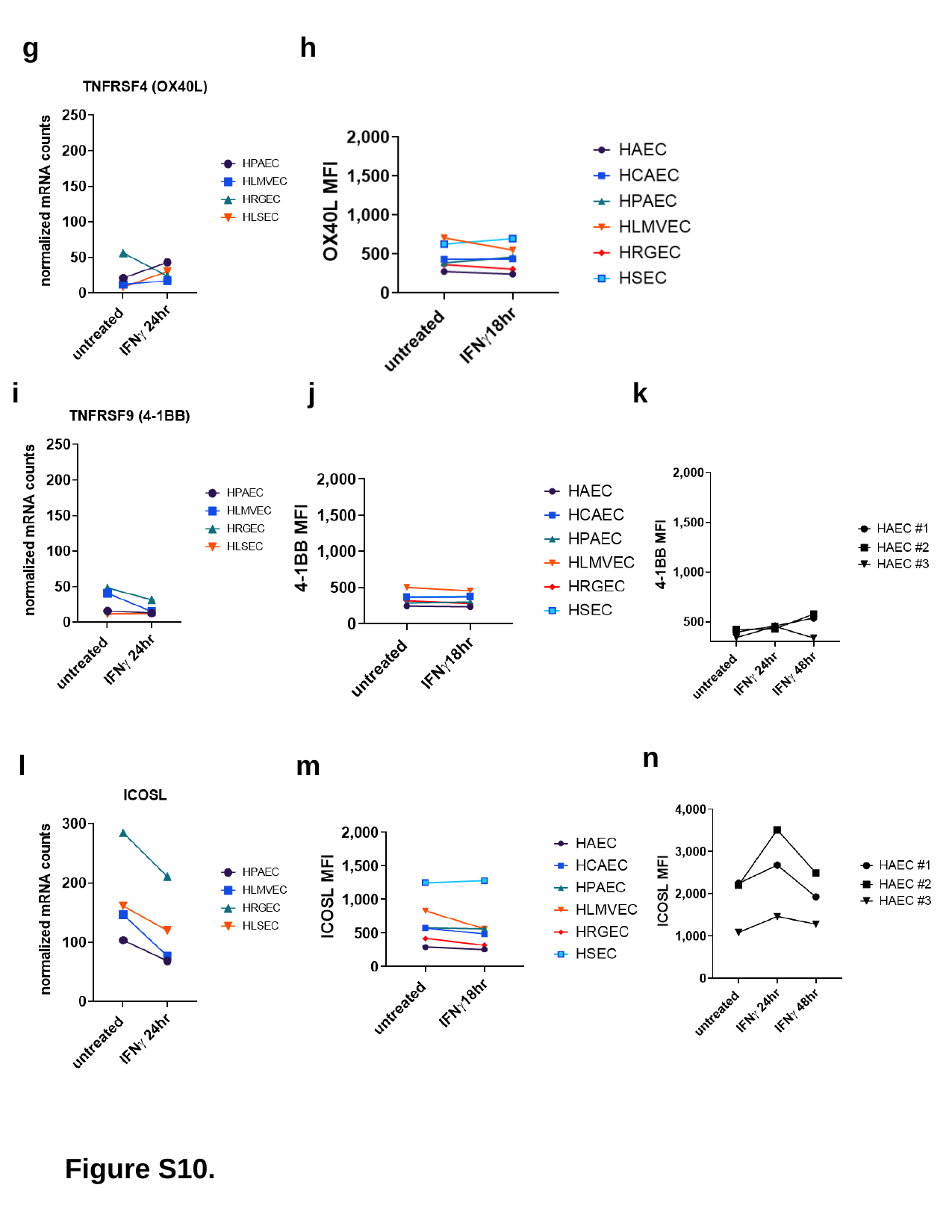

g
h
i
j
k
n
l
m
Figure S10.

## Slide 22
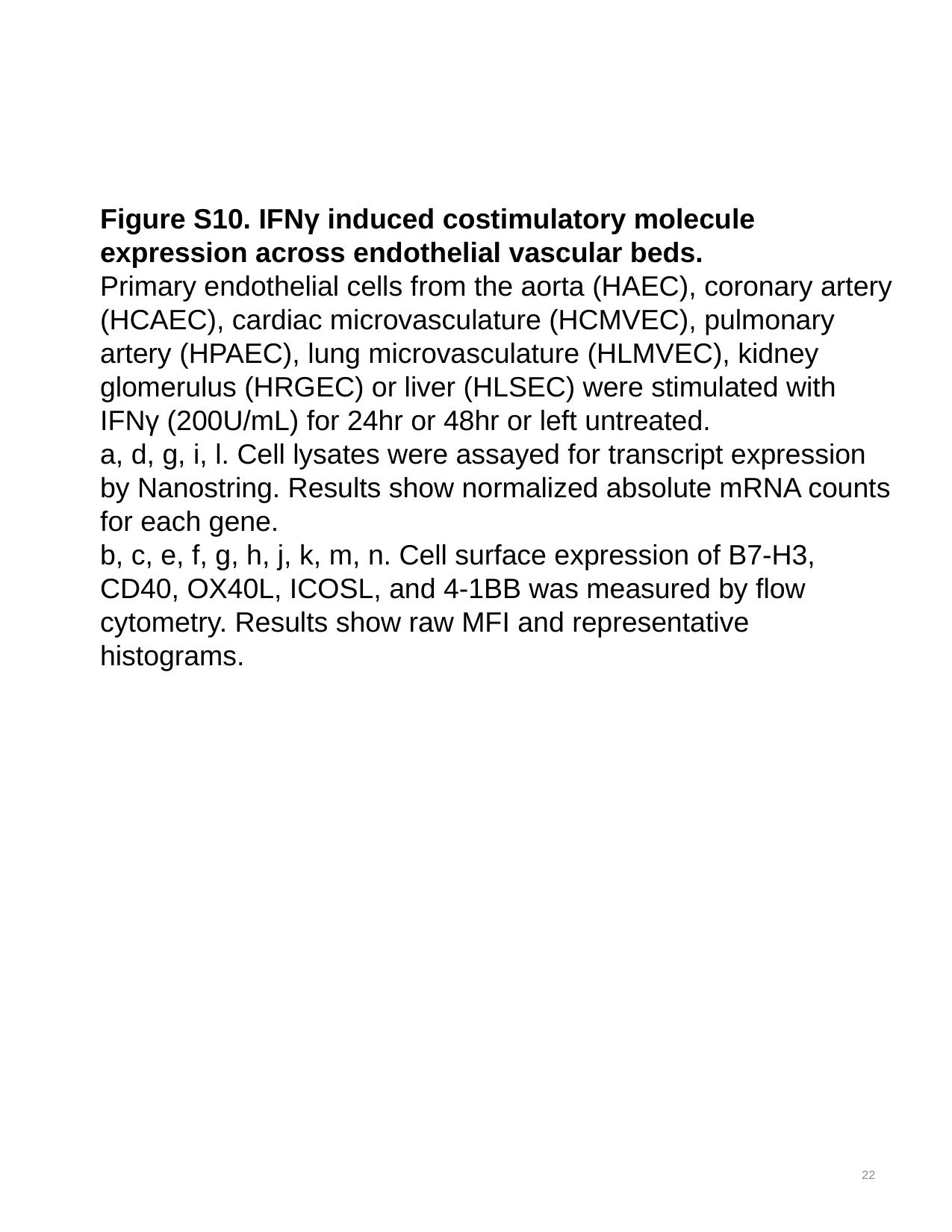

Figure S10. IFNγ induced costimulatory molecule expression across endothelial vascular beds.
Primary endothelial cells from the aorta (HAEC), coronary artery (HCAEC), cardiac microvasculature (HCMVEC), pulmonary artery (HPAEC), lung microvasculature (HLMVEC), kidney glomerulus (HRGEC) or liver (HLSEC) were stimulated with IFNγ (200U/mL) for 24hr or 48hr or left untreated.
a, d, g, i, l. Cell lysates were assayed for transcript expression by Nanostring. Results show normalized absolute mRNA counts for each gene.
b, c, e, f, g, h, j, k, m, n. Cell surface expression of B7-H3, CD40, OX40L, ICOSL, and 4-1BB was measured by flow cytometry. Results show raw MFI and representative histograms.
22

## Slide 23
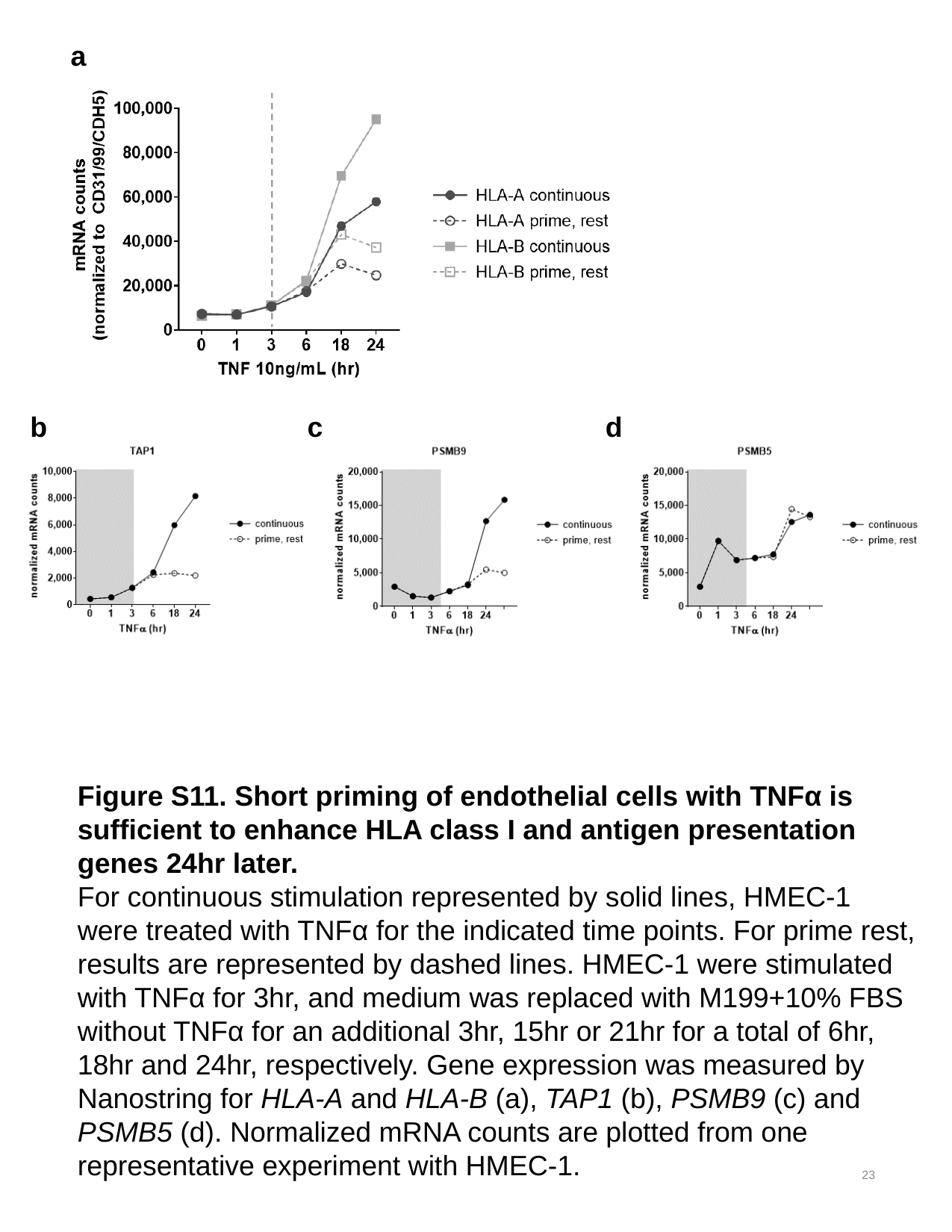

a
b
c
d
Figure S11. Short priming of endothelial cells with TNFα is sufficient to enhance HLA class I and antigen presentation genes 24hr later.
For continuous stimulation represented by solid lines, HMEC-1 were treated with TNFα for the indicated time points. For prime rest, results are represented by dashed lines. HMEC-1 were stimulated with TNFα for 3hr, and medium was replaced with M199+10% FBS without TNFα for an additional 3hr, 15hr or 21hr for a total of 6hr, 18hr and 24hr, respectively. Gene expression was measured by Nanostring for HLA-A and HLA-B (a), TAP1 (b), PSMB9 (c) and PSMB5 (d). Normalized mRNA counts are plotted from one representative experiment with HMEC-1.
23

## Slide 24
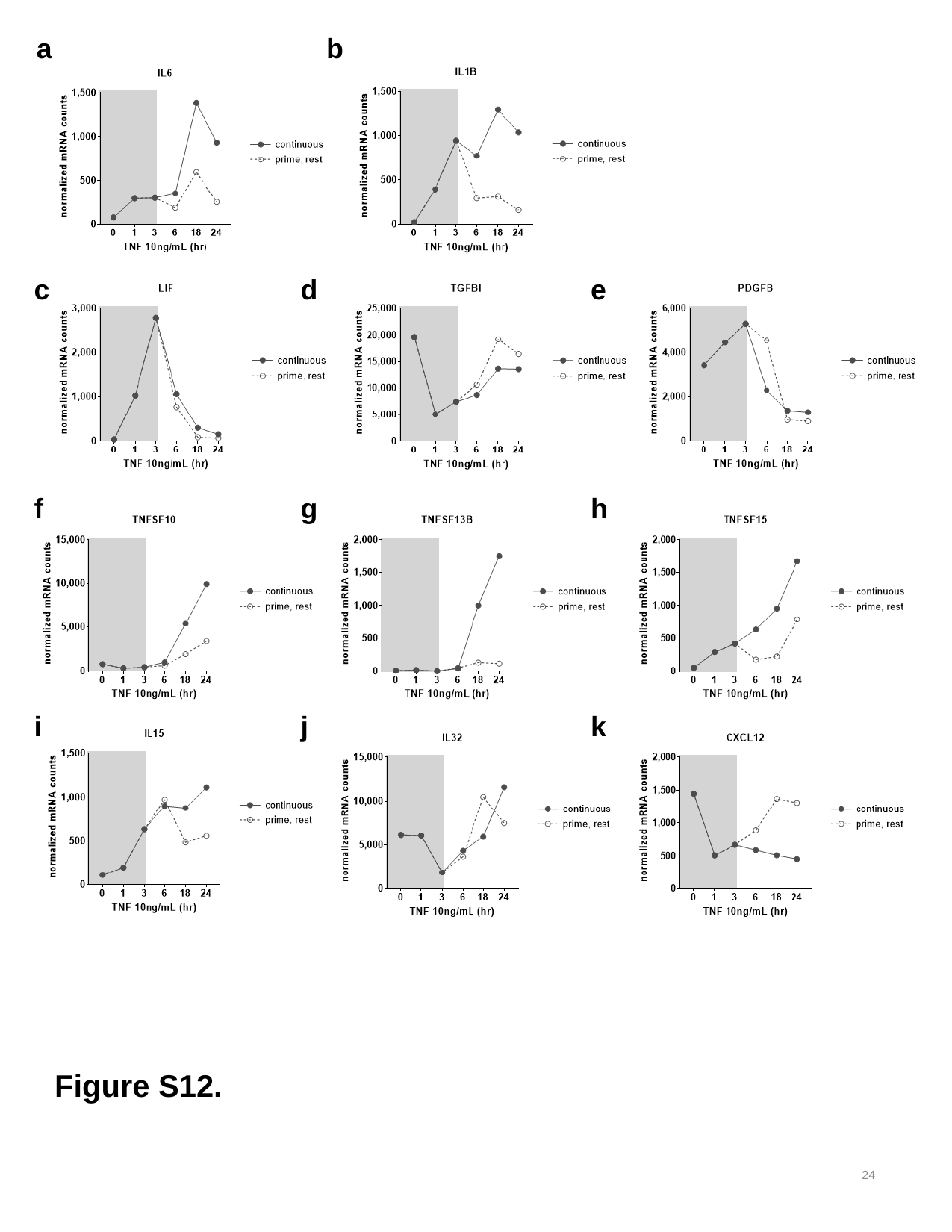

a
b
c
d
e
f
g
h
i
j
k
Figure S12.
24

## Slide 25
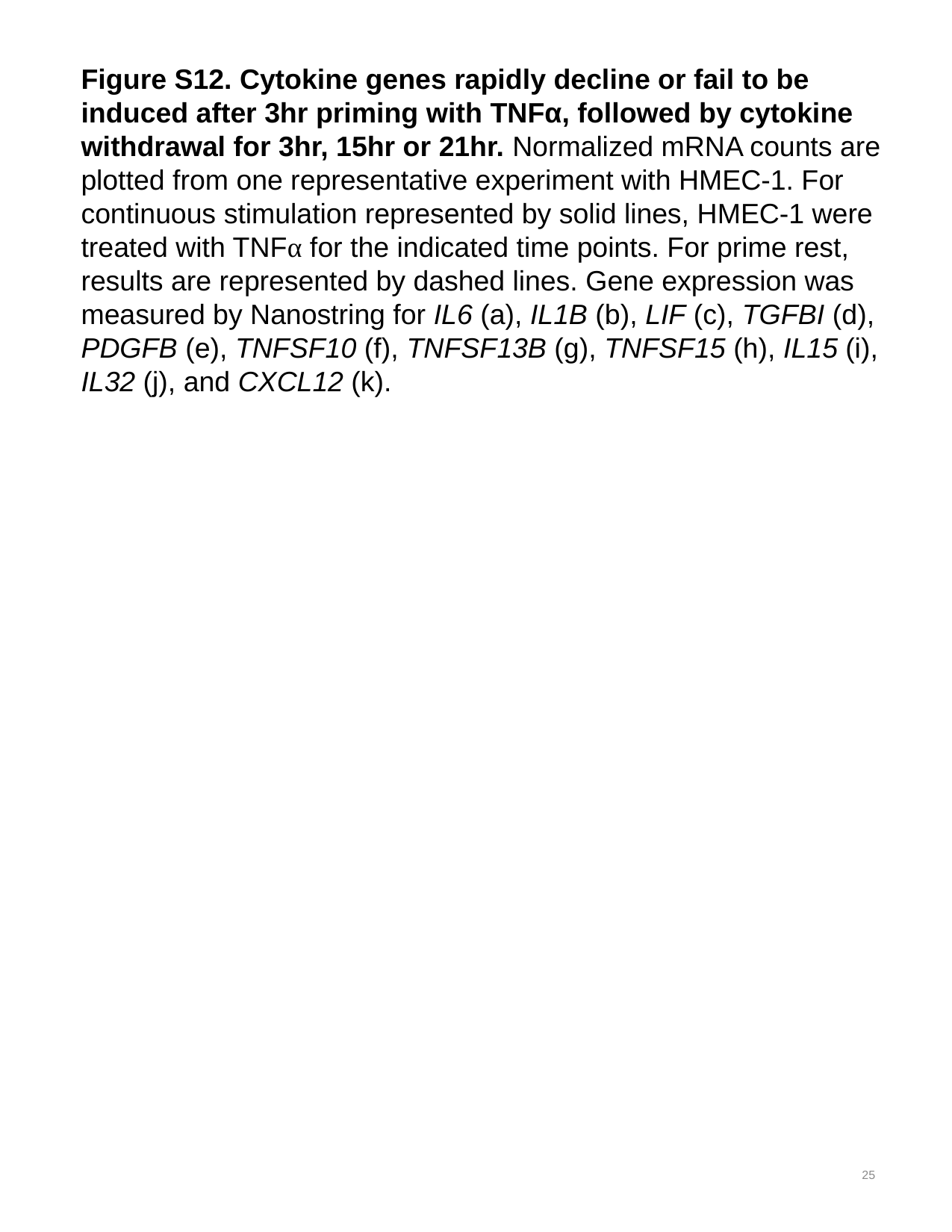

Figure S12. Cytokine genes rapidly decline or fail to be induced after 3hr priming with TNFα, followed by cytokine withdrawal for 3hr, 15hr or 21hr. Normalized mRNA counts are plotted from one representative experiment with HMEC-1. For continuous stimulation represented by solid lines, HMEC-1 were treated with TNFα for the indicated time points. For prime rest, results are represented by dashed lines. Gene expression was measured by Nanostring for IL6 (a), IL1B (b), LIF (c), TGFBI (d), PDGFB (e), TNFSF10 (f), TNFSF13B (g), TNFSF15 (h), IL15 (i), IL32 (j), and CXCL12 (k).
25

## Slide 26
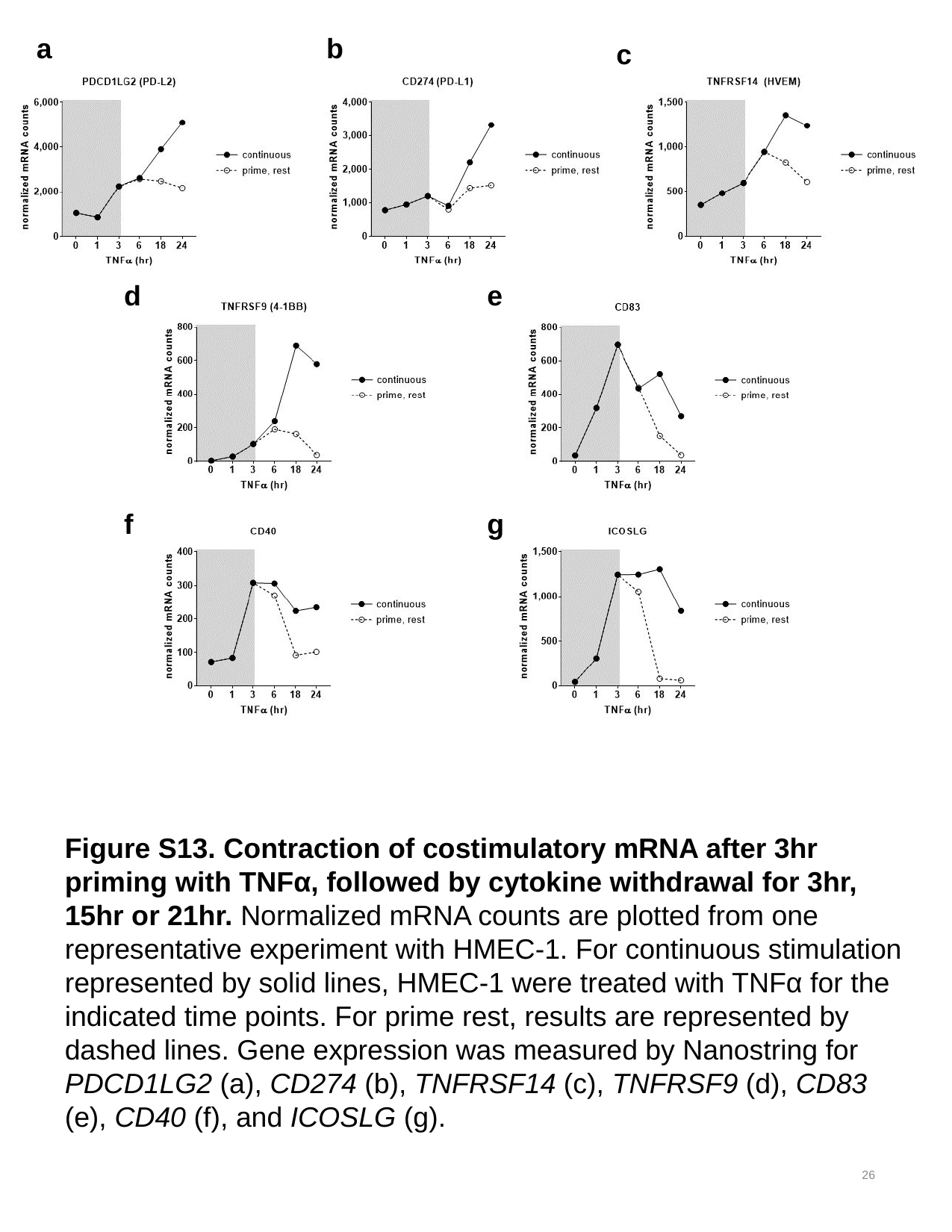

a
b
c
d
e
f
g
Figure S13. Contraction of costimulatory mRNA after 3hr priming with TNFα, followed by cytokine withdrawal for 3hr, 15hr or 21hr. Normalized mRNA counts are plotted from one representative experiment with HMEC-1. For continuous stimulation represented by solid lines, HMEC-1 were treated with TNFα for the indicated time points. For prime rest, results are represented by dashed lines. Gene expression was measured by Nanostring for PDCD1LG2 (a), CD274 (b), TNFRSF14 (c), TNFRSF9 (d), CD83 (e), CD40 (f), and ICOSLG (g).
26

## Slide 27
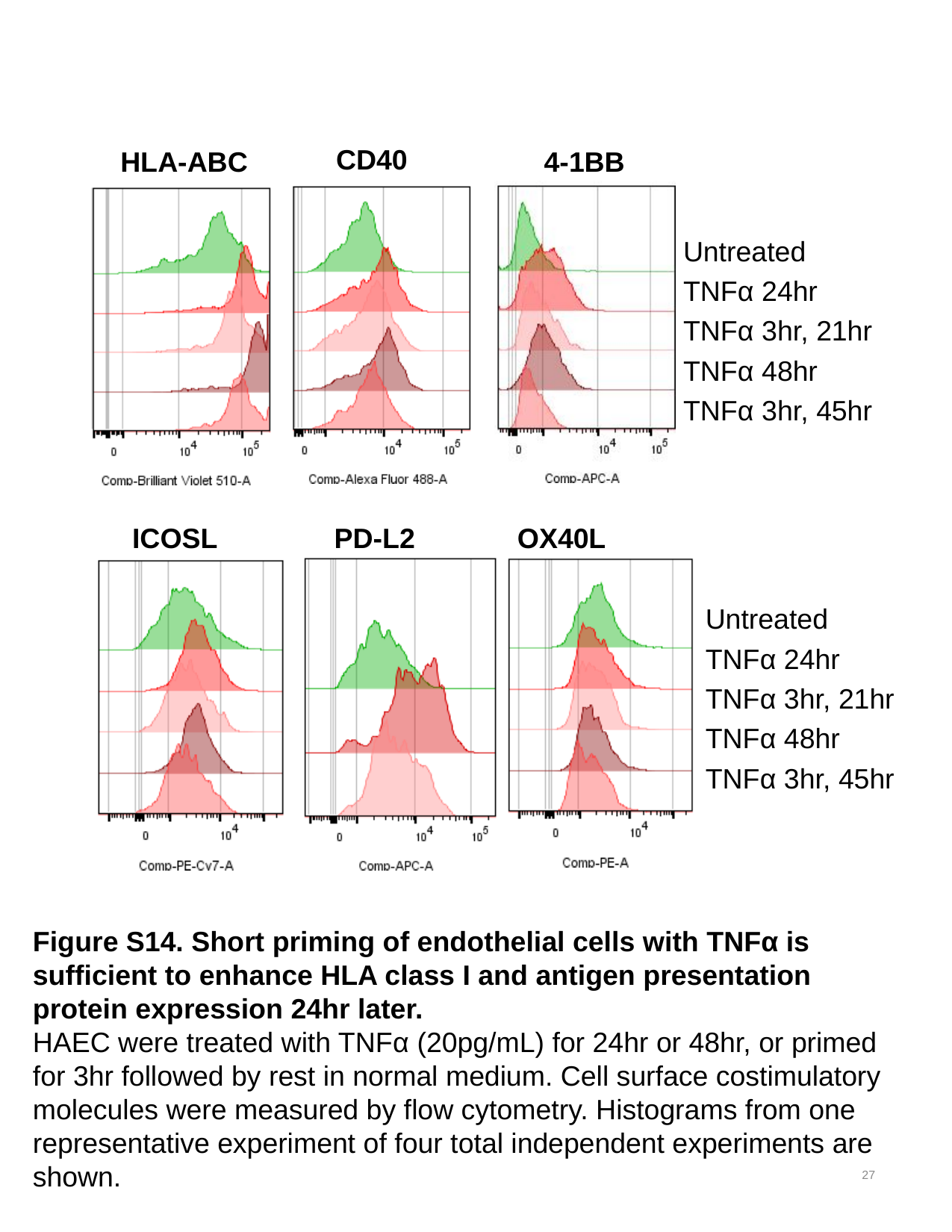

CD40
HLA-ABC
4-1BB
Untreated
TNFα 24hr
TNFα 3hr, 21hr
TNFα 48hr
TNFα 3hr, 45hr
ICOSL
PD-L2
OX40L
Untreated
TNFα 24hr
TNFα 3hr, 21hr
TNFα 48hr
TNFα 3hr, 45hr
Figure S14. Short priming of endothelial cells with TNFα is sufficient to enhance HLA class I and antigen presentation protein expression 24hr later.
HAEC were treated with TNFα (20pg/mL) for 24hr or 48hr, or primed for 3hr followed by rest in normal medium. Cell surface costimulatory molecules were measured by flow cytometry. Histograms from one representative experiment of four total independent experiments are shown.
27

## Slide 28
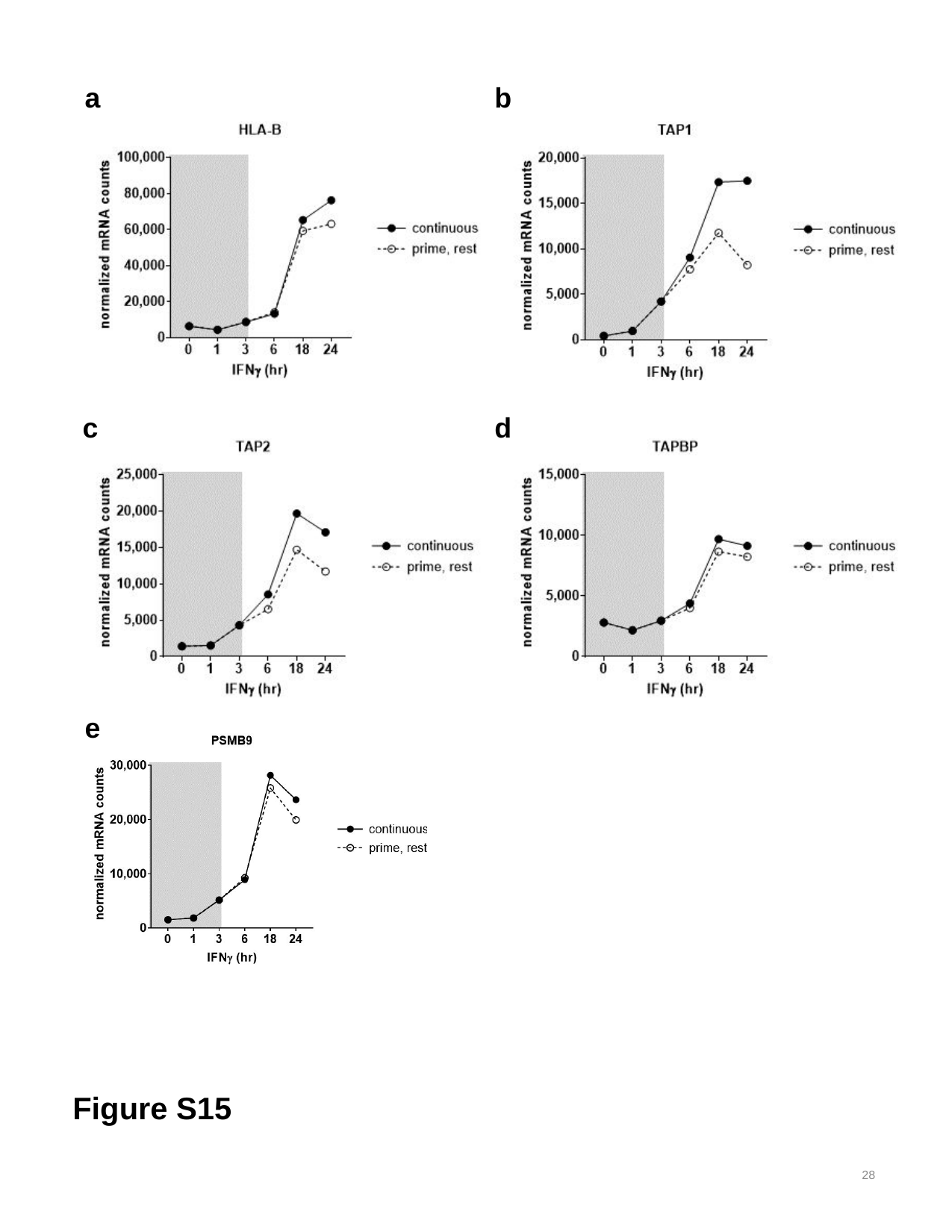

a
b
c
d
e
Figure S15
28

## Slide 29
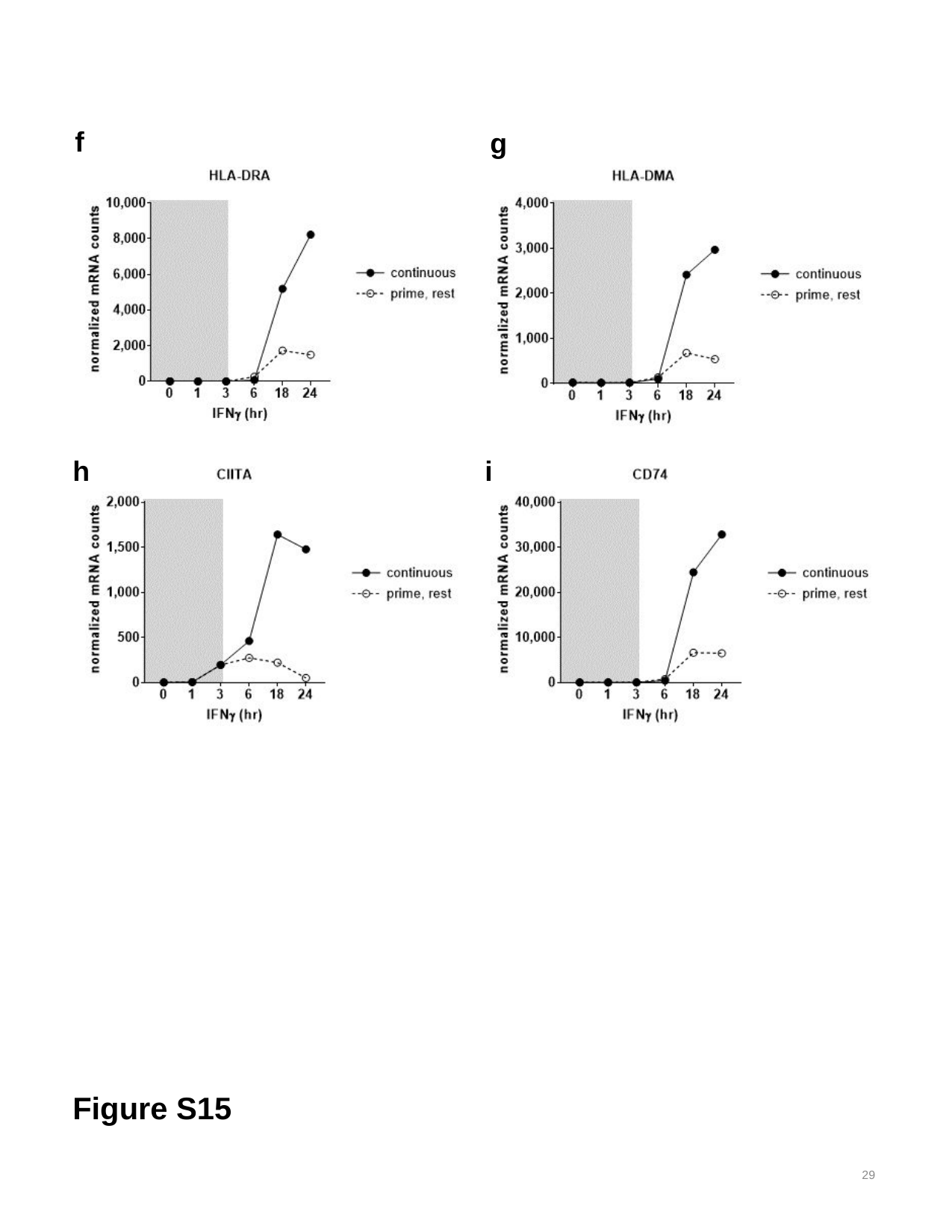

f
g
h
i
Figure S15
29

## Slide 30
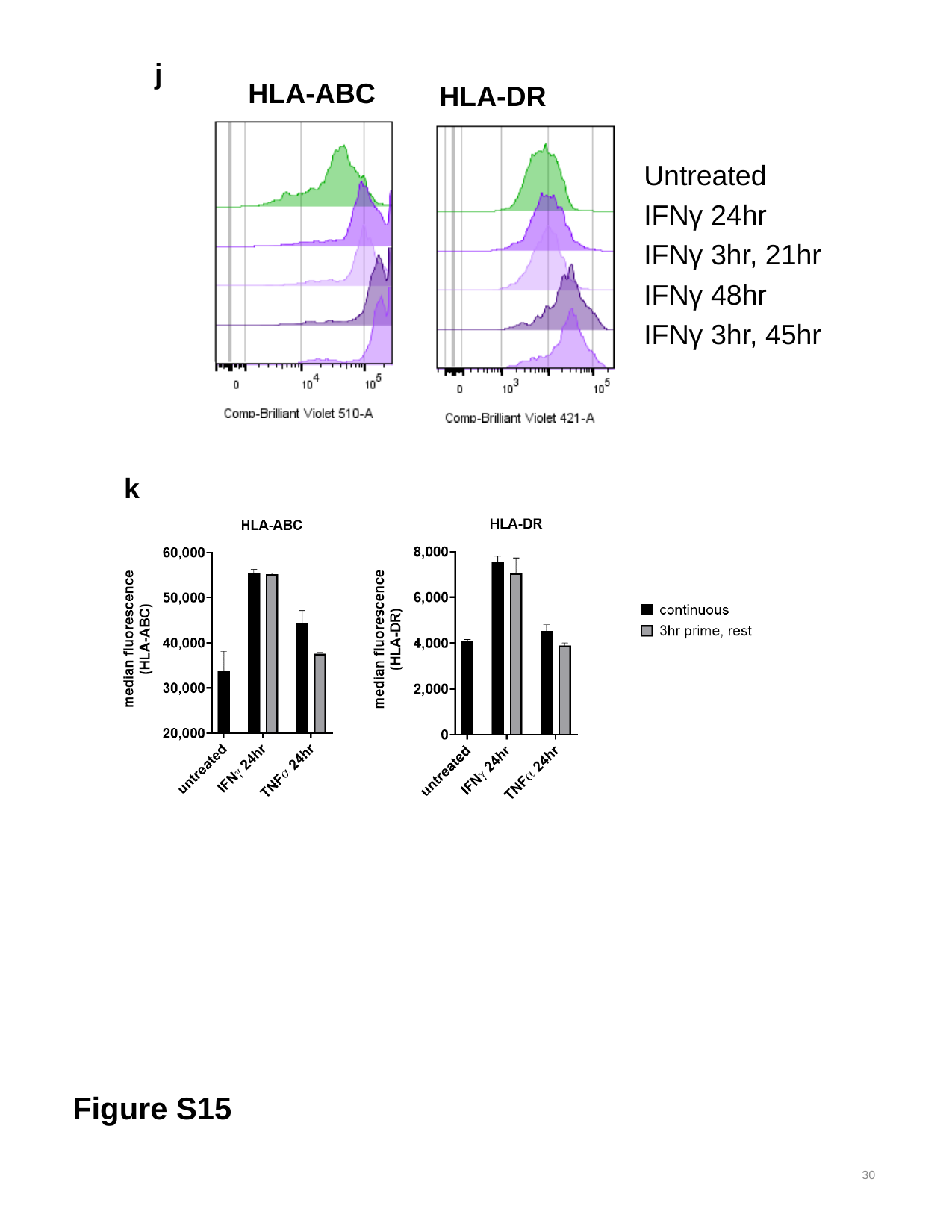

j
HLA-ABC
HLA-DR
Untreated
IFNγ 24hr
IFNγ 3hr, 21hr
IFNγ 48hr
IFNγ 3hr, 45hr
k
Figure S15
30

## Slide 31
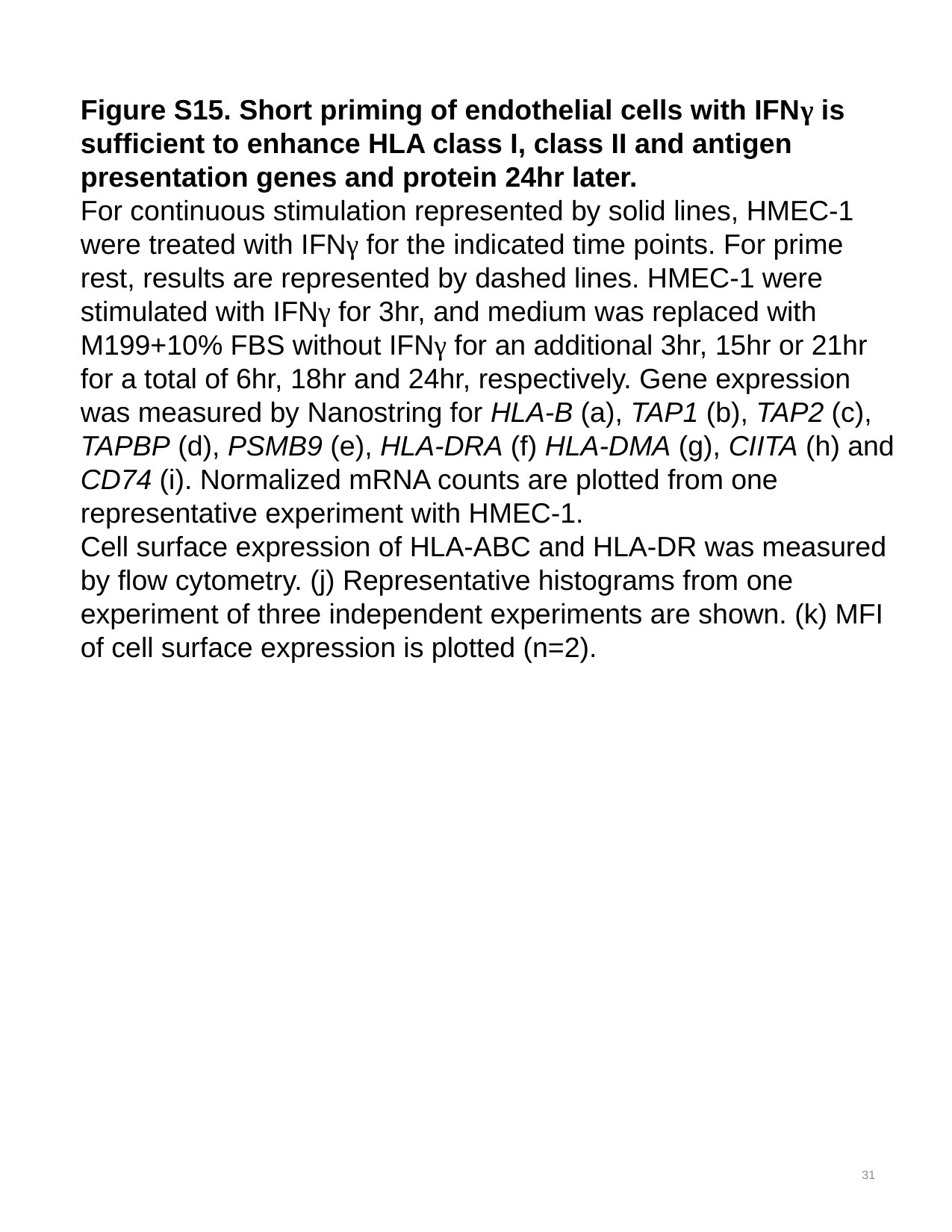

Figure S15. Short priming of endothelial cells with IFNγ is sufficient to enhance HLA class I, class II and antigen presentation genes and protein 24hr later.
For continuous stimulation represented by solid lines, HMEC-1 were treated with IFNγ for the indicated time points. For prime rest, results are represented by dashed lines. HMEC-1 were stimulated with IFNγ for 3hr, and medium was replaced with M199+10% FBS without IFNγ for an additional 3hr, 15hr or 21hr for a total of 6hr, 18hr and 24hr, respectively. Gene expression was measured by Nanostring for HLA-B (a), TAP1 (b), TAP2 (c), TAPBP (d), PSMB9 (e), HLA-DRA (f) HLA-DMA (g), CIITA (h) and CD74 (i). Normalized mRNA counts are plotted from one representative experiment with HMEC-1.
Cell surface expression of HLA-ABC and HLA-DR was measured by flow cytometry. (j) Representative histograms from one experiment of three independent experiments are shown. (k) MFI of cell surface expression is plotted (n=2).
31

## Slide 32
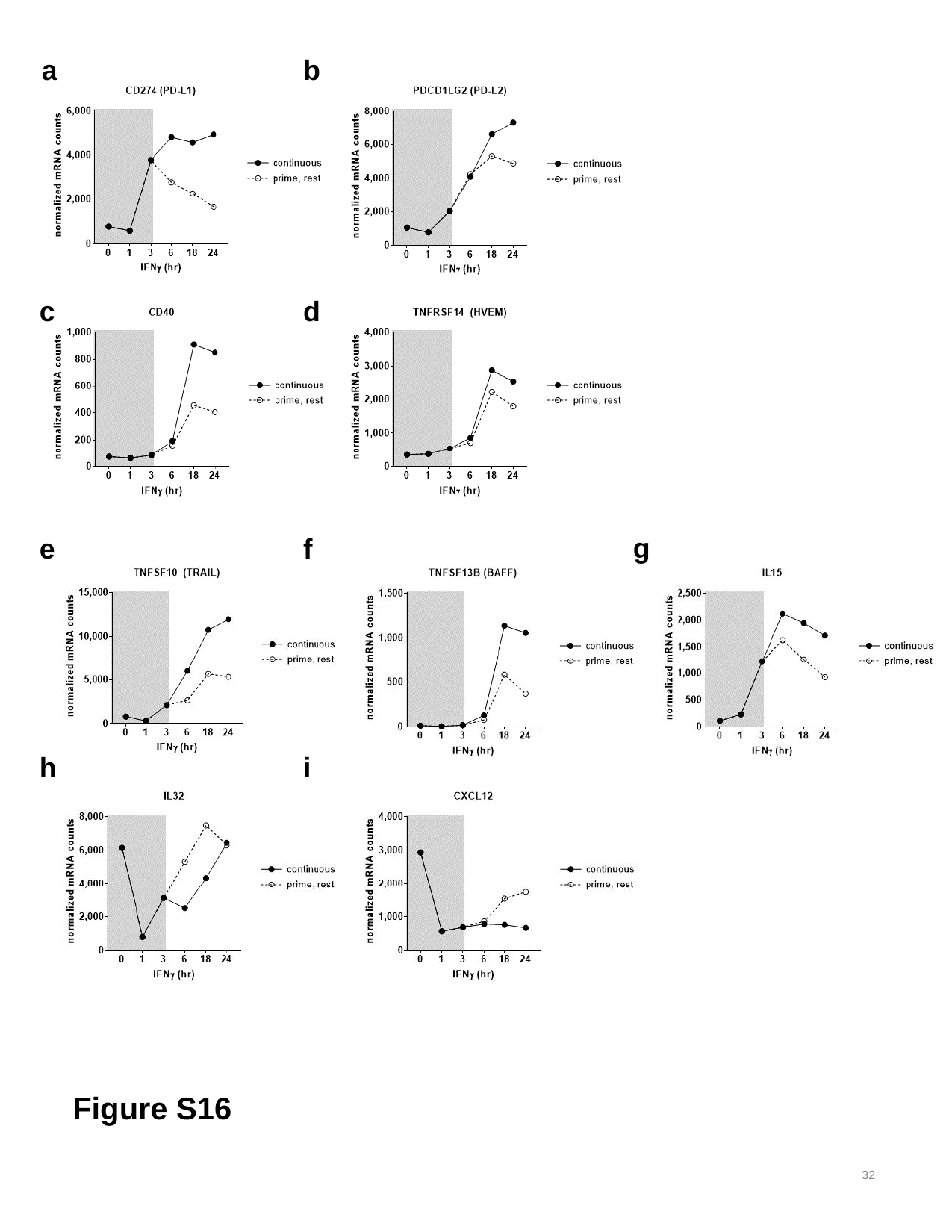

a
b
c
d
g
e
f
h
i
Figure S16
32

## Slide 33
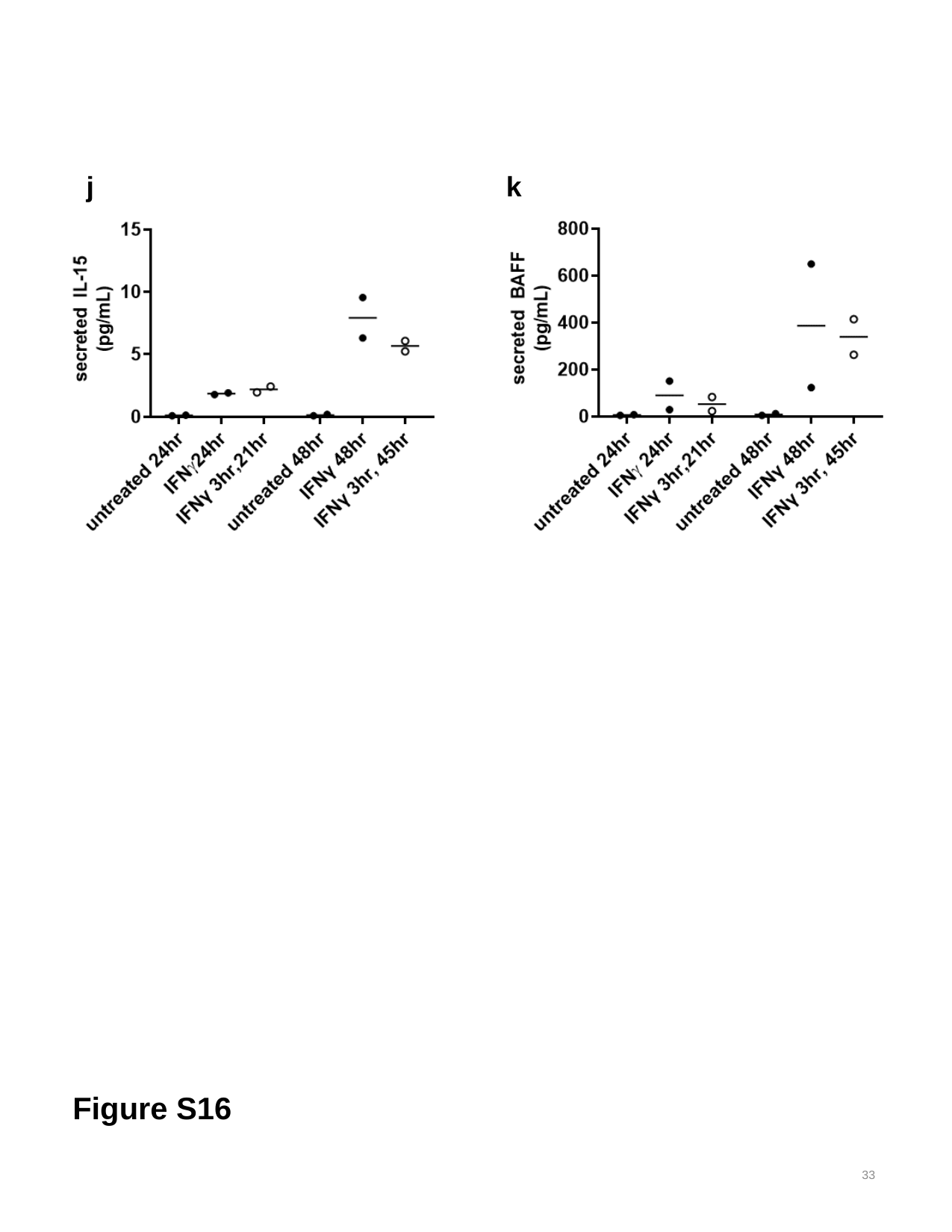

j
k
Figure S16
33

## Slide 34
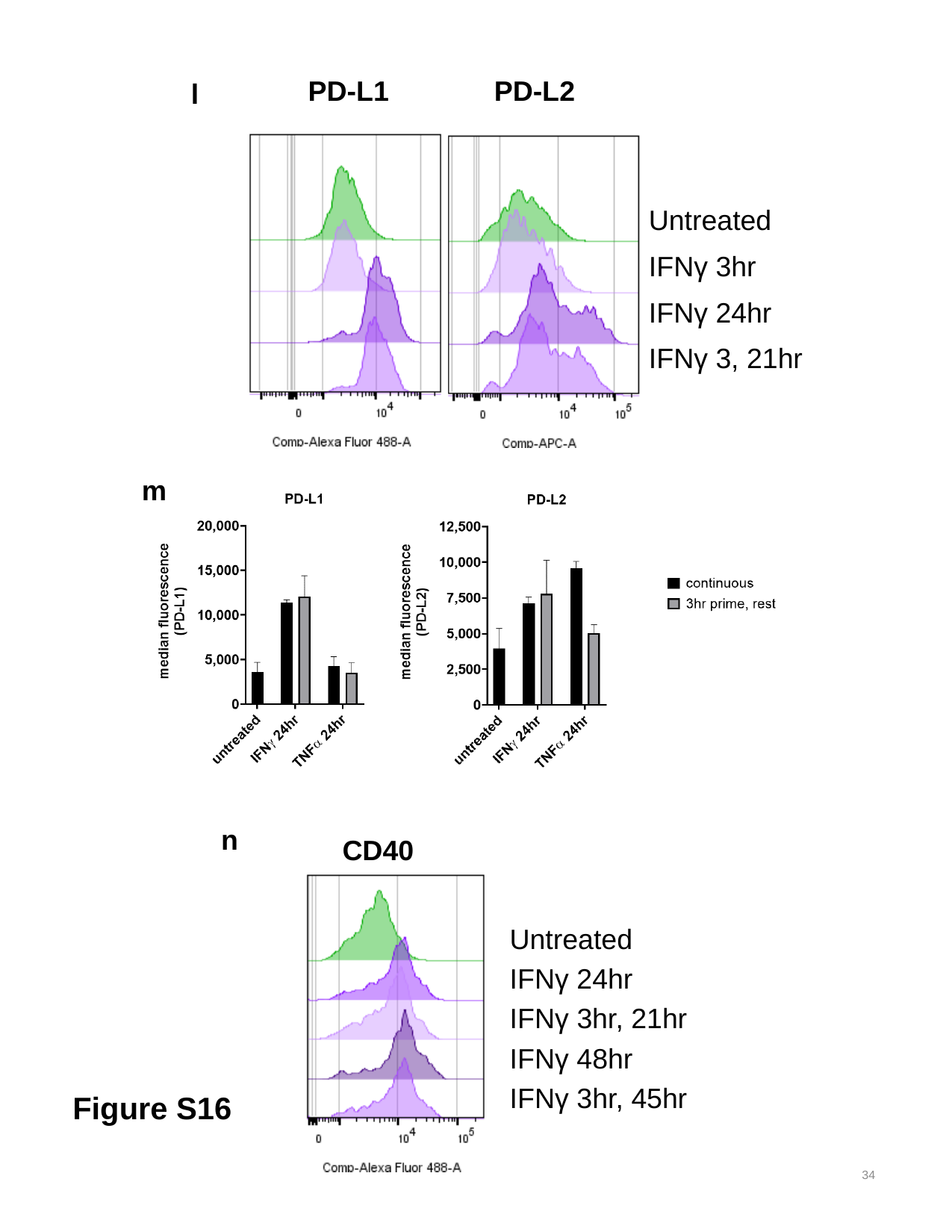

PD-L1
PD-L2
l
Untreated
IFNγ 3hr
IFNγ 24hr
IFNγ 3, 21hr
m
n
CD40
Untreated
IFNγ 24hr
IFNγ 3hr, 21hr
IFNγ 48hr
IFNγ 3hr, 45hr
Figure S16
34

## Slide 35
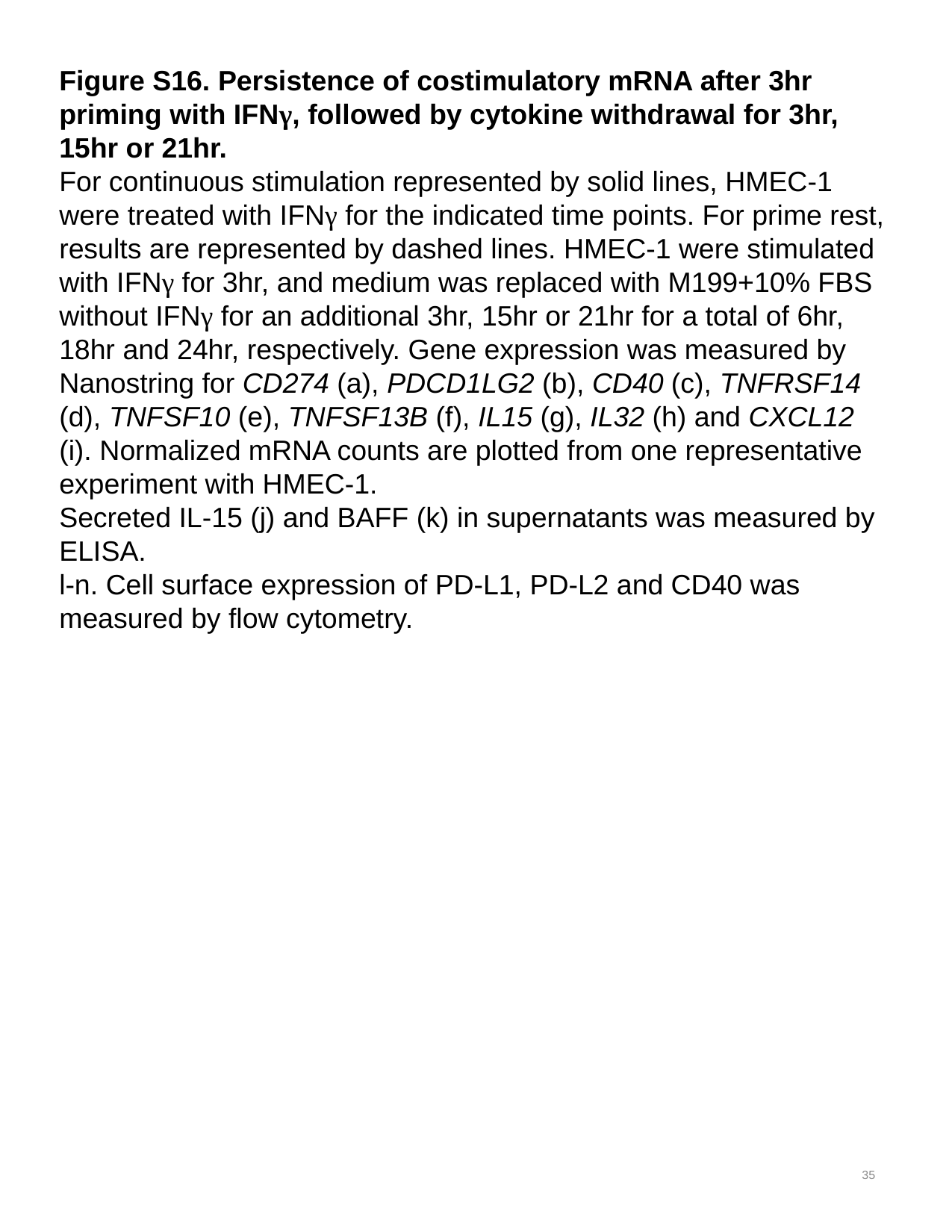

Figure S16. Persistence of costimulatory mRNA after 3hr priming with IFNγ, followed by cytokine withdrawal for 3hr, 15hr or 21hr.
For continuous stimulation represented by solid lines, HMEC-1 were treated with IFNγ for the indicated time points. For prime rest, results are represented by dashed lines. HMEC-1 were stimulated with IFNγ for 3hr, and medium was replaced with M199+10% FBS without IFNγ for an additional 3hr, 15hr or 21hr for a total of 6hr, 18hr and 24hr, respectively. Gene expression was measured by Nanostring for CD274 (a), PDCD1LG2 (b), CD40 (c), TNFRSF14 (d), TNFSF10 (e), TNFSF13B (f), IL15 (g), IL32 (h) and CXCL12 (i). Normalized mRNA counts are plotted from one representative experiment with HMEC-1.
Secreted IL-15 (j) and BAFF (k) in supernatants was measured by ELISA.
l-n. Cell surface expression of PD-L1, PD-L2 and CD40 was measured by flow cytometry.
35
